# Analysing Recent Socioeconomic Trends in Coronary Heart Disease Mortality in England, 2000–2007: A Population Modelling Study

**DOI:** 10.1371/journal.pmed.1001237

**Published:** 2012-06-12

**Authors:** Madhavi Bajekal, Shaun Scholes, Hande Love, Nathaniel Hawkins, Martin O'Flaherty, Rosalind Raine, Simon Capewell

**Affiliations:** 1 Department of Applied Health Research, University College London, London, United Kingdom; 2 Pensions and Annuity Group, Legal and General Assurance Society Limited, Kingswood, United Kingdom; 3 Institute of Cardiovascular Medicine & Science, Liverpool Heart and Chest Hospital, Liverpool, United Kingdom; 4 Institute of Psychology, Health and Society, University of Liverpool, Liverpool, United Kingdom; University of Queensland, Australia

## Abstract

**Background:**

Coronary heart disease (CHD) mortality in England fell by approximately 6% every year between 2000 and 2007. However, rates fell differentially between social groups with inequalities actually widening. We sought to describe the extent to which this reduction in CHD mortality was attributable to changes in either levels of risk factors or treatment uptake, both across and within socioeconomic groups.

**Methods and Findings:**

A widely used and replicated epidemiological model was used to synthesise estimates stratified by age, gender, and area deprivation quintiles for the English population aged 25 and older between 2000 and 2007. Mortality rates fell, with approximately 38,000 fewer CHD deaths in 2007. The model explained about 86% (95% uncertainty interval: 65%–107%) of this mortality fall. Decreases in major cardiovascular risk factors contributed approximately 34% (21%–47%) to the overall decline in CHD mortality: ranging from about 44% (31%–61%) in the most deprived to 29% (16%–42%) in the most affluent quintile. The biggest contribution came from a substantial fall in systolic blood pressure in the population not on hypertension medication (29%; 18%–40%); more so in deprived (37%) than in affluent (25%) areas. Other risk factor contributions were relatively modest across all social groups: total cholesterol (6%), smoking (3%), and physical activity (2%). Furthermore, these benefits were partly negated by mortality increases attributable to rises in body mass index and diabetes (−9%; −17% to −3%), particularly in more deprived quintiles. Treatments accounted for approximately 52% (40%–70%) of the mortality decline, equitably distributed across all social groups. Lipid reduction (14%), chronic angina treatment (13%), and secondary prevention (11%) made the largest medical contributions.

**Conclusions:**

The model suggests that approximately half the recent CHD mortality fall in England was attributable to improved treatment uptake. This benefit occurred evenly across all social groups. However, opposing trends in major risk factors meant that their net contribution amounted to just over a third of the CHD deaths averted; these also varied substantially by socioeconomic group. Powerful and equitable evidence-based population-wide policy interventions exist; these should now be urgently implemented to effectively tackle persistent inequalities.

*Please see later in the article for the Editors' Summary*

## Abstract 2

A modeling study conducted by Madhavi Bajekal and colleagues estimates the extent to which specific risk factors and changes in uptake of treatment contributed to the declines in coronary heart disease mortality in England between 2000 and 2007, across and within socioeconomic groups.

## Editors' Summary

**Background:**

Coronary heart disease is a chronic medical condition in which the blood vessels supplying the heart muscle become narrowed or even blocked by fatty deposits on the inner linings of the blood vessels—a process known as arthrosclerosis; this restricts blood flow to the heart, and if the blood vessels completely occlude, it may cause a heart attack. Lifestyle behaviors, such as unhealthy diets high in saturated fat, smoking, and physical inactivity, are the main risk factors for coronary heart disease, so efforts to reduce this condition are directed towards these factors. Global rates of coronary heart disease are increasing and the World Health Organization estimates that by 2030, it will be the biggest cause of death worldwide. However, in high-income countries, such as England, deaths due to coronary heart disease have actually fallen substantially over the past few decades with an accelerated reduction in annual death rates since 2000.

**Why Was This Study Done?:**

Socioeconomic factors play an important role in chronic diseases such as coronary heart disease, with mortality rates almost twice as high in deprived than affluent areas. However, the potential effect of population-wide interventions on reducing inequalities in deaths from coronary heart disease remains unclear. So in this study, the researchers investigated the role of behavioral (changing lifestyle) and medical (treatments) management of coronary heart disease that contributed to the decrease in deaths in England for the period 2000–2007, within and between socioeconomic groups.

**What Did the Researchers Do and Find?:**

The researchers used a well-known, tried and tested epidemiological model (IMPACT) but adapted it to include socioeconomic inequalities to analyze the total population of England aged 25 and older in 2000 and in 2007. The researchers included all the major risk factors for coronary heart disease plus 45 current medical and surgical treatments in their model. They used the Index of Multiple Deprivation 2007 as a proxy indicator of socioeconomic circumstances of residents in neighborhoods. Using the postal code of residence, the researchers matched deaths from, and patients treated for, coronary heart disease to the corresponding deprivation category (quintile). Changes in risk factor levels in each quintile were also calculated using the Health Survey for England. Using their model, the researchers calculated the total number of deaths prevented or postponed for each deprivation quintile by measuring the difference between observed deaths in 2007 and expected deaths based on 2000 data, if age, sex, and deprivation quintile death rates had remained the same.

The researchers found that between 2000 and 2007, death rates from coronary heart disease fell from 229 to 147 deaths per 100,000—a decrease of 36%. Both death rates and the number of deaths were lowest in the most affluent quintile and the pace of fall was also faster, decreasing by 6.7% per year compared to just 4.9% in the most deprived quintile. Furthermore, the researchers found that overall, about half of the decrease in death rates was attributable to improvements in uptake of medical and surgical treatments. The contribution of medical treatments to the deaths averted was very similar across all quintiles, ranging from 50% in the most affluent quintile to 53% in the most deprived. Risk factor changes accounted for approximately a third fewer deaths in 2007 than occurred in 2000, but were responsible for a smaller proportion of deaths prevented in the most affluent quintile compared with the most deprived (approximately 29% versus 44%, respectively). However, the benefits of improvements in blood pressure, cholesterol, smoking, and physical activity were partly negated by rises in body mass index and diabetes, particularly in more deprived quintiles.

**What Do These Findings Mean?:**

These findings suggest that approximately half the recent substantial fall in deaths from coronary heart disease in England was attributable to improved treatment uptake across all social groups; this is consistent with equitable service delivery across the UK's National Health Service. However, opposing trends in major risk factors, which varied substantially by socioeconomic group, meant that their net contribution accounted for just a third of deaths averted. Other countries have implemented effective, evidence-based interventions to tackle lifestyle risk factors; the most powerful measures involve legislation, regulation, taxation, or subsidies, all of which tend to be equitable. Such measures should be urgently implemented in England to effectively tackle persistent inequalities in deaths due to coronary heart disease.

**Additional Information:**

Please access these Web sites via the online version of this summary at http://dx.doi.org/10.1371/journal.pmed.1001237.

- The World Health Organization has information about the global statistics of coronary heart disease
- The National Heart Lung and Blood Institute provides a patient-friendly description of coronary heart disease
- The National Heart Forum is the leading UK organization facilitating the prevention of coronary heart disease and other chronic diseases
- The British Heart Foundation supports research and promotes preventative activity
- Heart of Mersey is the UK's largest regional organization promoting the prevention of coronary heart disease and other chronic diseases
- More information about the social determinants of health is available from WHO

## Introduction

Since the 1970s, coronary heart disease (CHD) mortality in England has fallen by a remarkable 60%, with accelerated reductions in annual death rates since 2000 [1]. However, CHD remains the leading cause of mortality and is a major contributor to social inequalities in premature mortality in England, as in the USA [2],[3]. Moreover, UK death rates have fallen faster in the most socially advantaged groups compared to the most deprived [1]. Thus, whilst absolute inequalities in mortality have fallen, relative inequalities have increased over the last decade. Previous country-level analyses have shown that about 50%–70% of the dramatic falls in CHD mortality between 1980 and 2000 were explained by improvements in modifiable risk factors (mainly smoking, total cholesterol and blood pressure), with the remaining 30%–50% attributable to improved uptake of evidence-based treatments [4]–[6]. However, so far no study has examined the specific contribution of risk factors and medical treatments to the underlying social differentials in CHD mortality falls.

The most recent study in the UK modelled CHD mortality change in England and Wales between 1981 and 2000 [4]. Since then several initiatives have been rolled out to improve the delivery of health care in England. Notable among them include the National Service Framework for CHD (2000) and the Qualities and Outcome Framework (2004), which aim to monitor and incentivise improvements in the quality of services provided for CHD prevention, diagnosis, treatment, and rehabilitation [7],[8]. In addition, important population-wide public health measures to reduce risk factors across the entire population were introduced. These measures included the ban on tobacco advertising (2003); comprehensive smoke-free legislation (2007), and voluntary agreements to reduce salt and artificial trans-fats in processed food [9],[10]. Reducing health inequalities was at the heart of New Labour's health agenda when it came to office in 1997. However, the target to reduce the inequality gap in life expectancy by 2010 was not met [11]. Furthermore, the potential effect of population-wide interventions on reducing inequalities in CHD mortality (when compared with individual treatments) remains unclear [12].

Thus although our analysis covers a relatively short period of time, the period included a raft of measures specifically aimed to improve outcomes and reduce social inequalities. We have quantified the variation by socioeconomic circumstances (SEC) in the relative contributions of modifiable population-level risk factors and evidence-based individual treatments to the fall in CHD mortality during the period 2000 to 2007. To do this we have used the widely used and replicated IMPACT model, substantially extending the model to capture socioeconomic inequalities concealed within the overall national trends.

## Methods

### IMPACT_SEC_ Model and Data Sources

IMPACT is an epidemiological model used to explain the contributions of population-level risk factor changes (incidence reduction) and uptake of evidence-based treatments (case fatality reduction) to the change in CHD deaths between two points in time. This deterministic, cell-based model has been described in detail elsewhere [4],[5]. The extended IMPACT_SEC_ model included all the major risk factors for CHD: smoking, systolic blood pressure, total cholesterol, body mass index (BMI), diabetes, physical inactivity, along with fruit and vegetable consumption; plus all 45 medical and surgical treatments currently in use in nine patient groups. The model included the total population of England aged 25 y and over in 2000 and 2007.

Data sources specific to the England population were used to construct the IMPACT_SEC_ model. When several sources were available, we chose the most up-to-date, representative dataset that we could link to a small-area deprivation index. Population estimates and CHD death counts (2000: ICD9 410–414; 2007: ICD10 I20–I25) by sex, 5-y age bands to age 85+, and deprivation quintile were obtained from the Office for National Statistics. Emergency admissions for acute myocardial infarction were extracted from Hospital Episode Statistics and supplemented with data from the Myocardial Ischaemia National Audit Project to disaggregate ST-elevated acute myocardial infarction and non–ST-elevated acute coronary syndrome, and to apportion treatment uptake to each group. For heart failure admissions, the National Health Service (NHS) Heart Failure Survey was used to estimate in-hospital treatment uptake. The General Practice Research Database and the Health Survey for England provided data on treatment uptake in the community. Risk factor trend data came from the Health Survey for England.

Detailed information on the IMPACT_SEC_ model, calculation methods, and data sources are provided as supporting information in Text S1.

### Stratifying Data according to Socioeconomic Circumstances

Only the Health Survey for England consistently recorded individual socioeconomic position; but all data sources recorded individual's postcode of residence. We therefore used a measure of relative area deprivation as a proxy indicator of the SECs of individuals living in small areas (*n* = 32,482; average population of 1,500). We used the Index of Multiple Deprivation 2007 [13] to rank all lower super output areas in England in ascending order of increasing deprivation and grouped them into equal quintiles. Although scoring low on the deprivation index does not necessarily equate to affluence, to assist readability we refer to quintile one as “most affluent” and quintile five as “most deprived.” On the basis of postcode of residence, the data providers matched CHD deaths and treated patients to their corresponding deprivation quintile before releasing the data to us.

### Deaths Prevented or Postponed

The total number of deaths prevented or postponed (DPPs) for each deprivation quintile were calculated as the difference between observed deaths in 2007 and expected deaths had age-, sex-, and quintile- specific CHD mortality rates in 2000 remained unchanged. DPPs explained by the model could be positive (i.e., deaths averted) or negative (i.e., additional deaths in 2007 relative to 2000). Any shortfalls between the DPPs explained by the model and the total DPPs for each SEC quintile were assumed to reflect either imprecision in our model parameters or omission of other, unmeasured risk factors.

### Mortality Reductions Attributable to Treatment Uptake

The treatment component of IMPACT_SEC_ included nine mutually exclusive CHD patient groups: ST-elevation myocardial infarction; non–ST-elevation acute coronary syndrome; secondary prevention post myocardial infarction; secondary prevention post revascularisation; chronic stable coronary artery disease; heart failure patients admitted to hospital; heart failure patients resident in the community; persons in the community taking blood pressure lowering medication; and persons in the community taking lipid lowering treatment. A total of 45 patient-treatment pairings were generated. To avoid double counting of patients treated for two or more conditions within the year (e.g., heart failure develops within 1 y after myocardial infarction in approximately 30% of survivors), we quantified overlaps between different groups and made appropriate adjustments to construct distinct, non-overlapping CHD patient subgroups (Text S1, Table N).

The numbers eligible for treatment, uptake of specific treatment, 1-y case fatality rates, and relative risk reduction due to treatment, all stratified by age, sex, and CHD patient subgroups, were extracted from relevant data sources (Text S1, Tables A, B, F, G, I, J). Disease prevalence and treatment uptake were further stratified by deprivation quintiles.

The estimates of medication uptake in the community recorded in our primary data sources were adjusted to reflect ordinary clinical practice. We assumed that medication adherence (i.e., the proportion of eligible patients actually taking therapeutically effective levels of medication) was 100% among hospitalised patients, 70% among symptomatic patients in the community, and 50% among asymptomatic patients in the community [5]. The scale of the downward adjustment of uptake was assumed to reasonably represent the combined effects of lower rates of adherence in community settings and reduced population effectiveness in routine health care settings compared to optimal clinical trial conditions. Uncertainty in both of these input variables—the actual uptake rates and the adjustment factor—were included in the stochastic sensitivity analysis reported below.

Deaths prevented by each intervention were then calculated by multiplying the numbers of patients in each diagnostic group by the (adherence-adjusted) proportion of those patients who received the treatment, the baseline case fatality rate, and the relative risk reduction of that treatment. To estimate the cumulative effect of relative risk reduction for patients on a combination of drug therapies, we used the Mant and Hicks correction [14].

Many of the treatments were already widely used in 2000. The net benefit of an intervention in 2007 was therefore calculated by subtracting the expected number of deaths prevented if 2000 uptake rates had remained unchanged from the deaths prevented using 2007 treatment uptake rates.

### Mortality Reductions Attributable to Risk Factor Changes

We included seven risk factors in the model; both behavioural—smoking, physical inactivity, fruit and vegetable consumption, BMI—and physiological markers including systolic blood pressure, total serum cholesterol, and diagnosed diabetes. To quantify the mortality benefits of an absolute change in each specific risk factor between 2000 and 2007, we used two approaches: a regression-based approach for factors measured on a continuous scale (e.g., total blood cholesterol); and, a population-attributable risk fraction approach for dichotomous variables such as diagnosed diabetes. The independent regression coefficients of mortality benefit for a unit change in mean risk factor were obtained from published multivariate analyses (Text S1, Table I). Hence, the contribution of each risk factor to deaths averted was then calculated as the product of the deaths in 2000 (the base year), the absolute change in risk factor, and the associated relative risk reduction. For binary variables, we used relative risks from the most recent meta-analyses and population cohort studies (Text S1, Table J).

CHD deaths are usually caused by multiple risk factors acting simultaneously. It is therefore recommended that enumerating mortality benefits from risk factor reductions should not use a simple additive approach. Instead, the effects of risk factor changes should be jointly estimated using the cumulative risk-reduction approach. This can be stated in the equation:
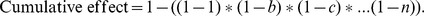
The cumulative effect of change in all risk factors over the study period was calculated by age, sex, and deprivation quintile (see Text S1, Section 1.3). The ratio of the cumulative effect to the corresponding additive effect was then calculated, yielding 70 adjustment factors (Text S1, Table D). These adjustment factors were used to scale down the additive DPPs for each risk factor. These adjusted DPPs, summed over all seven risk factors, then equalled the estimated total combined DPPs, capturing the multiplicative net impact of positive and adverse changes in risk factors. All risk factor DPPs quoted in the results tables refer to the adjusted DPPs.

We assumed that there was no further synergy between the treatment and risk-factor components of the model. Lag times between the change in cardiovascular risk factor levels and change in CHD mortality rates were assumed to be relatively rapid [15] and were therefore not specifically modelled. Similar to economic evaluation studies in health [16], 95% uncertainty intervals around the model output (i.e., DPPs) were calculated using Monte Carlo simulation. This calculation involved replacing all fixed input parameters used in the model by appropriate probability distributions, and repeatedly recalculating the model output with values sampled from the defined input distributions (Text S1, Table M). We used the EXCEL add-in Ersatz software (www.epigear.com) to perform 1,000 runs to determine the 95% uncertainty intervals of the DPPs (2.5th and 97.5th percentile values corresponding to the lower and upper limits).

## Results

Between 2000 and 2007, the age-standardised CHD mortality rate in adults aged 25 y and over fell from 229 to 147 deaths per 100,000; a decline of 36% overall or 6.1% per year (Table 1). In 2007, there were 74,174 CHD deaths, 56% of these were in men. Both death rates and the number of deaths were lowest in the most affluent quintile and the pace of fall was also faster: decreasing by 6.7% per year compared to just 4.9% in the most deprived quintile. Differentials in the rates of fall therefore widened relative inequalities over the period.

**Table 1:** Observed coronary heart disease deaths, age-standardised rates 2000 and 2007 and deaths prevented or postponed in England and stratified by deprivation quintiles.

Adults Aged 25 and over	England	Most Affluent	IMDQ2	IMDQ3	IMDQ4	Most Deprived
Population (000s)						
2000	33,952	6,972	7,035	6,939	6,678	6,329
2007	35,281	7,328	7,363	7,233	6,906	6,451
Observed deaths						
2000	103,243	16,529	19,827	21,460	22,187	23,240
2007	74,174	12,312	14,444	15,347	15,676	16,395
Age-standardised rates (per 100,000)						
2000	229	177	199	222	257	306
2007	147	109	124	141	169	215
Annual percent fall	6.1	6.7	6.5	6.3	5.8	4.9
Expected deaths 2007 (had 2000 rates persisted)	112,244	19,665	22,669	23,696	23,260	22,953
DPP (Expected - observed deaths, 2007)	38,070	7,353	8,225	8,349	7,584	6,558
Percent expected deaths averted	33.9	37.4	36.3	35.2	32.6	28.6

Rates have been standardised to the European Union reference population aged 25 and over. Separate breakdowns for males and females are available in Text S1, Table E.

IMD, index of multiple deprivation.

Nationally, there were 38,070 fewer CHD deaths in 2007 than if 2000 mortality rates had persisted, representing the “total” DPPs. Despite the slower annual rates of fall in the most deprived quintile, their higher CHD mortality rates in the base year meant that the number of DPPs by 2007 were fairly equally distributed: about 6,560 fewer deaths in the most deprived quintile versus 7,355 in the most affluent (Table 1).

**Figure 1:**
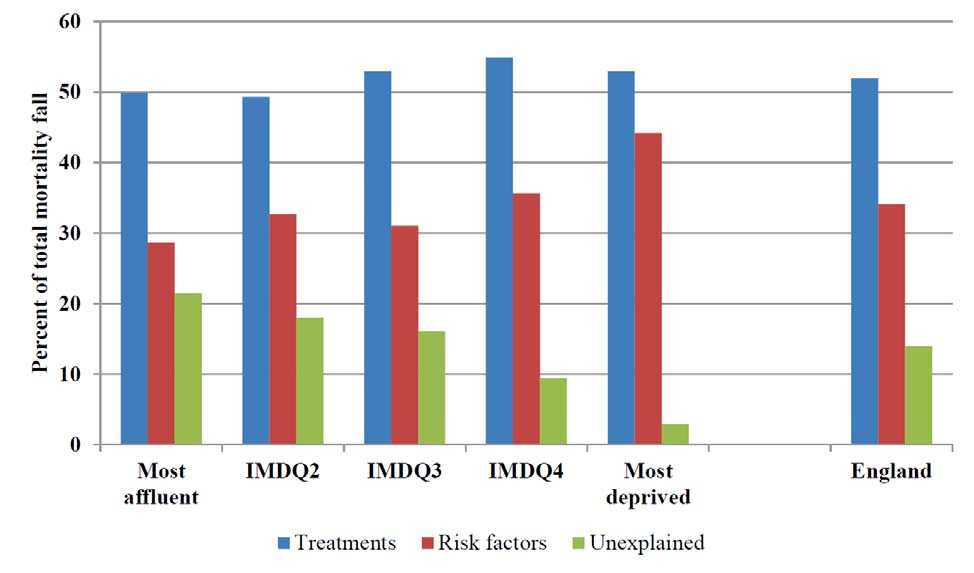
Proportion of coronary heart disease mortality decline attributable to treatment or risk factor changes, 2000–2007. England overall and by socioeconomic quintile.

Overall, approximately half of the total CHD mortality fall (19,780 fewer deaths or 52%; 95% uncertainly interval ranging from 40% to 70%) was attributable to improvements in uptake of medical and surgical treatments (Table 2). In contrast, population-level risk factor changes accounted for approximately 12,990 (34%; 21%–47%) fewer deaths (Table 3). The model could not explain some 14% of the overall mortality fall (i.e., a shortfall of 5,300 deaths) (Figure 1; Table 3). The contribution of medical treatments to the deaths averted was very similar across all quintiles, ranging from 50% in the most affluent quintile to 53% in the most deprived (Table 4). But risk factor changes explained a smaller proportion of deaths prevented in the most affluent quintile compared with the most deprived (approximately 29% versus 44%, respectively). As a result, about 21% of CHD deaths prevented could not be explained by the model in the most affluent quintile. The proportion not explained fell successively with increasing deprivation to 3% of CHD deaths (190 deaths) not explained in the most deprived quintile.

**Table 2:** Coronary heart disease deaths prevented or postponed due to changes in treatment uptake between 2000 and 2007 in England and stratified by deprivation quintiles.

Treatment by Patient Groups	England: DPP				By IMD: DPP				
	*n*	Percent	Percent Lower limit^a^	Percent Upper limit^a^	*n* Most Affluent	*n* IMDQ2	*n* IMDQ3	*n* IMDQ4	*n* Most Deprived
**STEMI** b	**−130**	**−0.3**	***−0.8***	***0.5***	**−6**	**−27**	**−28**	**−31**	**−38**
Thrombolysis	−118				−22	−23	−21	−23	−29
Aspirin	24				5	4	5	3	7
B-blocker	4				1	0	1	1	2
ACE inhibitor or ARB	5				0	0	2	1	2
Clopidogrel	65				12	14	14	13	12
Primary PCI	139				30	28	28	27	25
Primary CABG	1				0	0	0	0	0
CPR in hospital^c^	−252				−33	−51	−57	−53	−57
**NSTEACS** b	**295**	**0.8**	***0.0***	***2.2***	**57**	**59**	**62**	**53**	**65**
Aspirin and heparin	341				54	68	68	63	88
Aspirin alone	−114				−15	−24	−21	−21	−31
PG IIB/IIIA inhibitors	−1				−2	−1	0	0	2
ACE inhibitor or ARB	44				6	8	9	10	10
B-blocker	27				5	5	6	5	6
Clopidogrel	203				36	40	46	41	40
CABG surgery	1				2	1	0	0	−1
PCI	54				11	10	12	12	10
CPR in hospital^c^	−259				−40	−48	−58	−55	−58
**Secondary prevention post MI** b	**3,510**	**9.2**	***7.2***	***11.4***	**640**	**775**	**746**	**711**	**639**
Aspirin	351				65	69	76	87	55
B-blocker	862				158	197	194	167	146
ACE inhibitor or ARB	903				166	200	190	175	172
Statin	1,303				241	280	269	268	245
Warfarin	92				8	30	17	15	21
Rehabilitation^d^	0				0	0	0	0	0
**Secondary prevention post revascularisation** b	**590**	**1.5**	***1.2***	***1.9***	**112**	**121**	**128**	**117**	**110**
Aspirin	45				10	10	11	8	7
B-blocker	154				29	31	34	31	30
ACE inhibitor or ARB	179				35	37	37	34	36
Statin	174				31	36	38	37	32
Warfarin	0				0	0	1	0	−1
Rehabilitation (post CABG)^d^	0				0	0	0	0	0
Rehabilitation (post PTCA)	36				7	7	7	7	7
**Chronic stable CAD** b	**4,835**	**12.7**	***9.6***	***16.8***	**851**	**1,007**	**1,015**	**1,016**	**955**
Aspirin in community	818				139	159	176	179	165
Statin in community	2,488				443	523	526	510	485
ACE inhibitor or ARB	1,292				241	281	268	261	241
CABG surgery	236				27	44	45	57	63
**Heart failure - hospital** b	**250**	**0.7**	***0.5***	***0.8***	**42**	**47**	**53**	**59**	**51**
ACE inhibitor	49				8	9	10	10	11
B-blocker	39				6	8	8	9	9
Spironolactone	37				6	7	8	8	8
Aspirin	126				21	22	27	32	23
**Heart failure - community** b	**3,335**	**8.8**	***7.3***	***10.4***	**564**	**689v**	**732**	**711**	**641**
ACE inhibitor or ARB	737				125	158	171	146	137
B-blocker	1,592				284	325	353	338	292
Spironolactone	617				105	134	120	129	129
Aspirin	389				50	72	88	97	83
**Hypertension treatment** b	**1,800**	**4.7**	***1.8***	***9.9***	**357**	**411**	**408**	**345**	**277**
**Hyperlipidemia treatment (statins)** b	**5,300**	**13.9**	***5.0***	***31.8***	**1,054**	**975**	**1,305**	**1,194**	**772**
**Total treatment contribution** b	**19,780**	**52.0**	***40.2***	***69.7***	**3,670**	**4,055**	**4,420**	**4,166**	**3,471**

Subtotals for England (column 1) have been rounded to nearest 5.

a The 95% uncertainty interval corresponds to the lower (2.5th percentile) and upper (97.5th percentile) limits of the uncertainty analysis. These are shown to indicate range around the central estimate of percent of DPPs explained (column 3).

b Sub-totals (in rows) for CHD patient groups.

c We have assumed no change in cardiopulmonary resuscitation (CPR) uptake in the community between 2000 and 2007. Their contribution to DPPs have therefore been set to zero.

d No change in uptake between 2000 and 2007.

Abbreviations: B-blocker, beta-blocker; CABG, coronary artery bypass graft; CAD, coronary artery disease; IMD, index of multiple deprivation; MI, myocardial infarction; NSTEACS, non-ST elevation acute coronary syndrome; PCI, percutaneous coronary intervention; PG, platelet glycoprotein; PTCA, percutaneous transluminal coronary angioplasty; STEMI, ST elevation myocardial infarction.

**Table 3:** CHD deaths prevented or postponed due to changes in risk factor prevalence between 2000 and 2007 in England and stratified by deprivation quintiles.

Risk Factors	England: DPP				By IMD: DPP				
	*n*	Percent	Percent Lower Limit^a^	Percent Upper Limit^a^	*n* Most Affluent	*n* IMDQ2	*n* IMDQ3	*n* IMDQ4	*n* Most Deprived
**Current smoking**	1,040	2.7	0.1	5.6	93	148	189	242	368
**Physical inactivity**	600	1.6	0.8	2.3	91	109	119	130	155
**Systolic blood pressure, mmHg** b	11,160	29.3	18.4	40.0	1,861	2,168	2,321	2,391	2,421
**Total cholesterol, mmol/l** c	2,090	5.5	3.0	8.3	232	498	287	351	722
**Fruit and vegetable consumption**	1,555	4.1	1.1	8.3	305	334	342	301	273
**BMI**	−640	−1.7	−2.9	−0.4	−111	−129	−135	−133	−135
**Diabetes**	−2,820	−7.4	−12.6	−2.5	−365	−438	−533	−577	−908
**Total risk factors contribution**	12,990	34.1	21.1	47.3	2,107	2,690	2,591	2,704	2,896
**Total treatment contribution** d	19,780	52.0	40.2	69.7	3,670	4,055	4,420	4,166	3,471
**DPPs explained by model**	32,770	86.1	64.8	107.3	5,777	6,746	7,012	6,870	6,367
**DPPs not explained**	5,300	13.9			1,576	1,479	1,337	714	191
**Total DPPs**	38,070	100			7,353	8,225	8,349	7,584	6,558

Subtotals for England (column 1) have been rounded to nearest 5.

a The 95% uncertainty interval corresponds to the lower (2.5th percentile) and upper (97.5th percentile) limits of the uncertainty analysis.

b After subtracting DPPs due to hypertension treatment in primary prevention.

c After subtracting DPPs due to statin treatment in primary prevention.

d See Table 2 for detailed breakdown by patient group and treatment type.

Abbreviations: IMD, index of multiple deprivation.

**Table 4:** Percentage distribution of deaths prevented of postponed by deprivation quintile.

Treatments by Patient Groups; Risk Factors	England	Most Affluent	IMDQ2	IMDQ3	IMDQ4	Most Deprived
**Treatments**						
STEMI	**−0.3**	−0.1	−0.3	−0.3	−0.4	−0.6
NSTEACS	**0.8**	0.8	0.7	0.7	0.7	1.0
Secondary prevention post MI	**9.2**	8.7	9.4	8.9	9.4	9.7
Secondary prevention post revasc	**1.5**	1.5	1.5	1.5	1.5	1.7
Chronic stable CAD	**12.7**	11.6	12.2	12.2	13.3	14.6
Heart failure in the hospital	**0.7**	0.6	0.6	0.6	0.8	0.8
Heart failure in the community	**8.8**	7.7	8.4	8.8	9.4	9.8
Hypertension treatment	**4.7**	4.9	5.0	4.9	4.5	4.2
Hyperlipidemia treatment (statins)	**13.9**	14.3	11.9	15.6	15.7	11.8
**Total treatments** a	**52.0**	**49.9**	**49.3**	**52.9**	**54.9**	**52.9**
**Risk factors**						
Smoking	2.7	1.3	1.8	2.3	3.2	5.6
Diabetes	−7.4	−5.0	−5.3	−6.4	−7.6	−13.8
Physical inactivity	1.6	1.2	1.3	1.4	1.7	2.4
Systolic blood pressure, mmHg	29.3	25.3	26.4	27.8	31.5	36.9
Total cholesterol, mmol/l	5.5	3.2	6.1	3.4	4.6	11.0
BMI	−1.7	−1.5	−1.6	−1.6	−1.7	−2.1
Fruit and vegetable consumption	4.1	4.2	4.1	4.1	4.0	4.2
**Total Risk Factors** a	**34.1**	**28.7**	**32.7**	**31.0**	**35.7**	**44.2**
**DPPs explained by model** a	**86.1**	**78.6**	**82.0**	**84.0**	**90.6**	**97.1**
**DPPs not explained by model** a	**13.9**	**21.4**	**18.0**	**16.0**	**9.4**	**2.9**
**DPP Counts** a ^,^ b						
**DPPs explained by model** a **^,^** b	**32,770**	**5,777**	**6,746**	**7,012**	**6,870**	**6,367**
- Due to treatment uptake^b^	***19,780***	***3,670***	***4,055***	***4,420***	***4,166***	***3,471***
- Due to risk factor change^b^	***12,990***	***2,107***	***2,690***	***2,591***	***2,704***	***2,896***
**DPPs unexplained by model** a **^,^** b	**5,300**	**1,576**	**1,479**	**1,337**	**714**	**191**
**Total DPPs** a **^,^** b	**38,070**	**7,353**	**8,225**	**8,349**	**7,584**	**6,558**

a Sub-totals (in rows).

b DPP counts for England (column 1) have been rounded to nearest 5. All counts are in italics.

c Abbreviations: CAD, coronary artery disease; IMD, index of multiple deprivation; NSTEACS, non-ST elevation acute coronary syndrome; MI, myocardial infarction; revasc, revascularisation; STEMI, ST elevation myocardial infarction.

The most substantial contribution to deaths prevented by treatments came from statin treatment for hyperlipidemia (14% of the total mortality reduction, 5%–32%), management of chronic stable coronary artery disease (13%; 10%–17%), and secondary prevention following myocardial infarction or revascularisation (11%; 8%–13%) (Table 2). Uptake rates of statins and angiotensin-converting enzyme inhibitors (ACE inhibitors) or angiotensin receptor blockers (ARB) more than doubled for secondary prevention and the management of stable coronary artery disease (Table 5). These two therapies together contributed some 6,340 DPPs (17%). In contrast, deaths averted due to changes in treatment uptake in hospital-based patient groups were relatively modest: contributing just 165 fewer deaths (0.5%; −1% to 3%) amongst emergency admissions for infarction and unstable angina (ST elevation myocardial infarction [STEMI] and non-ST elevation acute coronary syndrome [NSTEACS], respectively). Improved heart failure treatments in the community resulted in approximately 3,335 fewer deaths (9%; 7%–10%), with relatively modest gains (250 fewer deaths) in hospitalised patients. Furthermore, there were essentially no gradients in treatment uptake across deprivation quintiles for either hospital treatment or drugs prescribed in the community for secondary prevention and heart failure (Table 5).

**Table 5:** Percentage treatment uptake rates 2000 and 2007 for England and stratified by deprivation quintiles.

Treatment by Patient Groups	Eligible Patients^a^	England		Most Affluent		IMDQ2		IMDQ3		IMDQ4		Most Deprived	
		2000^b^	2007^b^	2000	2007	2000	2007	2000	2007	2000	2007	2000	2007
**STEMI**	**20,700**												
Thrombolysis		**77.2**	**56.7**	79.4	58.6	77.9	59.5	75.4	57.1	76.2	56.0	77.4	52.5
Aspirin		**93.6**	**96.0**	93.6	96.6	94.7	96.3	93.1	95.4	93.2	95.6	93.4	96.4
B-blocker		**71.3**	**70.3**	74.8	70.9	72.3	69.1	71.0	69.8	69.6	69.5	69.9	72.4
ACE inhibitor or ARB		**77.2**	**76.3**	79.8	76.6	78.9	75.6	75.4	75.5	75.4	74.8	77.3	79.2
Clopidogrel		**27.7**	**88.5**	26.9	88.7	25.7	87.7	28.0	88.4	28.5	88.4	28.9	89.2
Primary PCI		**3.9**	**23.7**	2.9	24.2	3.4	21.8	3.8	23.3	4.3	24.5	4.8	24.8
Primary CABG		**0.1**	**0.1**	0.1	0.1	0.0	0.1	0.1	0.1	0.1	0.2	0.0	0.1
CPR in hospital		**11.4**	**6.6**	9.9	6.5	11.6	7.1	11.7	6.3	11.8	6.7	11.6	6.3
**NSTEACS**	**91,285**												
Aspirin and heparin		**64.0**	**79.7**	67.1	79.7	65.0	80.3	66.7	79.9	65.7	80.2	57.9	78.8
Aspirin alone		**24.2**	**12.8**	21.5	13.5	23.9	12.3	21.5	12.7	23.5	12.6	28.9	13.1
PG IIB/IIIA		**6.1**	**5.8**	**9.4**	**6.2**	**7.3**	**5.8**	**6.1**	**5.1**	**4.7**	**5.2**	**4.3**	**6.8**
**ACE inhibitor or ARB**		**66.0**	**73.2**	68.6	73.1	64.5	72.2	65.9	72.5	64.3	72.7	67.0	75.0
B-blocker		**63.2**	**67.6**	66.1	68.2	62.7	67.7	63.5	66.7	61.7	66.3	62.8	69.2
Clopidogrel		**44.3**	**86.6**	43.5	87.1	44.2	86.9	42.3	86.8	45.6	85.9	45.4	86.3
CABG surgery		**3.0**	**2.6**	3.5	3.4	3.2	2.7	3.2	2.6	2.9	2.3	2.5	2.1
PCI		**3.1**	**6.7**	3.6	7.7	3.4	6.9	3.2	7.0	2.9	6.4	2.5	5.7
CPR in hospital		**5.3**	**2.3**	4.6	2.3	4.9	2.2	5.3	2.1	5.6	2.5	5.8	2.5
**Secondary prevention post revascularisation**	**111,930**												
Aspirin		**64.3**	**76.5**	58.8	73.5	63.5	76.2	62.5	76.5	65.7	76.1	71.2	80.0
B-blocker		**30.7**	**55.7**	29.2	52.7	30.5	55.6	29.8	56.4	31.7	56.9	32.3	56.9
ACE inhibitor or ARB		**30.1**	**64.2**	30.8	63.9	29.2	63.0	29.5	63.9	30.9	63.4	30.5	67.1
Statin		**58.2**	**84.5**	61.7	85.1	58.2	84.6	56.3	83.9	56.3	84.8	58.7	84.2
Warfarin		**7.4**	**6.7**	7.9	7.2	7.3	6.6	6.7	6.5	7.1	7.1	7.9	6.1
Rehabilitation (post CABG)		**73.0**	**73.0**	73.0	73.0	73.0	73.0	73.0	73.0	73.0	73.0	73.0	73.0
Rehabilitation (post PTCA)		**10.0**	**20.0**	10.0	20.0	10.0	20.0	10.0	20.0	10.0	20.0	10.0	20.0
**Secondary prevention post MI**	**565,595**												
Aspirin		**59.7**	**74.4**	56.4	72.4	60.0	74.3	59.2	74.5	58.8	74.8	63.3	75.8
B-blocker		**32.6**	**53.4**	34.0	54.0	34.0	54.6	31.6	53.3	32.2	52.9	31.7	52.4
ACE inhibitor or ARB		**31.3**	**62.0**	32.3	62.6	32.5	62.3	31.0	61.6	30.6	61.2	30.5	62.5
Statin		**37.1**	**77.4**	39.8	77.9	39.5	77.8	35.9	76.6	34.7	76.6	36.2	78.1
Warfarin		**6.6**	**8.1**	7.7	8.3	6.7	8.9	6.5	7.9	6.2	7.7	6.2	7.6
Rehabilitation		**45.0**	**45.0**	45.0	45.0	45.0	45.0	45.0	45.0	45.0	45.0	45.0	45.0
**Chronic stable CAD**	**984,805**												
Aspirin in community		**42.9**	**62.4**	38.7	57.2	42.6	61.4	44.7	64.3	42.9	63.4	45.0	65.3
Statin in community		**23.9**	**66.2**	25.4	63.4	24.2	65.4	23.7	66.5	23.0	66.3	23.3	69.2
ACE inhibitor or ARB		**19.8**	**45.7**	19.9	45.1	19.0	45.5	20.5	45.8	20.1	45.5	19.7	46.5
CABG surgery		**8.7**	**9.6**	8.8	9.8	8.7	9.7	9.6	10.3	8.7	9.7	7.7	8.8
**Heart failure - hospital**	**24,625**												
ACE inhibitor		**53.2**	**59.1**	51.8	57.6	52.1	57.9	52.7	58.6	53.4	59.4	55.2	61.4
B-blocker		**25.4**	**28.2**	24.3	27.0	24.5	27.2	25.0	27.8	25.6	28.5	27.1	30.1
Spironolactone		**20.7**	**22.9**	19.8	22.0	20.0	22.3	20.4	22.7	20.8	23.1	21.8	24.3
Aspirin		**59.2**	**73.9**	56.6	71.9	59.8	73.3	58.6	74.1	58.1	75.3	62.2	74.4
**Heart failure - community**	**172,770**												
ACE inhibitor or ARB		**45.6**	**68.9**	48.2	70.2	44.5	69.3	43.4	67.8	45.9	69.2	46.6	68.4
B-blocker		**10.4**	**34.2**	10.7	35.1	11.2	34.6	10.8	34.9	9.4	34.2	10.1	32.4
Spironolactone		**3.9**	**14.5**	4.3	14.7	3.9	14.9	3.6	13.0	3.9	15.1	4.0	14.9
Aspirin		**38.1**	**50.4**	37.9	46.3	38.3	49.9	37.3	50.3	37.0	51.8	40.0	52.7
**Hypertension treatment**	**35,280,845**	**8.3**	**13.5**	8.3	14.0	8.2	13.8	8.6	13.9	8.2	13.0	8.3	12.7
**Hyperlipidemia treatment**	**35,280,845**	**1.1**	**9.0**	1.0	7.9	1.1	8.5	1.1	9.1	1.4	10.3	1.3	9.1

The overall treatment uptake rate is a weighted average over all age groups 25+ and both sexes.

a Eligible patient numbers rounded to nearest 5.

b Sub-totals (in rows) for CHD patient groups.

Abbreviations: B-blocker: beta-blocker; CABG: coronary artery bypass graft; CAD, coronary artery disease; CPR, cardiopulmonary resuscitation; IMD, index of multiple deprivation; NSTEACS, non-ST elevation acute coronary syndrome; MI, myocardial infarction; PCI, percutaneous coronary intervention; PG, platelet glycoprotein; PTCA, percutaneous transluminal coronary angioplasty; STEMI, ST elevation myocardial infarction.

Of the deaths prevented due to population-level risk factor changes, the largest contribution came from the fall in systolic blood pressure amongst those not on hypertensive medications (11,160 fewer deaths, or 29%; 18%–40%) (Table 3). On the other hand, gains from hypertensive medication were modest (approximately 1,800 fewer deaths [5%; 2%–10%]) (Table 2). Blood pressure falls were twice as high in women (5.4 mmHg versus 2.5 mmHg in men) but were of a similar magnitude across all deprivation quintiles (Table 6). Both in terms of absolute numbers and proportions, more deaths were prevented because of blood pressure falls in the most deprived quintile than in the most affluent (Tables 3 and 4).

**Table 6:** Absolute change in risk factor levels between 2000 and 2007 for England and stratified by deprivation quintiles and sex.

Risk Factors	Overall Levels		Absolute Change in Percentage Points, 2000–2007					
	2000	2007	England^c^	Most Affluent	IMDQ2	IMDQ3	IMDQ4	Most Deprived
**Smoking prevalence (%)**								
Male	***27.2***	***23.6***	**−3.7**	−2.6	−3.1	−3.6	−4.1	−4.8
Female	***23.4***	***19.9***	**−3.5**	−2.5	−3.0	−3.4	−4.0	−4.6
**Diabetes prevalence (%)**								
Male	***3.7***	***6.5***	**2.8**	2.4	2.7	2.8	2.6	3.6
Female	***2.9***	***4.8***	**1.9**	1.6	1.4	1.6	2.1	2.8
**Physical inactivity (%)**								
Male	***80.9***	***74.0***	**−6.9**	−6.9	−6.7	−6.8	−6.9	−7.2
Female	***82.4***	***78.1***	**−4.3**	−4.3	−4.2	−4.2	−4.2	−4.4
**Systolic blood pressure (mmHg)**								
Male	***133.1***	***130.6***	**−2.5**	−2.6	−2.6	−2.5	−2.5	−2.4
Female	***131.0***	***125.6***	**−5.4**	−5.3	−5.5	−5.5	−5.5	−5.5
**Total cholesterol (mmol/l)**								
Male	***5.6***	***5.4***	**−0.1**	−0.2	−0.2	−0.2	−0.1	−0.1
Female	***5.7***	***5.5***	**−0.2**	−0.2	−0.2	−0.2	−0.2	−0.2
**BMI (kg/m^2^)**								
Male	***27.3***	***27.7***	**0.4**	0.4	0.4	0.4	0.3	0.3
Female	***26.9***	***27.2***	**0.2**	0.2	0.2	0.2	0.2	0.3
**Fruit and vegetable consumption (portions per day)**								
Male	***3.4***	***3.7***	**0.4**	0.4	0.4	0.4	0.4	0.3
Female	***3.6***	***4.0***	**0.4**	0.4	0.4	0.4	0.4	0.4

See Text S1, Table K for weighted averages of risk factor levels for each deprivation quintile, 2000 and 2007.

a England average weighted by 2007 population distribution in 10-y age bands.

IMD, index of multiple deprivation.

In contrast, the benefits attributable to statin lowering of total cholesterol levels were double those attributable to the fall in cholesterol levels in the population not on treatment (approximately 5,300 versus 2,090 fewer deaths, respectively). Between 2000 and 2007, hyperlipidemia treatment increased nine-fold across all social groups from 1% to 9% (Table 5). Total cholesterol levels fell marginally more in women than men and by a similar magnitude across deprivation quintiles (Table 6). Thus, while the proportionate fall in deaths attributable to cholesterol reduction in the general population was similar across quintiles, in absolute terms more deaths were prevented in the most deprived quintiles (Table 3).

Favourable trends in smoking, fruit and vegetable consumption, and physical activity were modest; together only contributing about 3,195 fewer deaths, or 8% (2%–18%) of the overall mortality fall (Table 2). Smoking prevalence declined in men and women by a similar amount (4%); however, there was a clear social gradient with larger absolute falls in smoking prevalence in more deprived quintiles (Table 6). Furthermore, levels of smoking still remained twice as high in the most deprived compared to affluent groups (Text S1, Table K). Physical inactivity fell more in men (7%) than women (4%) across all deprivation quintiles; however, three in four adults remained classed as inactive in every quintile.

Mortality gains due to positive trends in smoking, fruit and vegetable consumption, and physical activity risk factors were negated by increases in BMI and diabetes (together contributing 3,460 additional deaths, equivalent to an 9% increase in mortality (−17% to −3%) (Table 3). Even over the relatively short period of this analysis, the social gradient in diabetes became more pronounced resulting in three times as many additional diabetes-related deaths in the most deprived quintile compared with the most affluent.

### Model Fit

The percentage unexplained by the model varied by age, sex, and SECs. The model fit was generally good overall and in women and men living in the most deprived areas (Text S1, Table L.3). However, the fit was least good in affluent quintiles, with the 95% uncertainty limits around the DPPs explained by the model (5,777; 4,134–7,420) just overlapping with the number of observed DPPs (7,353). Model fit was also better for women than men (see Text S1, Figure L.1).

## Discussion

Between 2000 and 2007, English CHD mortality rates fell by an impressive 36%, resulting in approximately 38,000 fewer CHD deaths in 2007. However the relative mortality inequalities between rich and poor persisted and even increased slightly over this period. This study is the first, to our knowledge, to analyse the socioeconomic components concealed within the overall mortality reductions attributable specifically to risk factor trends and to evidence-based treatments. By using deprivation scores for area of residence as a unified marker of SECs across all relevant large databases of population health and health service use in England, the study had adequate statistical size to quantify the impact of changes in risk factors and treatments within socioeconomic groups, even over a relatively short period of 7 y. Understanding these recent trends, and their socially divergent trajectories, will be crucial to planning the most effective and equitable future strategies to prevent cardiovascular disease and reduce inequalities.

### Main Findings

Approximately half the fall in CHD mortality was attributable to increased medical therapies. These benefits largely reflected a doubling of drug use for community patients with chronic disease (who represent the largest CHD burden). In contrast, the contribution of medical interventions in hospital was relatively modest. Firstly, because the numbers of patients admitted to hospital with acute disease were much smaller. Secondly, because few new treatments were introduced after 2000 other than clopidogrel and primary angioplasty. And thirdly, the uptake rates for existing hospital-based treatments were already close to maximum levels in 2000. The age-specific prevalence of CHD is socially graded. With similar levels of uptake of treatments across socioeconomic quintiles in both base and final years, this meant that the benefits of increased treatment were distributed remarkably evenly across social groups, which suggests a fairly equitable distribution of therapies across the NHS.

Reductions in major cardiovascular risk factors explained over two-fifths of the fall in CHD mortality (43%). However, the net benefit was much smaller (approximately 34%) because adverse trends in BMI and diabetes potentially increased mortality by some 9%.

The single largest contribution to the overall CHD mortality decrease came from population falls in blood pressure [17] with relatively small gains from hypertension therapies. Furthermore, reductions were similar across social groups. This is therefore entirely consistent with recent UK population-wide reductions in salt intake [18], and with recent encouraging trends in other wealthy counties [19].

Small increases in fruit and vegetable consumption and physical activity were seen across all social groups. Furthermore, moderate declines in smoking levels were actually greater in deprived areas. This may reflect the benefit of cumulative tobacco control policies since 2000, reinforced by the targeting of cessation services in deprived areas [20].

However, after excluding the effect of statin therapy, the decline in cholesterol levels in the wider population was modest. This finding may well reflect a failure to implement more effective dietary policies [21]. Particularly worrying was the approximately 3,500 additional deaths attributable to the continuing rises in diabetes and BMI. This number is consistent with recent Foresight analyses and represents a further warning to policy makers [22].

The absolute gap in CHD mortality between the most affluent and most deprived groups narrowed over the period of our study, however relative inequalities widened. This was unlikely to be due to differential treatment of diagnosed patients because levels of uptake of evidence-based therapies were similar for all groups. The pace of fall in mortality in the most affluent groups was faster; but changes in risk factor levels could not explain about 20% of this fall. By contrast, in the most deprived quintile, changes in risk factor levels explained almost all of the remaining CHD mortality fall after accounting for deaths averted due to increase in treatment uptake rates. Perhaps the most likely explanation for this difference is a social gradient in effect modification. Thus, the current model assumed that the mortality decrease per unit change in risk factor was similar across deprivation quintiles. However, the benefits of a specific decrease in blood pressure or cholesterol may be disproportionately higher in more affluent groups, perhaps reflecting synergy with other positive trends [23]. A recent cohort analysis found that even if four classic risk factors—blood pressure, cholesterol, smoking, and diabetes—were to be completely eliminated in middle-aged men, relative inequalities in CHD mortality between those in low and high employment grades would persist despite a 70% reduction in absolute mortality differences [24]. Furthermore we do not have many estimates of the cumulative benefit associated with a lifetime of low risk. For example, the Finnish Public Sector Study found that a marked socioeconomic gradient in absolute risk of CHD mortality persisted even in a low-risk subgroup that had never smoked, were not obese or physically inactive, and who consumed moderate amounts of alcohol [25].

Alternative explanations for the fraction of the mortality fall unexplained by the model include the possible omission of more “upstream” risks such as psychosocial stress, which might differentially benefit affluent groups [23],[26]. Differential levels of adherence to prescribed medications may also play a role; however, this is a relatively under-researched area without clear-cut evidence to support or refute the existence of systematic social gradients [27]–[29]. We also tested the impact of varying adherence rates differentially on the DPPs explained. However, varying adherence rates in this way had a limited effect on reducing the gradient in the proportion unexplained. Finally, measurement error may contribute; Health Survey estimates of risk factor trends by deprivation quintiles may lack precision because of small samples and differential response rates.

### Comparisons with Other UK and International Studies

Compared with previous IMPACT analyses from a baseline of the 1980s [4],[5],[30]–[33], models of more recent changes, for example in Ontario [6], demonstrate the growing relative contribution from improved treatments to reductions in CHD mortality. However, country-specific proportions attributable to risk factors or treatments are relative to the scale of the decline, and hence potentially misleading. Thus, although Nordic countries possess uniformly good health services, their larger absolute falls in coronary mortality mainly reflect particularly impressive decreases in major risk factors, mainly cholesterol, blood pressure and smoking, and smaller increases in obesity and diabetes [33],[34].

### Strengths and Limitations of This Study

The IMPACT model has been replicated and validated in diverse national populations. This is the first IMPACT study to quantify the socioeconomic components of the contributions of changes in treatment and risk factors to falls in coronary mortality. The main datasets used are reasonably representative of the socioeconomic distribution of the English population and large enough for reasonably accurate estimates of socioeconomic change.

However, a number of limitations should also be acknowledged. These include the use of area-level categorisation of SECs. However, area deprivation correlates well with individual socioeconomic position and may also help to capture the contextual effects of living conditions [35],[36].

Approximately 14% of the CHD mortality fall was not explained by the model and the uncertainty analysis also produced wide limits in the percentage explained (86%; 65%–107%). The model fit was also less good in men in affluent areas, as discussed earlier. However, the model fit was generally good overall and in women and in men living in the most deprived areas. As with all models attempting to capture complex and interacting changes, it remains possible that there were additional (unquantifiable) sources of error not captured by the uncertainty analysis.

### Conclusions and Implications for Future Prevention Policies and Strategies

Approximately half of the recent substantial CHD mortality fall in England was attributable to medical therapies. Benefits were relatively even across social groups. These findings are consistent with equitable service delivery across the NHS. Treatment uptake in hospitals was close to maximum levels over the entire period, while follow-up treatment of cardiovascular patients in the community substantially improved and was equitable. This suggests the Qualities and Outcome Framework that was being implemented in general practice during the study period was an effective incentive for improving uptake overall [37].

However, the net gains from risk factor improvements were small, reflecting modest recent decreases in powerful cardiovascular risk factors such as smoking and cholesterol, and further eroded by continuing rises in BMI and diabetes. This throws a spotlight on recent UK policies for salt reduction and tobacco control (relatively effective) and healthier diets (relatively neglected). Elsewhere, the successful introduction of effective, powerful, rapid, and cost-saving policy interventions have achieved substantial reductions in the saturated fat, trans-fats, sugars, and calories hidden in processed food, takeaways, and sweetened drinks [21],[37],[38]. Mandatory interventions involving legislation, regulation, taxation, or subsidies consistently appear more effective and cost saving than voluntary schemes [19],[39],[40]. They also tend to be equitable [12] and surprisingly rapid [15],[38]. The UK now has an equally pressing need for population-wide policy interventions to effectively tackle persistent inequalities in cardiovascular mortality.

## Supporting Information

Text S1 Technical appendix for the IMPACT_SEC_ model. Contents are as follows. Section 1: Overview of the IMPACT_SEC_ model. Table A: Population and patient data sources. Table B: Data sources for treatment uptake levels: medical and surgical treatments included in the model. Table C: Risk factors: variable definitions and source. Table D: Cumulative benefit: adjustment factors by age, sex, and IMD quintile. Table E: CHD mortality rates in 2000 and 2007 by sex and deprivation quintiles. Table F: Clinical efficacy of interventions: relative risk reductions obtained from meta-analyses, and randomised clinical trials. Table G: Case fatality rates for each patient group. Table H: Treatment uptake in 2000 and 2007. Table I: Beta coefficients for major risk factors. Table J: Relative risk values for CHD mortality: smoking, diabetes, and physical inactivity. Table K: Risk factor levels in 2000 and 2007 by sex and deprivation quintiles. Table L: Model fit by age, sex, and deprivation quintiles. Table M: Uncertainty analysis: parameter distributions, functions, and sources. Table N: Assumptions and overlap adjustments used in the IMPACT_SEC_ Model. Table O: “Fixed gradients” for measuring risk factor change between two time points for deprivation quintiles.
(DOC)

## Abbreviations

ARB: angiotensin receptor blocker
ACE inhibitor: angiotensin-converting enzyme inhibitors
BMI: body mass index
CHD: coronary heart disease
DPP: deaths prevented or postponed
NHS: National Health Service
SEC: socioeconomic circumstance
