# Supplementary material for: Analysing Recent Socioeconomic Trends in Coronary Heart Disease Mortality in England, 2000–2007: A Population Modelling Study
Source: PLoS Med. 2012 Jun 12;9(6):e1001237. doi: 10.1371/journal.pmed.1001237 (PMC3373639; doi:10.1371/journal.pmed.1001237)
Supplement: Text S1 — Technical appendix for the IMPACTSEC model. Contents are as follows. Section 1: Overview of the IMPACTSEC model. Table A: Population and patient data sources. Table B: Data sources for treatment uptake levels: medical and surgical treatments included in the model. Table C: Risk factors: variable definitions and source. Table D: Cumulative benefit: adjustment factors by age, sex, and IMD quintile. Table E: CHD mortality rates in 2000 and 2007 by sex and deprivation quintiles. Table F: Clinical efficacy of interventions: relative risk reductions obtained from meta-analyses, and randomised clinical trials. Table G: Case fatality rates for each patient group. Table H: Treatment uptake in 2000 and 2007. Table I: Beta coefficients for major risk factors. Table J: Relative risk values for CHD mortality: smoking, diabetes, and physical inactivity. Table K: Risk factor levels in 2000 and 2007 by sex and deprivation quintiles. Table L: Model fit by age, sex, and deprivation quintiles. Table M: Uncertainty analysis: parameter distributions, functions, and sources. Table N: Assumptions and overlap adjustments used in the IMPACTSEC Model. Table O: “Fixed gradients” for measuring risk factor change between two time points for deprivation quintiles. (DOC) [file pmed.1001237.s001.doc]

**Text S1: TECHNICAL APPENDIX FOR THE IMPACT_SEC_ MODEL**

***April 2012***

**Authors: Shaun Scholes, Madhavi Bajekal**, Hande Love, Nathaniel Hawkins, Martin O’Flaherty, Rosalind Raine & Simon Capewell

| **Contents** |  | Page |
| --- | --- | --- |
|  | ***List of abbreviations*** | 2 |
|  |  |  |
| ***1*** | **Overview of the IMPACT_SEC_ model** |  |
| *1.1* | Introduction | 3 |
| *1.2* | Method and examples of deaths prevented or postponed (DPP) calculations | 4 |
| *1.3* | Cumulative risk reduction | 9 |
| *1.4* | Other methodological considerations | 13 |
|  |  |  |
| ***Table A.*** | **Population and patient data sources** | 18 |
| ***Table B.*** | **Data sources for treatment uptake levels:** Medical and surgical treatments included in the model | 20 |
| ***Table C.*** | **Risk factors – variable definitions and source** | 22 |
| ***Table D.*** | **Cumulative benefit: Adjustment factors by age, sex and IMD quintile** | 24 |
| ***Table E.*** | **CHD mortality rates in 2000 and 2007 by sex and deprivation quintiles** | 25 |
| ***Table F.*** | **Clinical efficacy of interventions:** Relative risk reductions obtained from meta-analyses, and randomised clinical trials | 26 |
| ***Table G.*** | **Case fatality rates for each patient group** | 31 |
| ***Table H.*** | **Treatment uptake in 2000 and 2007** | 32 |
| ***Table I.*** | **Beta coefficients for major risk factors** | 34 |
| ***Table J.*** | **Relative risk values for CHD mortality: smoking, diabetes and physical inactivity** | 36 |
| ***Table K.*** | **Risk factor levels in 2000 and 2007 by sex and deprivation quintiles** | 44 |
| ***Table L.*** | **Model fit by age, sex and deprivation quintiles** | 45 |
| ***Table M.*** | **Uncertainty analysis: parameter distributions, functions and sources** | 49 |
| ***Table N*.** | **Assumptions and overlap adjustments used in the IMPACT_SEC_ Model** | 65 |
| ***Table O.*** | **‘Fixed gradients’ for measuring risk factor change between two time points for deprivation quintiles** | 69 |
|  |  |  |
|  | ***Reference list*** | 72 |

| **List of abbreviations:** | |
| --- | --- |
| ACE Inhibitors | Angiotensin-converting enzyme inhibitors |
| AMI/MI  AR | Acute myocardial infarction  Additive risk-reduction |
| ARB | Angiotensin receptor blocker |
| BMI | Body mass index |
| CABG | Coronary artery bypass graft |
| CAD | Coronary artery disease |
| CFR | Case fatality rates |
| CHD | Coronary heart disease |
| CPR | Cardiopulmonary resuscitation |
| CR | Combined/cumulative risk-reduction |
| DPP  GBD | Deaths prevented or postponed  Global burden of disease |
| GPRD | General practice research database |
| HES | Hospital episode statistics |
| HF | Heart failure |
| HSfE | Health survey for England |
| ICD | International classification of diseases |
| IMD | Index of multiple deprivation |
| MINAP | Myocardial ischaemia national audit project |
| NHANES | National health and nutrition examination survey |
| NHS | National health service |
| NSTEACS | Non-ST elevation acute coronary syndrome |
| NSTEMI | Non-ST elevation myocardial infarction |
| ONS | Office for National Statistics |
| OPCS codes | Office of Population Censuses and Surveys classification of surgical operations and procedures (used in HES) |
| PARF | Population attributable risk fraction |
| PCI | Percutaneous coronary intervention |
| PG IIB/IIIA | Platelet glycoprotein IIB/IIIA inhibitors |
| PTCA | Percutaneous coronary intervention |
| RRR | Relative risk reduction |
| SBP | Systolic blood pressure |
| SE | Standard error |
| SEC | Socio-economic circumstances |
| STEMI | ST-elevation myocardial infarction |
| UA  WHO | Unstable angina  World Health Organization |

# 1 Overview of the IMPACT_SEC_ model

**1.1 INTRODUCTION**

IMPACT is a deterministic, cell-based policy model. It uses epidemiological information to estimate the contributions of population-level risk factor changes (impacting mainly on incidence) and changes in the uptake of evidence-based treatments (impacting mainly on case fatality) on mortality decline between two points in time (the start-year and the end-year). The primary outcome measure of the model is the deaths prevented or postponed (DPPs).

The starting point for the model is to calculate the ‘target’ number of deaths the model needs to explain. This target number is obtained by using death counts recorded in the official registration system to calculate the difference between the actual observed Coronary Heart Disease (CHD) deaths recorded in the end-year and the deaths expected in the end-year had the CHD mortality rates remained the same as in the start-year (i.e. simple direct standardisation).

The calculation of the modelled estimate of DPPs rests on utilising two well-studied relationships: firstly, that between risk factor change and the relative reduction in CHD mortality; secondly, that between treatment uptake and reductions in case-fatality in patients with a specific form of CHD.

The model applies the relative risk reduction quantified in previous randomised controlled trials and meta-analyses to estimate the mortality reduction attributable to:

a) temporal change in risk factor prevalence (in those without diagnosed CHD) to calculate the DPPs ‘explained’ by specific risk factor trends;

b) net change over the period in the uptake of specific treatments in patients with each specific form of CHD to estimate DPPs ‘explained’ owing to improved 1-year case fatality rates. Great care is taken to avoid double counting the same individuals.

The mortality benefits from the risk factor reduction in the population, and the treatment benefits in patient groups are then summed. Thus summing uses a cumulative approach (rather than an additive approach), in order to avoid double-counting of benefits in the same individual. (This approach is detailed in Section 1.3).

This mortality sum represents the deaths prevented or postponed (DPPs) ‘explained’ by the model.

At the end of the modelling process, the total DPPs ‘explained’ by the model is then compared with the observed fall in deaths (the ‘target’ to be explained).

**Model fit** is therefore calculated as the difference between the observed deaths and model DPPs, and expressed as the percentage explained. This measures the extent to which the model was successful in explaining the observed change in CHD mortality in the population.

A policy model like IMPACT thus stands in contrast to a typical multivariate regression model. A typical multivariate regression model represents a statistical approach to describing a single data-set, for instance generated by a single cohort or randomised controlled trial. In contrast, a policy model such as IMPACT seeks to integrate and synthesise best estimates from a variety of sources to reliably estimate the extent to which a range of factors, acting in combination, explain or predict an outcome. We did not obtain the parameters for this model by running regressions. Rather, the model incorporates the best coefficients from the largest meta-analysis or randomised controlled trials of the reduction in case fatality attributed to treatment or the independent effect sizes of a unit change in each risk factor on CHD mortality.

Examples of the calculation method used for estimating the DPPs due to treatment uptake (Example 1) and for continuous and binary risk factor change (Examples 2 and 3, respectively) are provided below. Earlier versions of the IMPACT mortality model have been previously applied to national data from Europe, United States, Ontario, New Zealand and China [1-5]. The methodology has previously been described in detail online and elsewhere [1-2,4].

***The IMPACT_SEC_ model***

We have now extended the IMPACT model to accommodate sub-national variation in CHD mortality trends by socioeconomic circumstances (IMPACT_SEC_ model). We used the Index of Multiple Deprivation 2007 (IMD) quintiles as a proxy indicator of socioeconomic circumstances. This model examines the effects of changes in treatment uptake and risk factor trends on changes in mortality from coronary heart disease (CHD) among adults in England aged 25 years and over, stratified into equal quintiles by population size. The tables included in this Technical Appendix provide details about the sources and methods that were used.

**1.2. METHOD AND EXAMPLES OF DEATHS PREVENTED OR POSTPONED (DPP) CALCULATIONS**

**1.2.1** Changes in mortality rates from CHD, England 2000 to 2007

Data sources used in examining the changes in CHD mortality rates over 2000 to 2007 are shown in Table A. Mortality rates from CHD were calculated using the underlying cause of death (*2000*: ICD9 410-414; *2007*: ICD10 I20-I25). Both unadjusted and age-adjusted mortality rates were calculated. The direct method of age-standardisation was used with the European Union reference population as standard.

**1.2.2** Expected and observed number of deaths from CHD

Data sources used to estimate the observed and expected number of deaths from CHD for 2000 and 2007 are shown in Table A. The expected number of CHD deaths in 2007 was calculated by multiplying the age-sex-IMD quintile specific mortality rates from CHD in 2000 by the population counts for 2007 in that age-sex-IMD quintile stratum. Summing over all strata then yielded the expected number of deaths in 2007 had mortality rates remained unchanged. The difference between the number of expected and observed deaths from CHD represented the mortality fall, or the total DPPs in 2007 relative to 2000. Population counts, CHD mortality rates, observed and expected numbers of deaths are shown in Table E.

**1.2.3** Treatment component of IMPACT_SEC_ model

The treatment component of the IMPACT_SEC_ model included nine mutually exclusive CHD patient groups:

- Patients treated in hospital for acute myocardial infarction (ST-elevation myocardial infarction and non-ST elevation acute coronary syndrome)
- Patients admitted to hospital with unstable angina
- Community-dwelling patients who have survived a myocardial infarction for over a year
- Patients who have undergone a revascularisation procedure up to and including the years 2000 and 2007: Coronary Artery Bypass Grafting (CABG), or a Percutaneous Coronary Intervention (PCI)
- Community-dwelling patients with stable coronary artery disease
- Patients admitted to hospital with heart failure (due to CHD)
- Community-dwelling patients with heart failure (due to CHD)
- Hypercholesterolaemic subjects without CHD eligible for cholesterol lowering therapy such as statins
- Hypertensive individuals without CHD eligible for anti-hypertensive therapy

ST-segment and non-ST segment elevation myocardial infarction (STEMI and non-STEMI respectively) patients were examined separately as the management and outcomes of these entities differ markedly [6].

In order to minimise double counting, major efforts were made to ensure that patients counted in each CHD patient group were mutually exclusive. These approaches are detailed later, in Table N.

The data sources used to estimate the size of each treatment group (stratified by age-sex-IMD quintile) are shown in Table A. For each group, we estimated the number of DPPs that were attributable to various treatments. A list of the treatments considered in the model and the data sources used to estimate the percentage of patients receiving treatments in the start and end year is shown in Table B.

The general approach to calculating the number of DPPs from an intervention among a particular patient group was first to stratify by age, sex and IMD quintile; then to multiply the estimated number of patients in 2007 in turn by: the proportion of these patients receiving a particular treatment; the one-year case fatality rate; and the relative reduction in the case fatality rate due to the administered treatment. Sources for treatment uptake are shown in Table B. Sources for estimates of treatment efficacy (relative risk reductions) are shown in Table F. We obtained the relative risks based on the most recent published systematic reviews and meta-analyses of epidemiological studies. Each treatment relative risk value in the model was based on a meta-analysis comparison with an older therapy, or in some cases with a placebo if relevant. Age-sex specific case fatality rates for each patient group are presented in Table G.

It was assumed that compliance (adherence), i.e. the proportion of treated patients actually taking therapeutically effective levels of medication, was 100% among hospital patients, 70% among symptomatic community patients, and 50% among asymptomatic community patients taking lipid-lowering drugs or anti-hypertensive medication for primary prevention. An adjustment was also made in certain cases for sub-optimal dose.

**Example 1: Estimation of DPPs from a specific treatment**

*Mortality fall in STEMI patients as a result of taking aspirin in men aged 55-64 in the most affluent quintile*

For example, in 2007, about 1,410 men aged 55-64 in the most affluent quintile were hospitalised with myocardial infarction (ICD10: I21). 40% of these were assumed to be STEMI cases. Uptake of aspirin was estimated to be approximately 99% with 100% assumed to comply. Aspirin use reduces case fatality in patients with ST-segment elevation by approximately 23%. The underlying one-year case fatality rate in these men was approximately 6%. The DPPs for at least a year were therefore calculated as:

***Patient numbers × treatment uptake × compliance × relative mortality reduction × one year case fatality***

= (1,410 × 40%) × 99% × 100% × 23% × 6% ≈ 8 DPPs

This calculation was then repeated:

1. For each age-sex-IMD quintile group (70 in total).
2. Incorporating a Mant and Hicks adjustment for multiple medications within each patient group (see Section 1.4.1).

**1.2.4** Risk factor component of IMPACT_SEC_ model

The second part of the IMPACT_SEC_ model estimated the number of DPPs related to changes in cardiovascular risk factor levels in the population. The risk factors considered were cigarette smoking, total cholesterol, systolic blood pressure, body mass index, diabetes, physical activity, and fruit and vegetable consumption. The Health Survey for England was used to calculate trends in the prevalence (or mean values) of each risk factor (Table C). Two approaches to calculating DPPs from changes in risk factors were used: the regression approach and change in the Population Attributable Risk Fraction (PARF). These are illustrated below.

*Estimating DPPs from risk factor change – regression approach for continuous risk factors*

In the regression approach – used for systolic blood pressure (SBP), total cholesterol, body mass index, and fruit and vegetable consumption – the number of CHD deaths in 2000 (the start year) after adjusting for population change between 2000 and 2007 were multiplied by the absolute change in risk factor level, and by a regression coefficient (‘beta’) quantifying the estimated relative change in CHD mortality that would result from a one-unit change in risk factor level (Table I). Natural logarithms were used, as is conventional, in order to best describe the log-linear relationship between absolute changes in risk factor levels and relative change in mortality. For example, using the meta-analysis by Dauchet et.al [7], a beta coefficient of -0.04 corresponds to an estimated 4% reduction in CHD mortality rates for a one-unit increase in the mean number of fruit and vegetable portions consumed per day. Levels of risk factors in 2000 and 2007 by sex and IMD quintile are shown in Table K.

**Example 2: Estimation of DPPs from risk factor changes using regression method**

*Mortality fall due to reduction in SBP in women aged 55-64 in the most affluent quintile*

For example, in 2000, there were 227 CHD deaths among 573,291 women aged 55-64 years in the most affluent quintile. The population total had increased to 714,111 in 2007. Applying the CHD death rate from 2000 (39.6 per 100,000) to the 2007 population gives an (adjusted) total of 283 expected deaths in 2007.

Mean SBP in this group fell by an estimated 4.28 millimetres of mercury (mmHg) (from 133.8 in 2000 to 129.5 in 2007). The largest meta-analysis reports an estimated age-sex specific reduction in mortality of 50% for every 20 mmHg reduction in SBP, generating a logarithmic coefficient of -0.035 (i.e. natural logarithm of 0.5 divided by 20). The subsequent reduction in CHD deaths between 2000 and 2007 was then estimated as the product of three variables:

***DPPs = expected CHD deaths in 2007 (had mortality rates in 2000 remained constant) × absolute risk factor reduction between 2000 and 2007 × regression coefficient exponentiated***

DPPs = (1-(exponential (regression coefficient × absolute change))) × expected deaths in 2007

DPPs = (1-(exponential (-0.035 × 4.28))) × 283 ≈ 39

This calculation was then repeated for each age-sex-quintile group.

Data sources for the number of expected CHD deaths are shown in Table A, the Health Survey for England (HSfE) was used to estimate risk factor trends (Table C), and sources for the regression (beta) coefficients used in these analyses are listed in Table I. The regression coefficients were assumed equal across deprivation quintiles. A ‘fixed gradient’ approach was used to stabilise estimates of risk factor change across the quintiles; this method is discussed in Table O.

*Estimating DPPs from risk factor change – PARF approach for binary risk factors*

The PARF approach was used for cigarette smoking, diabetes, and physical activity. PARF, which can be interpreted as the proportion by which the mortality rate from CHD would be reduced if the exposure were eliminated [8], was calculated as:

***PARF = [P × (RR - 1)] / [1 + P × (RR - 1)]***

Where P is the prevalence of the risk factor and RR is the relative risk for CHD mortality associated with risk factor presence. A relative risk of 1.69 associated with smoking, for example, expresses the ratio of risk of CHD mortality in smokers to that in non-smokers. DPPs were then estimated as the expected CHD deaths in 2007 (had mortality rates in 2000 remained constant) multiplied by the difference in PARF for 2000 and 2007.

**Example 3: Estimation of DPPs from risk factor changes using the PARF method**

*Mortality increase due to increase in diabetes in men aged 65-74 in the most deprived quintile*

For example, the prevalence of diabetes among men aged 65-74 years was 7% in 1998 and 15.7% in 2006. Assuming a relative risk of 1.86, the PARF at the national level for men aged 65-74 was 0.057 in 1998 and 0.119 in 2006.

Using estimates of diabetes prevalence pooled over 1998, 2003, and 2006 survey data (to maximise precision), and the same relative risk value of 1.86, a ‘risk factor’ gradient was calculated using the ratio of the PARF at the national level to that in each deprivation quintile (See Table O for details of the SEC gradient approach). The risk factor gradient in the PARF in the most deprived quintile was estimated to be 1.38 times higher than the national. The gradient of 1.38 was then applied to the national PARF values in the base and final year of the model (0.057 and 0.119 respectively) to give estimated PARFs of 0.079 (start year) and 0.164 (end year) for men aged 65-74 in the most deprived quintile. The DPPs attributable to the increase in diabetes prevalence was therefore:

***DPPs = expected CHD deaths in 2007 (had mortality rates in 2000 remained constant) × (PARF_2000_ – PARF_2007_)***

DPPs = expected CHD deaths in 2007 (3,583) × (0.079 – 0.164) ≈ -305 DPPs

A negative sign for the DPPs denotes deaths increased or brought-forward due to the increase in diabetes prevalence. The calculation was then repeated for each age-sex-quintile group.

Relative risks estimated by expert working groups for the World Health Organization’s Global Burden of Disease 2001 Study were used for smoking and physical activity [9]. Effect estimates were based on systematic reviews of cohort studies (adjusted for regression dilution bias) and meta-analyses of randomised controlled trials. Age-variation in the relative risks for diabetes were taken from the DECODE study [10]. These were then applied to the sex-variation in relative risks estimated by Huxley et al [11]. The published relative risk values for smoking, physical activity and diabetes are shown in Table J. These were adjusted in our study to: a) match the 10-year age bands used in IMPACT_SEC_ and b) employ a dichotomous rather than trichotomous measure of physical activity. Full details on these adjustments are given in Table J.

**1.3** CUMULATIVE RISK-REDUCTION: ADJUSTING DEATHS PREVENTED OR POSTPONED (DPPs) TO CALCULATE CUMULATIVE BENEFIT OF MULTIPLE RISK FACTOR CHANGES

**1.3.1** Background

CHD deaths are usually caused by multiple risk factors acting simultaneously. Hence, part of the effect of one risk factor may be mediated through another. For example, physical inactivity may have a direct effect on CHD but may also partly be mediated through its effects on BMI and blood pressure. It is recommended therefore that mortality benefits attributable to risk factors which may be causally related, or which overlap in population groups, should not be combined by simple addition. Ideally, their effects should instead be jointly estimated [12-16].

We do not currently have sources that allow joint estimation of relative risks for combinations of risk factors in this English population. However, several large cohort studies and meta-analyses have published independent risk reduction coefficients for each risk factor included in this study. These are detailed in Tables I and J for continuous and dichotomous risk factors, respectively. One approach commonly used is to calculate the **cumulative risk-**reduction [17]. This approach accounts for risk factor prevalence overlap but assumes independence of effects [14-15]. The general equation for cumulative risk-reduction is stated as:

Combined (or cumulative) effect (CR) =

1 – ((1-a) × (1-b) × (1-c) ×….× (1-n)) **[1]**

Thus for CHD risk factors, the specific equation is stated as:

**CR** = 1 – ((1-R_SBP_) × (1-R_smoke_) × (1-R_diabetes_) ×….× (1-R_n_))

where R denotes the mortality change attributable to a specific risk factor.

This is in contrast to additive risk-reduction (AR):

**AR** = (R_SBP_) + (R_smoke_) + (R_diabetes_) +…..+ (R_n_) **[2]**

**1.3.2 Implementation**

For the purposes of this modelling study we first calculated the (additive) DPPs attributed to risk factor change. These were then adjusted down by using the ratio:

**Adjustment factor** = CR/AR

The adjustment factor would always be expected to be less than 1. In other words, cumulative risk factor reduction would be smaller than the mortality benefits arrived at by a simple summation of the benefits of each risk factor in turn.

The proportional change in the CHD mortality rate between two time points (denoted by R) was calculated using the following formulas [14-15]:

Continuous risk factors:

R_continuous_  = 1 – exp(beta × absolute mean risk factor change) **[3]**

Dichotomous risk factors:

R_dichotomous_  = PARF × (ΔP/P) **[4]**

where PARF = [P × (RR - 1)] / [1 + P × (RR - 1)]

and P denotes prevalence at the start-year; RR the relative risk in CHD mortality associated with risk factor presence; and ΔP the change in prevalence between the start and final years.

Formulas [3] and [4] were used to calculate the proportional change in the CHD mortality rate (R) for each risk factor and the steps involved in their estimation are detailed below. However, we made two modifications to the methodology used in previous work [14-15]. First, we estimated aggregate change over a seven year period (2000-2007) rather than average annual change. Second, additive and cumulative risk-reduction was calculated by using the **absolute** values of R (i.e. disregarding the direction of risk factor change). These are discussed in turn below.

*Calculating aggregate change in risk factors over 2000 and 2007*

Previous studies [14-15] estimating cumulative risk factor reduction calculated the average annual percentage change in CHD mortality attributable to annual falls in levels of smoking, blood pressure and cholesterol (where annual falls in CHD mortality and risk factor levels were estimated over a specified number of years). Rather than estimate the average annual change over a specific range of years, we were interested in calculating the R values between two fixed points in time (start and end years of the model), seven years apart, 2000 and 2007. We therefore adapted formulas [3] and [4], substituting change over the seven year study period for the estimation of annual average change. We checked our resulting estimates of cumulative risk reduction calculated over seven years against uprating the annual average by a factor of seven. The two sets of estimates were found to be virtually identical.

*Regression models to estimate risk factor change, 2000-2007*

Formulas [3] and [4] require estimates of absolute and relative change in risk factors, respectively. Regression modelling was used to estimate the magnitude of absolute and relative change. In order to smooth fluctuations in Health Survey for England data, we obtained estimates of risk factor change for each risk factor over 2000-2007 by using the predicted values from regression models. Separate models were fitted by sex and seven ten-year age-bands (14 in total for each risk factor).

Estimates of absolute change in the mean levels of risk factors measured on a continuous scale (blood pressure, total cholesterol, daily portions of fruit and vegetables, and body mass index) were calculated by linear regression. The dependent variable was the risk-factor level for each survey respondent; calendar year (i.e. year of interview) was the explanatory variable entered in the model as a continuous term. Absolute change was measured as the difference between the predicted values for 2000 and 2007, by age and sex.

Estimates of change in prevalence estimates (smoking, diabetes and physical activity) were calculated using a generalised linear model with binomial distribution and a log link function. The outcome variable was binary (1 indicating risk factor presence; 0 absence) with calendar year as the explanatory variable. The absolute difference in predicted values for 2007 and 2000 (ΔP in formula [4]) divided by the 2000 value provided the estimate of relative change.

Estimates of risk factor change were not calculated separately by deprivation quintile owing to small sample sizes, especially in those risk factors covered by the survey in intermittent years. Data since 2003 were weighted for non-response at each stage of data collection. Although it was just beyond the time period covered by the IMPACT_SEC_ model, the most recent survey data available (2008) was included in fitting the regressions to improve estimation of the underlying change. Analyses were conducted using Stata Version 11.1.

*Combining risk factors contributing positive and negative benefits to CHD mortality change*

In previous CHD modelling studies [14-15] adjusting for cumulative risk-reduction was straightforward as the trends in smoking, blood pressure and total cholesterol were mostly unidirectional: that is, risk factor levels were falling as were CHD mortality rates. However, in our current modelling study, mean BMI and the prevalence of diabetes increased over 2000-2007 while the five remaining risk factors showed favourable trends. In effect, the impact of risk factor change on CHD mortality was not uniformly beneficial: therefore, the proportional change in CHD mortality attributable to risk factor change was in some cases negative.

In order to avoid positive and negative R values cancelling each other out in the mathematical application of cumulative risk-reduction (formula [1] above), with the perverse effect of the cumulative benefits being apparently greater than the additive in some instances, we first converted all R values into absolute (i.e. sign-free) numbers. We did this on the understanding that the proportional change in CHD mortality associated with risk factor change was independent of the direction of change (e.g. a one unit increase in mean levels of fruit and vegetable consumption would result in a 4% fall in CHD mortality; likewise a one unit fall in consumption would result in a 4% increase). This meant that although the R values were not themselves ‘true’ indicators of the total proportional reduction in CHD mortality, both the additive and cumulative R values were computed on a like-for-like basis. Hence, the ratio of cumulative to additive risk reduction (the adjustment factor) was an accurate reflection of the degree to which the additive benefits needed to be adjusted down.

Age-sex-IMD specific adjustment factors (70 in total) were calculated by taking the ratio of cumulative to additive risk factor reduction. This involved five steps:

1. Regression equations were fitted to individual level survey data to derive the national (England) predicted risk factor levels for the start and end years of the model. Regression models were fitted separately by sex and ten-year age-bands (from ages 25 to 85+). (See section above describing the regression models fitted on the Health Survey for England data to estimate aggregate risk factor change).
2. The national predicted values for 2000 and 2007 were then graduated for increasing deprivation, using the SEC gradient calculated for each risk factor, based on pooled Health Survey for England data (see Table O). Multiplying the national predicted values by the SEC gradient resulted in a set of 70 age-sex-IMD specific estimates for 2000 and 2007, for each risk factor.
3. R values for continuous risk factors were then calculated using estimates of absolute change over 2000-2007 (formula [3]). R values for dichotomous risk factors were calculated by multiplying the estimated PARF in 2000 by the relative change in prevalence (formula [4]). This resulted in 70 R values (age-sex-IMD) for each risk factor. All R values were then converted into absolute numbers.
4. The absolute R values were then combined to calculate the additive (AR) and cumulative (CR) risk factor reductions (formulas [1] and [2] respectively). Age-sex-IMD specific adjustment factors were then calculated using the ratio CR/AR.
5. Multiplying through the age-sex-IMD specific additive DPPs in the model for each risk factor by the corresponding adjustment factor yielded the estimate of the cumulative benefit of risk factor change to CHD mortality decline over the seven year period, 2000 and 2007.

*Adjustment factors by age-sex-IMD*

The adjustment factors (shown in Table D) fell within the range of 0.83 to 0.96. The largest adjustment (0.83) was applied to the DPPs for women aged 65-74 resident in the most deprived areas (IMDQ5). The adjustment factors for the deprivation quintiles were, on average, ± 0.01 of the overall adjustment ratio for England across the 14 age and sex groups. The adjustments were on average, slightly higher for women (0.89) than men (0.92); and were higher in IMDQ5 than in IMDQ1 (mean values 0.8924 and 0.9089, respectively). Hence the adjustment values indicated a larger downward adjustment to the additive DPPs in the most deprived areas relative to the most affluent.

**1.4** OTHER METHODOLOGICAL CONSIDERATIONS

Other than calculations to take into account change in treatment uptake and risk factors over time, several other adjustments had to be made. These include:

- adjusting the relative reduction in the case fatality rate for persons receiving multiple treatments (poly-pharmacy),
- establishing rules for avoiding double-counting individuals belonging to more than one patient group,
- overlap between pharmacological and non-pharmacological contribution to risk factor change,
- uncertainty analyses,
- measuring net effects of changes in treatment uptake,
- allocating areas to quintiles by socioeconomic circumstances

These are discussed in turn below.

1.4.1 Accounting for poly-pharmacy

Persons with or at high risk of developing CHD may take a number of different medications. However, data from randomised clinical trials on efficacy of treatment combinations are sparse. Mant and Hicks suggested a method to estimate case fatality reduction by poly-pharmacy [18]. The adjustment is carried out in a step-by-step manner as set out in the example below. First the total effect is calculated using an inappropriate additive model, which is then adjusted using effect size calculation with an appropriate multiplicative model.

**Example 4: Estimation of reduced benefit if patient taking multiple medications (Mant and Hicks approach)**

*Adjustment for poly-pharmacy in secondary prevention post myocardial infarction in men aged 55-64 in the most affluent quintile*

Taking the example of secondary prevention post myocardial infarction, good evidence (Table F) suggests that, for each intervention, the relative reduction in case fatality is approximately: aspirin 15%, beta-blockers 23%, ACE inhibitors (ACE I) 20%, statins 22%, warfarin 22%, and rehabilitation 26%. Our best estimates for uptake in 2007 in these patients were respectively 69%, 59%, 68%, 83%, 3%, and 45%. Assuming a one-year case fatality rate of 0.013 for men aged 55-64 and a total of 15,068 men aged 55-64 residing in the most affluent quintile in 2007 the total DPPs, with no adjustment for poly-pharmacy, would be calculated as shown in the table below:

| **Secondary prevention post MI** | **Numbers**  **in 2007** | **Treatment uptake** | **Compliance** | **Relative risk reduction** | **One year case fatality** | **Unadjusted DPPs** |
| --- | --- | --- | --- | --- | --- | --- |
| **Treatment** | **A** | **B** | **C** | **D** | **E** | **(A × B × C × D × E)** |
| Aspirin | 15,068 | 0.69 | 70% | 0.15 | 0.013 | 14 |
| Beta blockers | 15,068 | 0.59 | 70% | 0.23 | 0.013 | 19 |
| ACE Inhibitors | 15,068 | 0.68 | 70% | 0.20 | 0.013 | 19 |
| Statins | 15,068 | 0.83 | 50% | 0.22 | 0.013 | 18 |
| Warfarin | 15,068 | 0.03 | 70% | 0.22 | 0.013 | 1 |
| Rehabilitation | 15,068 | 0.45 | 65% | 0.26 | 0.013 | 15 |
| **Total** |  |  |  |  |  | **85** |

The Mant and Hicks approach suggests that in individual patients receiving all these interventions, case fatality reduction is very unlikely to be simply additive. Instead, having considered the 15% case fatality reduction achieved by aspirin, the next medication, in this case a beta-blocker, can only reduce the residual case fatality (1-15%). Likewise, the subsequent addition of an ACE inhibitor can then only decrease the remaining case fatality, which will be 1 – [(1-0.15) × (1-0.23)]. The Mant and Hicks approach therefore suggests that a cumulative relative benefit can be estimated as follows:

Cumulative relative benefit = ***1 – [(1 – (uptake of drug A × relative reduction in case fatality rate for drug A)) × (1 – (uptake of drug B × relative reduction in case fatality rate for drug B)) × …. × (1 – (uptake of drug N × relative reduction in case fatality rate for drug N))]***

In considering appropriate treatments for post MI patients, applying relative risk reductions (RRR) for aspirin, beta-blockers, ACE I, statins, warfarin, and rehabilitation then gives the following cumulative relative benefit (using a weighted average of the age-sex specific uptake figures in each deprivation quintile):

=1 – [(1 – (aspirin_uptake_ × aspirin_RRR_)) × (1 – (beta blockers_uptake_ × beta blockers_RRR_)) × (1 – (ACE I_uptake_ × ACE I_RRR_)) × (1 – (statins_uptake_ × statins_RRR_)) × (1 – (warfarin_uptake_ × warfarin_RRR_)) × (1 – (rehabilitation_uptake_ × rehabilitation_RRR_))

= 1 – [(1 – (0.72 × 0.15)) × (1 – (0.54 × 0.23)) × (1 – (0.63 × 0.20)) × (1 – (0.78 × 0.22)) × (1 – (0.08 × 0.22)) × (1 – (0.45 × 0.26))]

= 1 – [(0.89) × (0.88) × (0.87) × (0.81) × (0.98) × (0.88)]

≈ 0.52 (i.e. a 52% lower case fatality)

This represented a 24% relative reduction, i.e. 1-(0.52/0.68) on the simple additive value of 68%, resulting in 24% fewer DPPs out of an original total of 85 DPPs (leaving an adjusted total of 65):

***Adjusted DPPs = unadjusted DPPs × (cumulative relative benefit / additive benefit)***

Adjusted DPPs = 85 × (0.52/0.68) ≈ 65

All treatment DPPs quoted in the results tables refer to the adjusted DPPs.

1.4.2 Potential overlaps between patient groups: avoiding double counting

There are potential overlaps between CHD patient groups (Table N). For example, approximately half the patients having CABG surgery have had a previous myocardial infarction, and approximately 30% of myocardial infarction survivors have or will go on to develop heart failure within 12 months. Overlap adjustments between CHD patient groups were made to ensure that the final groups could be considered mutually exclusive.

Patient overlaps for 2007 are shown in Figure N.1.

1.4.3 Overlap between pharmacological and non-pharmacological contributions to risk factor DPPs

Risk factor improvements, such as lower blood pressure or lower total cholesterol, may be achieved through medications, lifestyle changes, or a combination. In order to separate the DPPs from pharmacological versus non-pharmacological contributions to CHD mortality, we subtracted the DPPs calculated in the treatment (primary prevention) component of the model from the DPPs calculated in the risk factor component. That is, to estimate the impact of population-wide reduction in total cholesterol due to non-pharmacological change, we subtracted the estimated effect of statins for the primary prevention of CHD from the overall number of DPPs due to change in mean total cholesterol. Similarly, to estimate the impact of the population-wide reduction in SBP we subtracted the estimated effect of anti-hypertensive medication for primary prevention from the overall number of DPPs due to change in mean SBP levels.

1.4.4 Net effects

As all treatments were in use in 2000, the net benefit of an intervention in 2007 was calculated by subtracting the expected number of deaths prevented if the uptake rates in 2000 remained constant from the estimated number of deaths prevented calculated using the 2007 uptake rates. This is illustrated in the example below.

**Example 5: Net effects for treatments**

*Calculating net effects for clopidogrel use in STEMI cases in men aged 75-84 in the most affluent quintile*

With an estimated total of 1,440 men aged 75-84 in the most affluent quintile (of whom 40% were assumed to be STEMI cases), 89% uptake in 2007, a relative risk reduction of 3%, a one-year case fatality rate of 34%, and 100% compliance, the total number of DPPs in 2007 was calculated as:

***Patient numbers × treatment uptake_2007_ × compliance × relative mortality reduction × one year case fatality***

= (1,440 × 40%) × **89**% × 100% × 3% × 34% ≈ 5 DPPs

Applying the uptake rate in 2000 (31%) gave a total of 2 DPPs:

***Patient numbers × treatment uptake_2000_ × compliance × relative mortality reduction × one year case fatality***

= (1440 × 40%) × **31**% × 100% ×3% × 34% ≈ 2 DPPs

The net DPPs were therefore:

***Net DPPs = DPPs using uptake_2007_ – DPPs using uptake_2000_***

**=** 5 –2 = 3

The estimated changes in treatment uptake between 2000 and 2007 by deprivation quintile are shown in Table H.

1.4.5 Uncertainty analyses

We implemented uncertainty analysis in Excel using Ersatz (version 1.0 available at [http://www.epigear.com](http://www.epigear.com/)). This is an add-on which allows probabilistic bootstrapping in Excel. Ersatz allows repeated random draws from specified distributions for input variables and then calculates the 95% uncertainty intervals from the realised values of the output variable (deaths prevented or postponed). For the IMPACT_SEC_ model, we calculated the uncertainty intervals based on 1000 draws – taking the 95% uncertainty intervals from the 2.5^th^ and 97.5^th^ percentiles. The parameter distributions used for the input variables to the DPP calculations are shown in Table M. Worked examples using Ersatz are shown below Table M.

1.4.6 Model fit

Overall, the model could not explain 14% of the total deaths prevented (i.e. a shortfall of about 5,300 fewer CHD deaths unexplained by the model). However, the percentage unexplained varied by age, sex, and socio-economic circumstances. These are shown in Tables L.1- L.4 and Figure L.1.

1.4.7 Allocating areas to socioeconomic quintiles using the Index of Multiple Deprivation, 2007

The Index of Multiple Deprivation (IMD) is a composite index of relative deprivation at small area level based on seven domains: income; employment; health deprivation and disability; education, skills and training; barriers to housing and services; crime and disorder; and living environment [19]. The IMD 2007 score of all small areas in England (average population 1,500) were ranked in ascending order and grouped into equal quintiles (about 6,500 areas in each), with quintile one (IMDQ1) including the most affluent and quintile five (IMDQ5) the most deprived areas. Based on their postcode of residence, patients treated in hospital (e.g. recorded in Hospital Episode Statistics) or in the community (e.g. in the General Practice Research Database) were matched via their area of residence to the corresponding deprivation quintile by the data providers to protect patient anonymity. Mortality counts were similarly aggregated into deprivation quintiles by the Office for National Statistics before being released to us for research purposes.

As the IMD 2007 includes rates of premature total mortality in the health deprivation and disability domain, its use to quantify health inequalities risks a tautology. However UK studies have shown that removing the health domain had little effect on either the assignment of areas into their deprivation quintile or the relationship between area-based deprivation and health [20].

Conceptually, the IMD 2007 is a measure of deprivation, not a measure of affluence. Hence, areas with the lowest scores are not necessarily the most affluent; rather they have the lowest concentration of deprived people. In this paper for clarity and to easily distinguish between the extreme ends of the deprivation spectrum, we have used the term ‘most affluent’ and ‘most deprived’ rather than ‘least deprived’ and ‘most deprived’.

# Table A. Population and patient data sources used in the IMPACT_SEC_ model

| **Information** | **Source** |
| --- | --- |
| **Population data** |  |
| Population counts and CHD deaths stratified by age, sex, and Index of Multiple Deprivation quintiles | Office for National Statistics (ONS):  (*2000*: ICD9 410-414)  (*2007*: ICD10 I20-I25) |
| **Number of patients admitted to hospital**: | |
| Myocardial infarction (MI) | Hospital Episode Statistics (HES), ([www.hesonline.nhs.uk](http://www.hesonline.nhs.uk/)). Emergency admissions with a primary diagnosis of MI (ICD10: I21). The ratio of MI admissions to STEMI and nSTEMI cases taken as 40/60 [21]. Individual level data for 1998 to 2007 supplied by The NHS Information Centre (reference No ET2323). |
| Angina pectoris | HES. Emergency admissions with a primary diagnosis of angina pectoris (ICD10: I20). |
| Heart failure | HES. Admissions with a primary diagnosis of heart failure (ICD10: I50). |
| **Number of patients undergoing revascularisation:** | |
| CABG | HES. OPCS Classification of Surgical Operations and Procedures – Fourth Revision (OPCS-4) K40-K46. |
| PCI | HES. OPCS Classification of Surgical Operations and Procedures – Fourth Revision (OPCS-4) OPCS K49, K50.1, K75. |
| **Patients in the community eligible for secondary prevention therapies:** | |
| - Post MI - Angina without MI - Heart failure | General Practice Research Database (GPRD) (http://www.cprd.com/home). |
| - Cardiac rehabilitation | Number enrolled in Cardiac Rehabilitation programmes adapted from the National Audit of Cardiac Rehabilitation ([http://www.cardiacrehabilitation.org.uk](http://www.cardiacrehabilitation.org.uk/)) [22] |
| **Patients eligible for primary prevention therapies:** | |
| Lipid-lowering drugs | Prevalence of never having had angina or heart attack and currently taking lipid lowering drugs prescribed by a doctor from the Health Survey for England (HSfE 1998, 2003, and 2006) (<http://www.ic.nhs.uk/statistics-and-data-collections/health-and-lifestyles-related-surveys/health-survey-for-england>) |
| Hypertension treatment | Prevalence of never having had angina or heart attack and currently taking medication specifically prescribed to treat high blood pressure from the Health Survey for England (HSfE 1998, 2003, and 2006) |

Table B. Data sources for treatment uptake levels

**Medical and surgical treatments included in the model**

| **Information** | **Source** |
| --- | --- |
| **Medication use in hospital**: |  |
| **(ST-segment elevation myocardial infarction)**:   - Aspirin - Beta Blockers - ACE I or Angiotensin-II receptor antagonists (ARB) - Thrombolysis - Clopidogrel | Myocardial Ischaemia National Audit Project (MINAP) 2003 to 2007. (http://www.ucl.ac.uk/nicor/audits/minap). STEMI cases defined by the final diagnosis field (includes those with threatened infarction). |
| **Non-ST-segment elevation acute coronary syndrome (NSTEACS):**   - Aspirin without heparin - Aspirin & heparin - Platelet glycoprotein IIB/IIIA inhibitors - Beta Blockers - ACE I/ARB - Clopidogrel | MINAP 2003 to 2007. nSTEMI cases defined by the final diagnosis field. |
| **Heart failure due to CHD:**   - Aspirin^†^ - Beta blockers^††^ - ACE I/ARB^††^ - Spironolactone^††^ | ^†^ Assumed equal to post MI rates in the community obtained using the General Practice Research Database (GPRD) (http://www.cprd.com/home).  ^††^ NHS Heart Failure Survey 2005 [23]. Start year values for beta-blockers, ACE I/ARB and spironolactone assumed to be to 10% lower than 2005 values. |
| **In-hospital cardio-pulmonary resuscitation (CPR)** | MINAP 2003 to 2007. |
| **CPR in the community** | Net benefits assumed to be zero. |
| **Cardiac rehabilitation for MI and revascularisation survivors** | Number enrolled in cardiac rehabilitation programmes adapted from the National Audit of Cardiac Rehabilitation ([http://www.cardiacrehabilitation.org.uk](http://www.cardiacrehabilitation.org.uk/)) [22] |
| **Medication use in the community:**  **Post MI and revascularisation survivors, chronic stable coronary artery disease (CAD), heart failure**   - Aspirin - Beta blockers - ACE I/ARB - Statins - Warfarin - Spironolactone | General Practice Research Database (GPRD) (http://www.cprd.com/home). |
| **Primary prevention therapies:** | |
| **Lipid-lowering drugs** | Prevalence of never having had angina or heart attack and currently taking lipid lowering drugs prescribed by a doctor from the Health Survey for England (HSfE 1998, 2003, and 2006). |
| **Anti-hypertensive medication** | Prevalence of never having had angina or heart attack and currently taking medication specifically prescribed to treat high blood pressure from the Health Survey for England (HSfE 1998, 2003, and 2006). |
|  |  |

# Table C. Risk factors – variable definitions and source

The Health Survey for England (HSfE), an annual nationwide household survey of the English population, has been described in detail elsewhere [24]. Briefly, members of a stratified random sample (drawn from the Postcode Address File) that is socio-demographically representative of the English population were invited to participate. The annual household response rate was 75% in 2000, falling steadily to 66% in 2007. Data were collected at two visits: an interviewer’s visit, during which a questionnaire was administered, followed by a visit from a trained nurse for all those interviewed who agreed. The nurse visit, which did not take place in 2004 among the general population sample, includes measurements and collection of blood, as well as additional questioning including use of prescribed medication (1998, 2003, and 2006). The magnitude of risk factor change from 2000 to 2007 used for the calculation of DPPs (see Examples 2 and 3) was estimated using a ‘fixed gradient’ approach across deprivation quintiles to maximise precision. For details on this approach see Table O. Risk factor levels in 2000 and 2007 by gender and deprivation quintiles are shown in Table K.

| **Risk factor** | **HSfE survey years** | **Description** |
| --- | --- | --- |
| **Current cigarette smoking** | 2000-7 | Self-reported status |
| **SBP (mmHg)** | All years between 2000-7 except 2004 | Calculated as the mean of the 2^nd^ and 3^rd^ readings for those who had not eaten, consumed alcohol or smoked in the 30 minutes prior to measurement. Those reporting taking blood pressure lowering drugs were included |
| **Body Mass Index** | 2000-7 | Weight (kg) divided by height squared (m^2^) for all respondents with valid height and weight measurements. |
| **Total cholesterol (mmol/l)** | 1998,2003,2006 | Those reporting taking lipid lowering drugs were included |
| **Diabetes** | 1998,2003,2006 | Those reporting diabetes that was doctor-diagnosed, excluding women who had only had diabetes during pregnancy |
| **Physical activity** | 1998,2003,2006 | High levels defined as spending 30 minutes or more of moderate or vigorous activity on at least five days per week. Occupational activity was excluded. |
| **Fruit and vegetable consumption** | 2001-7 | Measured in portions per day |

# Table D. Cumulative benefit: Adjustment factors by age, sex and IMD quintile

In Section 1.3 we described how we adjusted down the DPPs calculated in an additive fashion over the seven risk factors by using the ratio of cumulative to additive risk-reduction. The 70 age-sex-IMD specific adjustment factors are shown below.

|  | **Deprivation quintile** | | | | |  |
| --- | --- | --- | --- | --- | --- | --- |
|  | **IMDQ1** | **IMDQ2** | **IMDQ3** | **IMDQ4** | **IMDQ5** | **England** |
| **Men:** |  |  |  |  |  |  |
| **25-34** | 0.9464 | 0.9449 | 0.9463 | 0.9462 | 0.9434 | 0.9453 |
| **35-44** | 0.9196 | 0.9169 | 0.9179 | 0.9126 | 0.9110 | 0.9153 |
| **45-54** | 0.9335 | 0.9278 | 0.9205 | 0.9193 | 0.9083 | 0.9219 |
| **55-64** | 0.8957 | 0.8957 | 0.8883 | 0.8851 | 0.8762 | 0.8886 |
| **65-74** | 0.8885 | 0.8843 | 0.8846 | 0.8817 | 0.8720 | 0.8827 |
| **75-84** | 0.9182 | 0.9146 | 0.9134 | 0.9214 | 0.9149 | 0.9162 |
| **85+** | 0.9561 | 0.9569 | 0.9525 | 0.9520 | 0.9582 | 0.9547 |
| **Women:** |  |  |  |  |  |  |
| **25-34** | 0.8799 | 0.8872 | 0.8846 | 0.8787 | 0.8782 | 0.8809 |
| **35-44** | 0.9148 | 0.9119 | 0.9014 | 0.9034 | 0.8892 | 0.9038 |
| **45-54** | 0.9038 | 0.9013 | 0.8937 | 0.8777 | 0.8546 | 0.8865 |
| **55-64** | 0.8862 | 0.8896 | 0.8842 | 0.8703 | 0.8560 | 0.8780 |
| **65-74** | 0.8620 | 0.8569 | 0.8523 | 0.8363 | 0.8307 | 0.8479 |
| **75-84** | 0.8803 | 0.8869 | 0.8824 | 0.8778 | 0.8622 | 0.8779 |
| **85+** | 0.9394 | 0.9399 | 0.9409 | 0.9463 | 0.9386 | 0.9410 |
| **Overall** | 0.9089 | 0.9082 | 0.9045 | 0.9006 | 0.8924 | 0.9029 |

# Table E. CHD mortality rates 2000 and 2007 by sex and deprivation quintiles

|  | **Year** | **England** | **IMDQ1** | **IMDQ2** | **IMDQ3** | **IMDQ4** | **IMDQ5** |
| --- | --- | --- | --- | --- | --- | --- | --- |
| **Male** |  |  |  |  |  |  |  |
| **Population (000s)** | **2000** | 16242 | 3353 | 3372 | 3321 | 3186 | 3011 |
|  | **2007** | 17002 | 3525 | 3542 | 3486 | 3335 | 3114 |
| **Observed CHD deaths** | **2000** | 56713 | 9146 | 10868 | 11671 | 12094 | 12934 |
|  | **2007** | 41713 | 6962 | 8129 | 8535 | 8723 | 9364 |
| **Age-standardised rate (00,000)** | **2000** | 310 | 238 | 270 | 301 | 349 | 415 |
|  | **2007** | 200 | 147 | 170 | 191 | 231 | 294 |
| **Annual % fall^†^** |  | 6.0 | 6.6 | 6.4 | 6.3 | 5.7 | 4.8 |
| **Expected deaths^††^** | **2007** | 63685 | 11207 | 12856 | 13348 | 13098 | 13176 |
| **Target DPPs^‡^** | **2007** | 21972 | 4245 | 4727 | 4813 | 4375 | 3812 |
| **% of expected deaths prevented** | **2007** | 34.5 | 37.9 | 36.8 | 36.1 | 33.4 | 28.9 |
| **Female** |  |  |  |  |  |  |  |
| **Population (000s)** | **2000** | 17710 | 3618 | 3663 | 3618 | 3493 | 3318 |
|  | **2007** | 18279 | 3803 | 3820 | 3747 | 3571 | 3337 |
| **Observed CHD deaths** | **2000** | 46530 | 7383 | 8959 | 9789 | 10093 | 10306 |
|  | **2007** | 32461 | 5350 | 6315 | 6812 | 6953 | 7031 |
| **Age-standardised rate (00,000)** | **2000** | 148 | 115 | 128 | 143 | 164 | 198 |
|  | **2007** | 94 | 70 | 79 | 90 | 107 | 136 |
| **Annual % fall^†^** |  | 6.3 | 6.7 | 6.7 | 6.4 | 5.9 | 5.2 |
| **Expected deaths^††^** | **2007** | 48559 | 8458 | 9812 | 10348 | 10162 | 9778 |
| **Target DPPs^‡^** | **2007** | 16098 | 3108 | 3497 | 3536 | 3209 | 2747 |
| **% of expected deaths prevented** | **2007** | 33.2 | 36.7 | 35.6 | 34.2 | 31.6 | 28.1 |
| **Total** |  |  |  |  |  |  |  |
| **Population (000s)** | **2000** | 33952 | 6972 | 7035 | 6939 | 6678 | 6329 |
|  | **2007** | 35281 | 7328 | 7363 | 7233 | 6906 | 6451 |
| **Observed CHD deaths** | **2000** | 103243 | 16529 | 19827 | 21460 | 22187 | 23240 |
|  | **2007** | 74174 | 12312 | 14444 | 15347 | 15676 | 16395 |
| **Age-standardised rate (00,000)** | **2000** | 229 | 177 | 199 | 222 | 257 | 306 |
|  | **2007** | 147 | 109 | 124 | 141 | 169 | 215 |
| **Annual % fall^†^** |  | 6.1 | 6.7 | 6.5 | 6.3 | 5.8 | 4.9 |
| **Expected deaths^††^** | **2007** | 112244 | 19665 | 22669 | 23696 | 23260 | 22953 |
| **Total DPPs^‡^** | **2007** | 38070 | 7353 | 8225 | 8349 | 7584 | 6558 |
| **% of expected deaths prevented** | **2007** | 33.9 | 37.4 | 36.3 | 35.2 | 32.6 | 28.6 |

^†^ Annual % fall = (1-(2007 rate/2000 rate)^(1/7))

^††^ Expected deaths = CHD deaths expected in 2007 had 2000 CHD rates remained.

^‡^ DPPs, deaths prevented or postponed. DPPs = expected – observed deaths in 2007

# Table F. Clinical efficacy of interventions: relative risk reductions obtained from meta-analyses, and randomised clinical trials

| **Treatments** | **Relative risk reduction^†^** | **Comments** | **Source paper: First author (year), notes** |
| --- | --- | --- | --- |
| ***ST elevation myocardial infarction (STEMI)*** | | | |
|  |  |  |  |
| **Thrombolysis** | 31% (95% CI: 14,45) | <55 years: Odds Ratio (OR)=0.692; Relative Risk Reduction (RRR)=30.8% (95% CI: 14,45)  55-64 years: OR=0.736; RRR=26.4% (95% CI: 17,40)  65-74 years: OR=0.752; RRR=24.8% (95% CI: 15,37)  > 75 years: OR=0.844; RRR=15.6% (95% CI: 4,30) | Estess (2002) [25] |
| **Aspirin** | 23% (95% CI: 15,30) | RRR=23% (95% CI: 15,30): outcome is vascular deaths | ISIS-2 (1988) [26] |
| **Primary CABG surgery** | 39% (95% CI: 23,52) | OR=0.61 (95% CI: 0.48,0.77); RRR=39% (95% CI: 23,52) on page 565, 0-5 year mortality | Yusuf (1994) [27] |
| **Primary PCI** | 30% (95% CI: 15,42) | OR=0.70 (95% CI: 0.58,0.85); RRR=30% (95% CI: 15,42) outcome compares primary angioplasty to thrombolytics. | Keeley (2003) [28] |
| **Beta blockers** | 4% (95% CI: -8,15) | OR=0.96 (95% CI: 0.85,1.08); RRR=4% (95% CI: -8,15) on page 1732 | Freemantle (1999) [29] |
| **ACE inhibitors** | 7% (95% CI: 2,11) | OR=0.93 (95% CI: 0.89,0.98); RRR=7% (95% CI: 2,11) for 30 day mortality in myocardial infarction | ACE Inhibitor Myocardial Infarction Collaborative Group (1998) [30] |
| **Clopidogrel** | 3% (95% CI: 1,6) | RRR=3% (95% CI: 1,6) for 30 day mortality in myocardial infarction | Chen (2005) [31]  Sabatine (2005) [32] |
| **Hospital CPR** | 33% (95% CI: 10,36) | Survival at 24 hours estimated to be 32%, discharge to home at 21%, and 1 year survival to be 15% overall. | Tunstall-Pedoe (1992) [33]  Nadkarni (2006) [34] |
| ***Non-ST-segment elevation acute coronary syndrome (NSTEACS):*** | | | |
|  |  |  |  |
| **Aspirin alone** | 15% (95% CI: 11,19) | OR=0.85 (95% CI: 0.49,0.95); RRR=15% (95% CI: 11,19). Outcome is vascular and nonvascular deaths on page 75. Assume appropriate for patients with NSTE-ACS. | Antithrombotic Trialists’ Collaboration (2002) [35] |
| **Aspirin & heparin** | 33% (95% CI: -2,56) | OR=0.67 (95% CI: 0.48,1.02); RRR=33% (95% CI: -2,56%) in Table 2. The study outcome is composite MI death and non-fatal MI; compares those on aspirin & heparin to aspirin only. | Oler (1996) [36] |
| **Platelet glycoprotein IIB/IIIA inhibitors** | 9% (95% CI: 2,16) | OR=0.91 (95% CI: 0.84,0.98); RRR=9% (95% CI: 2,16). Study looked at acute coronary syndrome without persistent ST elevation. | Boersma (2002) [37] |
| **Early PCI** | 32% (95% CI: 5,51) | OR=0.68 (95% CI: 0.49,0.95); RRR=32% (95% CI: 5,51) | RITA 3 (Fox 2005) [38] |
| **Primary CABG surgery** | 39% (95% CI: 23,52) | OR=0.61 (95% CI: 0.48,0.77); RRR=39% (95% CI: 23,52) on page 565, 0-5 year mortality | Yusuf (1994) [27],  assumed similar as STEMI. |
| **Clopidogrel** | 7% (95% CI: 2,11) | RRR=7% (95% CI: 2,11) | Yusuf (2001) [39] |
| **Beta blockers** | 4% (95% CI: -8,15) | OR=0.96 (95% CI: 0.85,1.08); RRR=4% (95% CI: -8,15) on page 1732 | Freemantle (1999) [29],  assumed similar as STEMI. |
| **ACE inhibitors** | 7% (95% CI: 2,11) | OR=0.93 (95% CI: 0.89,0.98); RRR=7% (95% CI: 2,11) for 30 day mortality in myocardial infarction | ACE Inhibitor Myocardial Infarction Collaborative Group (1998) [30] |
| ***Secondary prevention post myocardial infarction/revascularisation:*** | | | |
|  |  |  |  |
| **Aspirin** | 15% (95% CI: 11,19) | OR=0.85 (95% CI: 0.49,0.95); RRR=15% (95% CI: 11,19). Outcome is vascular and nonvascular deaths on page 75. This data seems to be appropriate to this outcome in CHD patients. | Antithrombotic Trialists’ Collaboration (2002) [35] |
| **Beta blockers** | 23% (95% CI: 15,31) | OR=0.77 (95% CI: 0.69,0.85); RRR=23% (95% CI: 15,31) on page 1734. Odds of death in long term trials. | Freemantle (1999) [29] |
| **ACE inhibitors or Angiotensin-II receptor antagonists** | 20% (95% CI: 13,26) | OR=0.80 (95% CI: 0.74,0.87); RRR=20% (95% CI: 13,26) on page 1577, death up to four years [endpoint of study looking at those with heart failure or LV dysfunction]. | Flather (2000) [40] |
| **Statins** | 24% (95% CI: 10,26) | RRR=24% (95% CI: 10,26)  Intensive statin therapy in acute coronary syndromes. | Hulten (2006) [41] |
| **Warfarin** | 22% (95% CI: 13,31) | OR=0.78 (95% CI: 0.67,0.90); RRR=22% (95% CI: 10,33) | Anand and Yusuf (1999) [42] |
| **Rehabilitation** | 26% (95% CI: 10,39) | OR=0.74 (95% CI: 0.61,0.90); RRR=26% (95% CI: 10,39) in Figure 1, page 685 Taylor reference | Taylor (2004) [43] |
| ***Chronic stable coronary artery disease:*** | | | |
|  |  |  |  |
| **CABG surgery**  **years 0-5** | 39% (95% CI:23,52) | OR = 0.61 (95% CI: 0.48-0.77), RRR 39% (95% CI: 23,52) on page 565, 5 year mortality | Yusuf (1994) [27] |
| **CABG surgery**  **years 6-10** | 32% (95% CI: 2,30) | OR = 0.83 (95% CI: 0.70-0.98), RRR 17% (95% CI: 2,30) on page 565, 10 year mortality.  OR = 0.68 (95% CI: 0.56-0.83), RRR 32% (95% CI: 17,44) on page 565, 7 year mortality  CABG compared to medical treatment | Yusuf (1994) [27] |
| **Angioplasty** | No effect |  | Boden (2007) [44] |
| **Aspirin** | 15% (95% CI: 11,19) | OR=0.85 (95% CI: 0.49-0.95); RRR=15% (95% CI: 11,19). Outcome is vascular and nonvascular deaths on page 75. | Antithrombotic Trialists’ Collaboration (2002) [35] |
| **Statins** | 23% (95% CI: 10,26) | RRR=23% (95% CI 10,26)  Standard dose statin therapy in coronary artery disease. | Wilt (2004) [45] |
| **ACE inhibitors/ARB** | 17% (95% CI: 6,28) | RRR=17% (95% CI 6,28) | Al-Mallah (2006) [46] |
| ***Heart failure in patients requiring hospitalisation or in the community:*** | | | |
|  |  |  |  |
| **ACE inhibitors** | 20% (95% CI: 13,26) | OR=0.80 (95% CI: 0.74,0.87); RRR=20% (95% CI: 13,26) on page 1577 [death up to four years was study endpoint for those with heart failure or LV dysfunction] | Flather (2000) [40] |
| **Beta blockers** | 35% (95% CI: 26,43) | OR=0.65 (95% CI: 0.57,0.74); RRR=35% (95% CI: 26,43): all cause mortality | Shibata (2001) [47] |
| **Spironolactone** | 30% (95% CI: 18,41)  31% (95% CI: 18,42) | OR=0.70 (95% CI: 0.59,0.82); RRR=30% (95% CI: 18,41) in those that had at least one cardiac related hospitalisation.  OR=0.69 (95% CI: 0.58,0.82); RRR=31% (95% CI: 18,42) in entire study population consisting of those with community heart failure, page 711. | Pitt (1999) [48] |
| **Aspirin** | 15% (95% CI: 11,19) | OR=0.85 (95% CI: 0.49,0.95); RRR=15% (95% CI: 11,19). Outcome is vascular and nonvascular deaths on page 75. | Antithrombotic Trialists’ Collaboration (2002) [35] |
| **Statins** | No effect |  | Kjekshus (2007) [49]  Tavazzi (2008) [50] |
| ***Primary prevention therapies:*** | | | |
|  |  |  |  |
| **Treatments for high blood pressure** | 13% (95% CI: 6,19) | OR=0.87 (95% CI: 0.81,0.94); RRR=13% (95% CI: 6,19) in those with high blood pressure without disease at entry. [RRR=29% (95% CI: 17,37) those with average blood pressure and CHD, treated with ACE inhibitors] | Law (2003) [51] |
| **Statins** | 35% (95% CI: 11,52) | OR=0.65 (95% CI: 0.48,0.89); RRR=35% (95% CI: 11,52) for CHD mortality (only trials using statins), Figure 3 on page 4 | Pignone (2000) [52] |

^†^Relative risk reduction (RRR) calculated as 1 – odds ratio

# Table G. Case fatality rates for each patient group

| **Patient group** | **AMI** | **POST AMI** | **Acute coronary syndrome (ACS)** | **Post revascularisation** | **Chronic stable coronary artery disease** | **Heart failure in hospital** | **Heart failure in community** | **Hypertension** | **Hyper-cholesteraemia** |
| --- | --- | --- | --- | --- | --- | --- | --- | --- | --- |
| **Interval** | **30 day** | **1 year** | **1 year** | **1 year** | **1 year** | **1 year** | **1 year** | **1 year** | **1 year** |
| **Men:** |  |  |  |  |  |  |  |  |  |
| **25-34** | 0.03 | 0.009 | 0.01 | 0.250 | 0.006 | 0.14 | 0.04 | 0.000 | 0.000 |
| **35-44** | 0.02 | 0.006 | 0.01 | 0.050 | 0.009 | 0.14 | 0.04 | 0.001 | 0.001 |
| **45-54** | 0.03 | 0.006 | 0.02 | 0.020 | 0.012 | 0.13 | 0.06 | 0.002 | 0.002 |
| **55-64** | 0.06 | 0.013 | 0.03 | 0.030 | 0.016 | 0.22 | 0.08 | 0.006 | 0.006 |
| **65-74** | 0.16 | 0.027 | 0.05 | 0.045 | 0.029 | 0.34 | 0.13 | 0.014 | 0.014 |
| **75-84** | 0.34 | 0.067 | 0.11 | 0.078 | 0.065 | 0.44 | 0.20 | 0.035 | 0.035 |
| **85+** | 0.51 | 0.189 | 0.26 | 0.194 | 0.163 | 0.61 | 0.32 | 0.094 | 0.094 |
|  |  |  |  |  |  |  |  |  |  |
| **Women:** |  |  |  |  |  |  |  |  |  |
| **25-34** | 0.03 | 0.008 | 0.01 | 0.000 | 0.007 | 0.50 | 0.05 | 0.000 | 0.000 |
| **35-44** | 0.05 | 0.008 | 0.01 | 0.000 | 0.007 | 0.17 | 0.05 | 0.001 | 0.001 |
| **45-54** | 0.06 | 0.011 | 0.02 | 0.033 | 0.010 | 0.06 | 0.05 | 0.002 | 0.002 |
| **55-64** | 0.11 | 0.014 | 0.02 | 0.044 | 0.014 | 0.24 | 0.08 | 0.004 | 0.004 |
| **65-74** | 0.18 | 0.028 | 0.05 | 0.064 | 0.025 | 0.31 | 0.12 | 0.014 | 0.014 |
| **75-84** | 0.30 | 0.052 | 0.10 | 0.084 | 0.054 | 0.39 | 0.17 | 0.035 | 0.035 |
| **85+** | 0.49 | 0.177 | 0.19 | 0.083 | 0.155 | 0.37 | 0.30 | 0.094 | 0.094 |
|  |  |  |  |  |  |  |  |  |  |

**Source:** Wijeysundera et.al (2010) [5]

# Table H. Treatment uptake in 2000 and 2007^†^

|  | **England** |  | | **IMDQ1** | | | **IMDQ2** | | | **IMDQ3** | | | **IMDQ4** | | | **IMDQ5** | | |
| --- | --- | --- | --- | --- | --- | --- | --- | --- | --- | --- | --- | --- | --- | --- | --- | --- | --- | --- |
|  | **N** | **Uptake (%)** | | **N** | **Uptake (%)** | | **N** | **Uptake (%)** | | **N** | **Uptake (%)** | | **N** | **Uptake (%)** | | **N** | **Uptake (%)** | |
|  |  | **2000** | **2007** |  | **2000** | **2007** |  | **2000** | **2007** |  | **2000** | **2007** |  | **2000** | **2007** |  | **2000** | **2007** |
| ***ST elevation myocardial infarction (STEMI):*** | | | | | | | | | | | | | | | | | | |
| Thrombolysis | 20,702 | 77.2 | 56.7 | 3,667 | 79.4 | 58.6 | 4,155 | 77.9 | 59.5 | 4,267 | 75.4 | 57.1 | 4,264 | 76.2 | 56.0 | 4,350 | 77.4 | 52.5 |
| Aspirin | 20,702 | 93.6 | 96.0 | 3,667 | 93.6 | 96.6 | 4,155 | 94.7 | 96.3 | 4,267 | 93.1 | 95.4 | 4,264 | 93.2 | 95.6 | 4,350 | 93.4 | 96.4 |
| B-Blocker | 20,702 | 71.3 | 70.3 | 3,667 | 74.8 | 70.9 | 4,155 | 72.3 | 69.1 | 4,267 | 71.0 | 69.8 | 4,264 | 69.6 | 69.5 | 4,350 | 69.9 | 72.4 |
| ACE I/ARB | 20,702 | 77.2 | 76.3 | 3,667 | 79.8 | 76.6 | 4,155 | 78.9 | 75.6 | 4,267 | 75.4 | 75.5 | 4,264 | 75.4 | 74.8 | 4,350 | 77.3 | 79.2 |
| Clopidogrel | 20,702 | 27.7 | 88.5 | 3,667 | 26.9 | 88.7 | 4,155 | 25.7 | 87.7 | 4,267 | 28.0 | 88.4 | 4,264 | 28.5 | 88.4 | 4,350 | 28.9 | 89.2 |
| Primary PCI | 20,702 | 3.9 | 23.7 | 3,667 | 2.9 | 24.2 | 4,155 | 3.4 | 21.8 | 4,267 | 3.8 | 23.3 | 4,264 | 4.3 | 24.5 | 4,350 | 4.8 | 24.8 |
| Primary CABG | 20,702 | 0.1 | 0.1 | 3,667 | 0.1 | 0.1 | 4,155 | 0.0 | 0.1 | 4,267 | 0.1 | 0.1 | 4,264 | 0.1 | 0.2 | 4,350 | 0.0 | 0.1 |
| In hospital CPR^††^ | 20,702 | 11.4 | 6.6 | 3,667 | 9.9 | 6.5 | 4,155 | 11.6 | 7.1 | 4,267 | 11.7 | 6.3 | 4,264 | 11.8 | 6.7 | 4,350 | 11.6 | 6.3 |
| ***Non-ST-segment elevation acute coronary syndrome (NSTEACS):*** | | | | | | | | | | | | | | | | | | |
| Aspirin & heparin | 91,288 | 64.0 | 79.7 | 14,653 | 67.1 | 79.7 | 17,062 | 65.0 | 80.3 | 18,257 | 66.7 | 79.9 | 19,437 | 65.7 | 80.2 | 21,878 | 57.9 | 78.8 |
| Aspirin alone | 91,288 | 24.2 | 12.8 | 14,653 | 21.5 | 13.5 | 17,062 | 23.9 | 12.3 | 18,257 | 21.5 | 12.7 | 19,437 | 23.5 | 12.6 | 21,878 | 28.9 | 13.1 |
| Platelet glycoprotein IIB/IIIA inhibitors | 91,288 | 6.1 | 5.8 | 14,653 | 9.4 | 6.2 | 17,062 | 7.3 | 5.8 | 18,257 | 6.1 | 5.1 | 19,437 | 4.7 | 5.2 | 21,878 | 4.3 | 6.8 |
| ACE I/ARB | 91,288 | 66.0 | 73.2 | 14,653 | 68.6 | 73.1 | 17,062 | 64.5 | 72.2 | 18,257 | 65.9 | 72.5 | 19,437 | 64.3 | 72.7 | 21,878 | 67.0 | 75.0 |
| B-Blocker | 91,288 | 63.2 | 67.6 | 14,653 | 66.1 | 68.2 | 17,062 | 62.7 | 67.7 | 18,257 | 63.5 | 66.7 | 19,437 | 61.7 | 66.3 | 21,878 | 62.8 | 69.2 |
| Clopidogrel | 91,288 | 44.3 | 86.6 | 14,653 | 43.5 | 87.1 | 17,062 | 44.2 | 86.9 | 18,257 | 42.3 | 86.8 | 19,437 | 45.6 | 85.9 | 21,878 | 45.4 | 86.3 |
| CABG (< 6 weeks) | 91,288 | 3.0 | 2.6 | 14,653 | 3.5 | 3.4 | 17,062 | 3.2 | 2.7 | 18,257 | 3.2 | 2.6 | 19,437 | 2.9 | 2.3 | 21,878 | 2.5 | 2.1 |
| PCI (0-14 days) | 91,288 | 3.1 | 6.7 | 14,653 | 3.6 | 7.7 | 17,062 | 3.4 | 6.9 | 18,257 | 3.2 | 7.0 | 19,437 | 2.9 | 6.4 | 21,878 | 2.5 | 5.7 |
| In hospital CPR (nSTEMI only)^††^ | 31,053 | 5.3 | 2.3 | 5,500 | 4.6 | 2.3 | 6,233 | 4.9 | 2.2 | 6,400 | 5.3 | 2.1 | 6,395 | 5.6 | 2.5 | 6,524 | 5.8 | 2.5 |
| ***Secondary prevention post myocardial infarction:*** | | | | | | | | | | | | | | | | | | |
| Aspirin | 565,592 | 59.7 | 74.4 | 99,403 | 56.4 | 72.4 | 116,190 | 60.0 | 74.3 | 115,527 | 59.2 | 74.5 | 114,807 | 58.8 | 74.8 | 119,665 | 63.3 | 75.8 |
| B-Blocker | 565,592 | 32.6 | 53.4 | 99,403 | 34.0 | 54.0 | 116,190 | 34.0 | 54.6 | 115,527 | 31.6 | 53.3 | 114,807 | 32.2 | 52.9 | 119,665 | 31.7 | 52.4 |
| ACE I/ARB | 565,592 | 31.3 | 62.0 | 99,403 | 32.3 | 62.6 | 116,190 | 32.5 | 62.3 | 115,527 | 31.0 | 61.6 | 114,807 | 30.6 | 61.2 | 119,665 | 30.5 | 62.5 |
| Statin | 565,592 | 37.1 | 77.4 | 99,403 | 39.8 | 77.9 | 116,190 | 39.5 | 77.8 | 115,527 | 35.9 | 76.6 | 114,807 | 34.7 | 76.6 | 119,665 | 36.2 | 78.1 |
| Warfarin | 565,592 | 6.6 | 8.1 | 99,403 | 7.7 | 8.3 | 116,190 | 6.7 | 8.9 | 115,527 | 6.5 | 7.9 | 114,807 | 6.2 | 7.7 | 119,665 | 6.2 | 7.6 |
| Rehabilitation | 565,592 | 45.0 | 45.0 | 99,403 | 45.0 | 45.0 | 116,190 | 45.0 | 45.0 | 115,527 | 45.0 | 45.0 | 114,807 | 45.0 | 45.0 | 119,665 | 45.0 | 45.0 |
| ***Secondary prevention post revascularisation:*** | | | | | | | | | | | | | | | | | | |
| Aspirin | 111,930 | 64.3 | 76.5 | 21,442 | 58.8 | 73.5 | 22,773 | 63.5 | 76.2 | 23,274 | 62.5 | 76.5 | 22,574 | 65.7 | 76.1 | 21,868 | 71.2 | 80.0 |
| B-Blocker | 111,930 | 30.7 | 55.7 | 21,442 | 29.2 | 52.7 | 22,773 | 30.5 | 55.6 | 23,274 | 29.8 | 56.4 | 22,574 | 31.7 | 56.9 | 21,868 | 32.3 | 56.9 |
| ACE I/ARB | 111,930 | 30.1 | 64.2 | 21,442 | 30.8 | 63.9 | 22,773 | 29.2 | 63.0 | 23,274 | 29.5 | 63.9 | 22,574 | 30.9 | 63.4 | 21,868 | 30.5 | 67.1 |
| Statin | 111,930 | 58.2 | 84.5 | 21,442 | 61.7 | 85.1 | 22,773 | 58.2 | 84.6 | 23,274 | 56.3 | 83.9 | 22,574 | 56.3 | 84.8 | 21,868 | 58.7 | 84.2 |
| Warfarin | 111,930 | 7.4 | 6.7 | 21,442 | 7.9 | 7.2 | 22,773 | 7.3 | 6.6 | 23,274 | 6.7 | 6.5 | 22,574 | 7.1 | 7.1 | 21,868 | 7.9 | 6.1 |
| Rehabilitation (CABG) | 32,151 | 73.0 | 73.0 | 6,071 | 73.0 | 73.0 | 6,758 | 73.0 | 73.0 | 6,877 | 73.0 | 73.0 | 6,425 | 73.0 | 73.0 | 6,020 | 73.0 | 73.0 |
| Rehabilitation (PCI) | 61,672 | 10.0 | 20.0 | 11,912 | 10.0 | 20.0 | 12,348 | 10.0 | 20.09 | 12,666 | 10.0 | 20.0 | 12,457 | 10.0 | 20.0 | 12,290 | 10.0 | 20.0 |

|  | **England** |  | | **IMDQ1** | | | **IMDQ2** | | | **IMDQ3** | | | **IMDQ4** | | | **IMDQ5** | | |
| --- | --- | --- | --- | --- | --- | --- | --- | --- | --- | --- | --- | --- | --- | --- | --- | --- | --- | --- |
|  | **N** | **Uptake (%)** | | **N** | **Uptake (%)** | | **N** | **Uptake (%)** | | **N** | **Uptake (%)** | | **N** | **Uptake (%)** | | **N** | **Uptake (%)** | |
|  |  | **2000** | **2007** |  | **2000** | **2007** |  | **2000** | **2007** |  | **2000** | **2007** |  | **2000** | **2007** |  | **2000** | **2007** |
| ***Chronic stable coronary artery disease:*** | | | | | | | | | | | | | | | | | | |
| Aspirin | 984,807 | 42.9 | 62.4 | 183,176 | 38.7 | 57.2 | 205,945 | 42.6 | 61.4 | 197,094 | 44.7 | 64.3 | 196,263 | 42.9 | 63.4 | 202,329 | 45.0 | 65.3 |
| Statins | 984,807 | 23.9 | 66.2 | 183,176 | 25.4 | 63.4 | 205,945 | 24.2 | 65.4 | 197,094 | 23.7 | 66.5 | 196,263 | 23.0 | 66.3 | 202,329 | 23.3 | 69.2 |
| ACE I/ARB | 984,807 | 19.8 | 45.7 | 183,176 | 19.9 | 45.1 | 205,945 | 19.0 | 45.5 | 197,094 | 20.5 | 45.8 | 196,263 | 20.1 | 45.5 | 202,329 | 19.7 | 46.5 |
| CABG surgery (last 5 years) | 984,807 | 8.7 | 9.6 | 183,176 | 8.8 | 9.8 | 205,495 | 8.7 | 9.7 | 197,094 | 9.6 | 10.3 | 196,263 | 8.7 | 9.7 | 202,329 | 7.7 | 8.8 |
| ***Heart failure in patients requiring hospitalisation:*** | | | | | | | | | | | | | | | | | | |
| ACE I/ARB | 24,624 | 53.2 | 59.1 | 3,933 | 51.8 | 57.6 | 4,719 | 52.1 | 57.9 | 5,020 | 52.7 | 58.6 | 5,332 | 53.4 | 59.4 | 5,621 | 55.2 | 61.4 |
| B-Blocker | 24,624 | 25.4 | 28.2 | 3,933 | 24.3 | 27.0 | 4,719 | 24.5 | 27.2 | 5,020 | 25.0 | 27.8 | 5,332 | 25.6 | 28.5 | 5,621 | 27.1 | 30.1 |
| Spironolactone | 24,624 | 20.7 | 22.9 | 3,933 | 19.8 | 22.0 | 4,719 | 20.0 | 22.3 | 5,020 | 20.4 | 22.7 | 5,332 | 20.8 | 23.1 | 5,621 | 21.8 | 24.3 |
| Aspirin | 24,624 | 59.2 | 73.9 | 3,933 | 56.6 | 71.9 | 4,719 | 59.8 | 73.3 | 5,020 | 58.6 | 74.1 | 5,332 | 58.1 | 75.3 | 5,621 | 62.2 | 74.4 |
| ***Heart failure in the community:*** | | | | | | | | | | | | | | | | | | |
| ACE I/ARB | 172,770 | 45.6 | 68.9 | 28,063 | 48.2 | 70.2 | 35,493 | 44.5 | 69.3 | 36,590 | 43.4 | 67.8 | 35,571 | 45.9 | 69.2 | 37,053 | 46.6 | 68.4 |
| B-Blocker | 172,770 | 10.4 | 34.2 | 28,063 | 10.7 | 35.1 | 35,493 | 11.2 | 34.6 | 36,590 | 10.8 | 34.9 | 35,571 | 9.4 | 34.2 | 37,053 | 10.1 | 32.4 |
| Spironolactone | 172,770 | 3.9 | 14.5 | 28,063 | 4.3 | 14.7 | 35,493 | 3.9 | 14.9 | 36,590 | 3.6 | 13.0 | 35,571 | 3.9 | 15.1 | 37,053 | 4.0 | 14.9 |
| Aspirin | 172,770 | 38.1 | 50.4 | 28,063 | 37.9 | 46.3 | 35,493 | 38.3 | 49.9 | 36,590 | 37.3 | 50.3 | 35,571 | 37.0 | 51.8 | 37,053 | 40.0 | 52.7 |
| ***Numbers with CHD*** | 1,971,713 |  |  | 354,337 |  |  | 406,337 |  |  | 400,028 |  |  | 398,248 |  |  | 412,763 |  |  |
|  | | | | | | | | | | | | | | | | | | |
| ***Primary prevention therapies:*** | | | | | | | | | | | | | | | | | | |
| Anti-hypertension | 35,280,843 | 8.3 | 13.5 | 7,328,217 | 8.3 | 14.0 | 7,362,561 | 8.2 | 13.8 | 7,232,779 | 8.6 | 13.9 | 6,905,987 | 8.2 | 13.0 | 6,451,299 | 8.3 | 12.7 |
| Statins | 35,280,843 | 1.1 | 9.0 | 7,328,217 | 1.0 | 7.9 | 7,362,561 | 1.1 | 8.5 | 7,232,779 | 1.1 | 9.1 | 6,905,987 | 1.4 | 10.3 | 6,451,299 | 1.3 | 9.1 |
|  |  |  |  |  |  |  |  |  |  |  |  |  |  |  |  |  |  |  |

† For sources see Table B

†† We assumed no change in community-based CPR between 2000 and 2007

# Table I. Beta coefficients for major risk factors

**Estimated β coefficients from multiple regression analyses for the relationship between absolute changes in population mean risk factors and percentage changes in coronary heart disease mortality for men and women, stratified by age. Data sources, values and comments.**

| **Systolic blood pressure** | | **Age group (years)** | | | | |
| --- | --- | --- | --- | --- | --- | --- |
|  | | **25-44** | **45-54** | **55-64** | **65-74** | **75+** |
|  | |  |  |  |  |  |
| **Men** (hazard ratio per 20 mmHg) | | 0.49 | 0.49 | 0.52 | 0.58 | 0.65 |
| Men (log hazard ratio per 1 mmHg) | | **-0.036** | **-0.035** | **-0.032** | **-0.027** | **-0.021** |
|  | |  |  |  |  |  |
| *Minimum* | | *-0.029* | *-0.028* | *-0.026* | *-0.022* | *-0.017* |
| *Maximum* | | *-0.043* | *-0.042* | *-0.039* | *-0.032* | *-0.025* |
|  | |  |  |  |  |  |
|  | |  |  |  |  |  |
| **Women** (hazard ratio per 20 mmHg) | | 0.40 | 0.40 | 0.49 | 0.52 | 0.59 |
| Women (log hazard ratio per 1 mmHg) | | **-0.046** | **-0.046** | **-0.035** | **-0.032** | **-0.026** |
| *Minimum* | | *-0.037* | *-0.037* | *-0.028* | *-0.026* | *-0.021* |
| *Maximum* | | *-0.055* | *-0.055* | *-0.042* | *-0.039* | *-0.031* |
|  | |  |  |  |  |  |
|  | |  |  |  |  |  |
| Source: Prospective studies collaborative meta-analysis, Lancet 2002 [53] | | | | | | |
| Units: Percentage change in CHD mortality per 20 mmHg change in systolic blood pressure | | | | | | |
| **Strengths:** | Large dataset, includes US data, adjusted for regression dilution bias, consistent with randomised controlled trials, results stratified by age and sex, with 95% confidence intervals | | | | | |
| **Limitations:** | Some publication bias still possible | | | | | |

| **Cholesterol** | **Age groups (years)** | | | | | | |
| --- | --- | --- | --- | --- | --- | --- | --- |
|  | **25-44** | | **45-54** | **55-64** | **65-74** | **75-84** | **85+** |
| **Mortality reduction per 1 mmol/l** | | | | | | | |
| Men | 0.55 | | 0.53 | 0.36 | 0.21 | 0.21 | 0.21 |
| Women | 0.57 | | 0.52 | 0.35 | 0.23 | 0.23 | 0.23 |
| **Log coefficient** | | | | | | | |
| **Men** | **-0.799** | | **-0.755** | **-0.446** | **-0.236** | **-0.117** | **-0.083** |
| *Minimum* | *-0.639* | | *-0.604* | *-0.357* | *-0.189* | *-0.093* | *-0.067* |
| *Maximum* | *-0.958* | | *-0.906* | *-0.536* | *-0.283* | *-0.140* | *-0.100* |
|  |  | |  |  |  |  |  |
| **Women** | **-0.844** | | **-0.734** | **-0.431** | **-0.261** | **-0.174** | **-0.051** |
| *Minimum* | *-0.675* | | *-0.587* | *-0.345* | *-0.209* | *-0.139* | *-0.041* |
| *Maximum* | *-1.013* | | *-0.881* | *-0.517* | *-0.314* | *-0.209* | *-0.062* |
| Source: Prospective studies collaborative meta-analysis, Lancet 2007 [54] | | | | | | | |
| Units: | | Percentage change in CHD mortality per 1 mmol/l change in total cholesterol | | | | | |
| **Strengths:** | | Includes US data, adjusted for regression dilution bias, includes randomised controlled trials, RCT values consistent with observational data, results stratified by age and sex, with 95% confidence intervals | | | | | |
| **Limitations:** | | Some publication bias still possible | | | | | |

| **Body Mass Index (BMI)** | | **Age groups (years)** | | | | |
| --- | --- | --- | --- | --- | --- | --- |
|  | | **<44** | **45-59** | **60-69** | **70-79** | **80+** |
| *James et.al (2004):* | |  |  |  |  |  |
| Hazard ratio | | 0.89 | 0.91 | 0.95 | 0.96 | 0.97 |
| Risk reduction† per 1 kg/m^2^ | | 0.11 | 0.09 | 0.05 | 0.04 | 0.03 |
| Age gradient (45-59 as reference) | | 1.22 | **1.00** | 0.56 | 0.44 | 0.33 |
| *Bogers (2006):*  Relative risks, CHD deaths per 5 BMI units (kg/m^2^) | |  | **1.16** |  |  |  |
| Relative risks per 1 kg/m^2^ applying age gradients from James et.al | | 1.04 | 1.03 | 1.02 | 1.01 | 1.01 |
| **Log coefficients** | | **0.0363** | **0.0297** | **0.0165** | **0.0132** | **0.0099** |
| *Minimum* | | *0.0255* | *0.0209* | *0.0116* | *0.0093* | *0.0070* |
| *Maximum* | | *0.0466* | *0.0381* | *0.0212* | *0.0169* | *0.0127* |
| Source: Bogers et al (2006) [55], James et al (2004) [56] | | | | | | |
| Units: | Percentage change in CHD mortality per 1 kg/m^2^ change in BMI | | | | | |
| **Strengths:** | Large number of studies included. Adjusted for blood pressure, total cholesterol, and physical activity. 95% confidence intervals included. | | | | | |
| **Limitations:** | Observational data; age gradient applied from James study | | | | | |

^†^ Risk reduction = 1 – hazard ratio

# Table J. Relative Risks for CHD used in the IMPACT_SEC_ model for Smoking, Diabetes and Physical Inactivity

**Calculation of Relative Risk estimates for dichotomous risk factors in the IMPACT_SEC_ model**

Relative risks (RRs) estimated by expert working groups for the World Health Organization’s (WHO) Global Burden of Disease (GBD) 2001 Study were used for smoking and physical activity [9]. Effect estimates were based on systematic reviews of cohort studies (adjusted for regression dilution bias) and meta-analyses of randomised controlled trials. Age-variation in the relative risks for diabetes were taken from the DECODE study [10]. These were then applied to the sex-variation in relative risks estimated by Huxley et al [11]. The set of RRs used in the IMPACT_SEC_ model for the three binary risk factors with 95% Confidence Intervals (in parentheses) are shown below. RRs were assumed constant across deprivation quintiles.

|  | **Smoking** | **Physical inactivity** | **Diabetes** |
| --- | --- | --- | --- |
| **Male 25-34** | 5.51 (2.47-12.25) | 1.50 (1.35-1.67) | 4.33 (3.47-5.20) |
| **Male 35-44** | 5.51 (2.47-12.25) | 1.50 (1.35-1.67) | 3.22 (2.58-3.86) |
| **Male 45-54** | 3.04 (2.66-3.48) | 1.50 (1.35-1.67) | 2.14 (1.71-2.57) |
| **Male 55-64** | 2.51 (2.22-2.84) | 1.50 (1.35-1.67) | 1.99 (1.59-2.39) |
| **Male 65-74** | 1.69 (1.52-1.89) | 1.44 (1.30-1.61) | 1.86 (1.49-2.23) |
| **Male 75-84** | 1.31 (1.11-1.56) | 1.32 (1.19-1.47) | 1.71 (1.37-2.05) |
| **Male 85+** | 1.05 (0.78-1.43) | 1.23 (1.11-1.37) | 1.71 (1.37-2.05) |
| **Female 25-34** | 2.26 (0.83-6.14) | 1.50 (1.35-1.68) | 7.55 (6.04-9.06) |
| **Female 35-44** | 2.26 (0.83-6.14) | 1.50 (1.35-1.68) | 5.63 (4.51-6.76) |
| **Female 45-54** | 3.78 (3.10-4.62) | 1.50 (1.35-1.68) | 3.81 (3.05-4.57) |
| **Female 55-64** | 3.21 (2.70-3.82) | 1.50 (1.35-1.68) | 3.12 (2.50-3.74) |
| **Female 65-74** | 2.17 (1.89-2.47) | 1.45 (1.30-1.61) | 2.55 (2.04-3.06) |
| **Female 75-84** | 1.58 (1.33-1.88) | 1.33 (1.20-1.47) | 2.36 (1.89-2.83) |
| **Female 85+** | 1.38 (1.08-1.77) | 1.24 (1.13-1.37) | 2.36 (1.89-2.83) |

In Section J.1 we list the published RRs for each of the three risk factors; in Section J.2 we detail how these were modified to fit to the age-sex distributions used in the IMPACT_SEC_ model.

**J.1** Published relative risks

1 Current smoking

Relative risk of mortality from Ischaemic Heart Disease (ICD9: 410-414) for current smokers relative to non-smokers (95% CIs in parentheses), from the American Cancer Society’s Cancer Prevention Study (CPS-II)

| Age | Male | Female |
| --- | --- | --- |
| 30-44 | 5.51 (2.47-12.25) | 2.26 (0.83-6.14) |
| 45-59 | 3.04 (2.66-3.48) | 3.78 (3.10-4.62) |
| 60-69 | 1.88 (1.70-2.08) | 2.53 (2.22-2.87) |
| 70-79 | 1.44 (1.27-1.63) | 1.68 (1.46-1.93) |
| ≥ 80 years | 1.05 (0.78-1.43) | 1.38 (1.08-1.77) |

*Notes:* CPS-II is an ongoing prospective study of mortality in 1.2 million Americans aged 30 years or more when they completed a questionnaire on tobacco and alcohol use, diet, and multiple other factors affecting health and mortality in 1982. RRs were estimated from Cox proportional-hazard models, with non-smokers as the reference group (RR=1.0 for non-smokers). Risks were adjusted for age, race, education, marital status, “blue collar” employment in most recent or current job, weekly consumption of vegetables and citrus fruit, vitamin (A, C, and E) use, alcohol use, aspirin use, body mass index, exercise, dietary fat consumption and for hypertension and diabetes (both at baseline). Analyses of the hazards associated with smoking were based on the first six years of follow-up (1982 through 1988).

*Source: Ezzati et al (2005) [57]*

2 Physical inactivity

Relative risk of Ischaemic Heart Disease (ICD10: I20-I25) from physical (in)activity levels from WHO GBD Study (95% CIs in parentheses), relative to those considered physically active

| Age | Inactive level | Insufficiently active level |
| --- | --- | --- |
| 15-69 | 1.71 (1.58-1.85) | 1.44 (1.28-1.62) |
| 70-79 | 1.50 (1.38-1.61) | 1.31 (1.17-1.48) |
| 80+ years | 1.30 (1.21-1.41) | 1.20 (1.07-1.35) |

*Notes*: Physical (in)activity in the WHO GBD study was treated as a categorical variable with three categories: Level 1: Inactive: ‘doing no or very little physical activity at work, at home, for transport, or during discretionary time’. Level 2: Insufficiently active: ‘doing some physical activity but less than 150 minutes of moderate-intensity physical activity or 60 minutes of vigorous-intensity physical activity a week accumulated across work, home, transport or discretionary domains’. Level 3: Sufficiently active (unexposed): ‘at least 150 minutes of moderate-intensity physical activity or 60 minutes of vigorous-intensity physical activity a week accumulated across work, home, transport or discretionary domains’, which approximately corresponds to current recommendations in many countries. RR estimates were adjusted for confounding variables, measurement error associated with self-report, and attenuated over age (25% of the excess risk for the 70-79 year age-group and 50% of the excess risk for the oldest age group, 80+), but not adjusted for blood pressure and cholesterol.

*Sources: Bull et al (2004) [58]; Joubert et al (2007) [59]*

3 Diabetes

A meta-analysis of 22 prospective cohort studies by Huxley et al [11] estimated that the relative risk for CHD due to diabetes was 1.99 (95% CI: 1.69-2.35) in men and 3.12 (2.34-4.17) in women. These estimates were derived from studies that provided multiple risk factor adjusted coefficients. This systematic review included Asia-Pacific studies with larger RR values compared to Western studies, although the difference was not statistically significant. To obtain age-specific relative risk estimates, we used the age-gradients in relative risk for total mortality for diabetic persons compared to non-diabetics taken from the DECODE study as detailed below.

Estimates of relative risk for total mortality due to diabetes (DECODE study)

| Age | Males | Females |
| --- | --- | --- |
| 20-29 | 3.66 | 6.05 |
| 30-39 | 3.38 | 5.41 |
| 40-49 | 1.85 | 3.14 |
| 50-59 | 1.63 | 2.64 |
| 60-69 | 1.60 | 2.04 |
| 70-79 | 1.39 | 1.79 |

*Notes*: Undertaken in 1997, the Diabetes Epidemiology: Collaborative Analysis of Diagnostic Criteria in Europe (DECODE) study built a dataset that included the baseline values needed to determine the presence of the metabolic syndrome using a modification of the WHO definition for 11 European study cohorts, and follow-up data on all-cause and cardiovascular disease mortality [60].

*Source*: Roglic and Unwin (2010) [10]

**J.2** Adjusting the published RR values

The published relative risk values outlined in the previous section were adjusted to conform to the age distributions and binary classification of risk used in the IMPACT_SEC_ study. Table J above shows the final, adjusted RR values used in our model to estimate the Population Attributable Risk Fractions. Below we detail how the adjustments to the published RR values were calculated.

*Weighted averages using the European Standard Population*

We adjusted the RRs for each binary risk factor to match the ten-year age-bands used in our study. A population-weighted approach, using weights from the European Union (EU) reference population, was used to estimate the RRs for each of the 7 age-bands. We used the EU standard reference population for two reasons: first, for consistency. The EU standard was used as the reference population distribution in all IMPACT_SEC_ and related studies to calculate directly-standardised rates. Secondly, using the EU reference population aids comparability. The ensuing age-weighted rates can easily be used in studies in other European countries with a similar population structure and results compared against each other or with other health statistics (e.g. mortality rates) standardised using the same reference population.

1 Adjustment to published RRs for current smoking

The population-weighted adjustment approach is illustrated below using the RRs for current smoking in men as an example. For example, the RR value used in our model for males aged 55-64 was 2.51. This is roughly, but not exactly, halfway between the CPS-II estimates of 3.04 and 1.88 for males aged 55-59 and 60-64 with population weights of 0.545 and 0.455, respectively. The same calculations using a population weighted approach were performed using the CPS-II 95% confidence intervals to estimate the standard error of the RRs to use as input to the Ersatz Relative Risk function (See Table M).

| Age bands for IMPACT_SEC_ | 5 year age-bands | EU population | EU population weight | CPS-II RR | IMPACT_SEC_ RR |
| --- | --- | --- | --- | --- | --- |
| M 25-34 | M 25-29 | 7000 | 0.5 | 5.51 | 5.51 |
|  | M 30-34 | 7000 | 0.5 | 5.51 |  |
| M 35-44 | M 35-39 | 7000 | 0.5 | 5.51 | 5.51 |
|  | M 40-44 | 7000 | 0.5 | 5.51 |  |
| M 45-54 | M 45-49 | 7000 | 0.5 | 3.04 | 3.04 |
|  | M 50-54 | 7000 | 0.5 | 3.04 |  |
| M 55-64 | M 55-59 | 6000 | 0.545 | 3.04 | 2.51 |
|  | M 60-64 | 5000 | 0.455 | 1.88 |  |
| M 65-74 | M 65-69 | 4000 | 0.571 | 1.88 | 1.69 |
|  | M 70-74 | 3000 | 0.429 | 1.44 |  |
| M 75-84 | M 75-79 | 2000 | 0.667 | 1.44 | 1.31 |
|  | M 80-84 | 1000 | 0.333 | 1.05 |  |
| M 85+ | | 1000 | 1 | 1.05 | 1.05 |

2 Adjustment to published RRs for physical activity

We adjusted the published RRs for physical activity to employ a dichotomous rather than trichotomous measure (i.e. combining the GBD ‘insufficiently active’ and ‘inactive’ categories into a single inactive group). We used a weighted average approach using as weights the GBD estimates of exposure to physical inactivity in the EUR-A subregion (Table 10.10 in Bull et al, 2004) [58]. The physical activity exposure levels (%) with the corresponding RR value (RR=1 for the Level 3 ‘sufficiently active’ group) shown in parentheses are detailed below.

Physical activity exposure levels (%) in the GBD EUR-A region^†^ with accompanying RRs

| Exposure category | Age-group (years) | | | | | |
| --- | --- | --- | --- | --- | --- | --- |
|  | 15-29 | 30-44 | 45-59 | 60-69 | 70-79 | ≥ 80 years |
| Men: |  |  |  |  |  |  |
| Level 3: Recommended | 35 (1) | 29 (1) | 30 (1) | 30 (1) | 30 (1) | 32 (1) |
| Level 2: Insufficient | 52 (1.44) | 57 (1.44) | 55 (1.44) | 52 (1.44) | 50 (1.31) | 47 (1.20) |
| Level 1: Inactive | 13 (1.71) | 15 (1.71) | 16 (1.71) | 18 (1.71) | 20 (1.50) | 21 (1.30) |
| Levels 1 and 2  (combined RR) | *1.49* | *1.50* | *1.50* | *1.51* | *1.36* | *1.23* |
|  |  |  |  |  |  |  |
| Women: |  |  |  |  |  |  |
| Level 3: Recommended | 37 (1) | 31 (1) | 31 (1) | 33 (1) | 31 (1) | 30 (1) |
| Level 2: Insufficient | 47 (1.44) | 51 (1.44) | 51 (1.44) | 45 (1.44) | 45 (1.31) | 42 (1.20) |
| Level 1: Inactive | 17 (1.71) | 18 (1.71) | 18 (1.71) | 22 (1.71) | 24 (1.50) | 28 (1.30) |
| Levels 1 and 2  (combined RR) | *1.51* | *1.51* | *1.51* | *1.53* | *1.38* | *1.24* |

*Notes: For example, using the GBD estimates of exposure and RRs, the combined RR for males aged 45-59 equalled 1.50:*

RR (combining insufficient and inactive): ((0.55×1.44)+(0.16×1.71))/(0.55+0.16) = 1.50

^†^ Countries in the EUR-A GBD subregion: Andorra, Austria, Belgium, Croatia, Czech Republic, Denmark, Finland, France, Germany, Greece, Iceland, Ireland, Israel, Italy, Luxembourg, Malta, Monaco, Netherlands, Norway, Portugal, San Marino, Slovenia, Spain, Sweden, Switzerland, United Kingdom

*Source: Bull et al (2004) [58]*

Due to negligible differences in the combined RRs across the youngest age categories we used a relative risk of 1.50 for men and women aged 25-69. Having used the GBD data to obtain a set of RRs for a binary physical activity variable in each of three broad age-groups (25-69, 70-79, 80+) we then used the population-weighted average approach, using weights from the EU population, to estimate the combined RRs for the seven ten-year age-bands used in IMPACT_SEC_. This is illustrated below for males. The same sets of calculations using a population weighted approach were performed using the GBD 95% confidence intervals to estimate the standard error of the RRs to use as input into the Ersatz Relative Risk function (See Table M).

| Age bands for IMPACT_SEC_ | 5 year age-bands | EU population | EU population weight | Combined inactive RR | IMPACT_SEC_ RR |
| --- | --- | --- | --- | --- | --- |
| M 25-34 | M 25-29 | 7000 | 0.5 | 1.50 | 1.50 |
|  | M 30-34 | 7000 | 0.5 | 1.50 |  |
| M 35-44 | M 35-39 | 7000 | 0.5 | 1.50 | 1.50 |
|  | M 40-44 | 7000 | 0.5 | 1.50 |  |
| M 45-54 | M 45-49 | 7000 | 0.5 | 1.50 | 1.50 |
|  | M 50-54 | 7000 | 0.5 | 1.50 |  |
| M 55-64 | M 55-59 | 6000 | 0.545 | 1.50 | 1.50 |
|  | M 60-64 | 5000 | 0.455 | 1.50 |  |
| M 65-74 | M 65-69 | 4000 | 0.571 | 1.50 | 1.44 |
|  | M 70-74 | 3000 | 0.429 | 1.36 |  |
| M 75-84 | M 75-79 | 2000 | 0.667 | 1.36 | 1.32 |
|  | M 80-84 | 1000 | 0.333 | 1.23 |  |
| M 85+ | | 1000 | 1 | 1.23 | 1.23 |

3 Adjustment to published RRs for diabetes

Unlike the relative risk estimates for smoking and physical activity, the meta-analysis of 22 prospective cohort studies by Huxley et al [11] provided just overall estimates of RR for diabetes by gender. From previous studies we know that the relative risks associated with diabetes are higher for women and decline with age. To obtain age variation in the diabetes RRs, we used the age-gradient in the RR estimates for total mortality taken from the DECODE study [10]. As diabetes is a proximate risk factor for cardiovascular disease and cardiovascular disease comprises about half of total mortality [61], we have assumed that the relative age pattern of diabetes-related CHD mortality will be similar. We made the reasonable assumption that the mean age across the prospective studies examined in the meta-analysis by Huxley et al [11] was age 55-64 years. Hence the age-adjusted values were anchored to this age group for both sexes.

To estimate the relative risks for diabetes, we first used the DECODE study estimates to compute the age gradient in RRs, indexed on the value for the 55-64 age-group. The resulting value for each age group was then multiplied by the overall RR for men and women (1.99 and 3.12, respectively) taken from the study by Huxley et al [11] to give the age-specific RR of CHD mortality for diabetes. For ages 80 and over (for which published RRs were not found), we have assumed that the RR remained the same as for those aged 70-79. Detailed below is the worked example for males. Estimates of the 95% confidence intervals were not provided for the RRs taken from the DECODE study [10]. Hence, similar to previous IMPACT models, we used ± 20% of the point estimate as an approximation of the 95% confidence interval. We then used this interval to derive the standard error of the RR to use as an input parameter in the Ersatz uncertainty analysis. This value was equal to 0.103 - a value lying reasonably close to the average of the standard errors around the sex-specific estimates from Huxley et al [11].

| Age-bands for IMPACT_SEC_ | 5 year age bands | EU pop | EU pop weight | RR from DECODE study | Weighted RRs from DECODE | Age variation from DECODE^†^ | IMPACT_SEC_ RRs^‡^ |
| --- | --- | --- | --- | --- | --- | --- | --- |
| M 25-34 | M 25-29 | 7000 | 0.5 | 3.66 | 3.5 | 2.18 | 4.33 |
|  | M 30-34 | 7000 | 0.5 | 3.38 |  |  |  |
| M 35-44 | M 35-39 | 7000 | 0.5 | 3.38 | 2.6 | 1.62 | 3.22 |
|  | M 40-44 | 7000 | 0.5 | 1.85 |  |  |  |
| M 45-54 | M 45-49 | 7000 | 0.5 | 1.85 | 1.7 | 1.08 | 2.14 |
|  | M 50-54 | 7000 | 0.5 | 1.63 |  |  |  |
| M 55-64 | M 55-59 | 6000 | 0.545 | 1.63 | 1.6 | 1.00 | 1.99 |
|  | M 60-64 | 5000 | 0.455 | 1.60 |  |  |  |
| M 65-74 | M 65-69 | 4000 | 0.571 | 1.60 | 1.5 | 0.93 | 1.86 |
|  | M 70-74 | 3000 | 0.429 | 1.39 |  |  |  |
| M 75-84 | M 75-79 | 2000 | 0.667 | 1.39 | 1.4 | 0.86 | 1.71 |
|  | M 80-84 | 1000 | 0.333 | 1.39 |  |  |  |
| M 85+ |  | 1000 | 1 | 1.39 | 1.4 | 0.86 | 1.71 |

*Notes:*

^†^ 55-64 age-group taken as the reference

^‡^ IMPACT_SEC_ RR calculated as sex-specific RR from Huxley et al (2006) [11] (men:1.99; women:3.12) multiplied by age-variation in EU weighted RRs taken from DECODE study [10]

#

# *Table K. Risk factor levels in 2000 and 2007 by sex and deprivation quintiles*

|  | **England** | | **IMDQ1** | | **IMDQ2** | | **IMDQ3** | | **IMDQ4** | | **IMDQ5** | |
| --- | --- | --- | --- | --- | --- | --- | --- | --- | --- | --- | --- | --- |
|  | **2000** | **2007** | **2000** | **2007** | **2000** | **2007** | **2000** | **2007** | **2000** | **2007** | **2000** | **2007** |
| **Smoking prevalence (%)** |  |  |  |  |  |  |  |  |  |  |  |  |
| Male | 27.2 | 23.6 | 19.2 | 16.6 | 22.7 | 19.6 | 26.7 | 23.0 | 31.0 | 26.9 | 36.0 | 31.2 |
| Female | 23.4 | 19.9 | 17.2 | 14.7 | 20.0 | 17.0 | 22.9 | 19.5 | 26.2 | 22.2 | 29.9 | 25.2 |
| **Diabetes prevalence (%)** |  |  |  |  |  |  |  |  |  |  |  |  |
| Male | 3.7 | 6.5 | 3.3 | 5.7 | 3.5 | 6.1 | 3.7 | 6.5 | 3.6 | 6.1 | 4.5 | 8.1 |
| Female | 2.9 | 4.8 | 2.5 | 4.1 | 2.3 | 3.6 | 2.6 | 4.2 | 3.0 | 5.2 | 4.0 | 6.8 |
| **Physical inactivity (%)** |  |  |  |  |  |  |  |  |  |  |  |  |
| Male | 80.9 | 74.0 | 81.3 | 74.5 | 79.6 | 72.9 | 80.4 | 73.6 | 80.5 | 73.6 | 82.7 | 75.5 |
| Female | 82.4 | 78.1 | 82.3 | 78.0 | 82.5 | 78.3 | 81.6 | 77.4 | 81.9 | 77.7 | 83.9 | 79.5 |
| **Systolic blood pressure, mmHg** | | |  |  |  |  |  |  |  |  |  |  |
| Male | 133.1 | 130.6 | 133.1 | 130.5 | 133.4 | 130.8 | 133.3 | 130.7 | 133.0 | 130.6 | 133.0 | 130.6 |
| Female | 131.0 | 125.6 | 130.7 | 125.3 | 131.6 | 126.6 | 131.2 | 125.7 | 131.1 | 125.6 | 130.6 | 125.1 |
| **Cholesterol, mmol/L** |  |  |  |  |  |  |  |  |  |  |  |  |
| Male | 5.6 | 5.4 | 5.6 | 5.4 | 5.6 | 5.5 | 5.6 | 5.4 | 5.5 | 5.4 | 5.5 | 5.4 |
| Female | 5.7 | 5.5 | 5.7 | 5.6 | 5.8 | 5.6 | 5.7 | 5.5 | 5.6 | 5.4 | 5.6 | 5.5 |
| **Body mass index, kg/m^2^** |  |  |  |  |  |  |  |  |  |  |  |  |
| Male | 27.3 | 27.7 | 27.2 | 27.6 | 27.4 | 27.8 | 27.4 | 27.7 | 27.5 | 27.8 | 27.1 | 27.4 |
| Female | 26.9 | 27.2 | 26.3 | 26.5 | 26.7 | 26.9 | 27.0 | 27.2 | 27.2 | 27.5 | 27.6 | 27.9 |
| **Fruit and vegetable consumption, portions per day** | | |  |  |  |  |  |  |  |  |  |  |
| Male | 3.4 | 3.7 | 3.7 | 4.1 | 3.6 | 4.0 | 3.4 | 3.8 | 3.2 | 3.5 | 2.8 | 3.2 |
| Female | 3.6 | 4.0 | 4.0 | 4.4 | 3.8 | 4.3 | 3.6 | 4.0 | 3.4 | 3.8 | 3.0 | 3.3 |

|  |
| --- |

**Tables L. Model fit by age, sex and deprivation quintiles**

**Table L.1Total CHD deaths prevented or postponed (DPPs) by age, sex and deprivation quintiles (*expected deaths had 2000 rates persisted – observed deaths in 2007)***

|  | **England** | **IMDQ1** | **IMDQ2** | **IMDQ3** | **IMDQ4** | **IMDQ5** |
| --- | --- | --- | --- | --- | --- | --- |
| **Male** | 21972 | 4245 | 4727 | 4813 | 4375 | 3812 |
| **Female** | 16098 | 3108 | 3497 | 3536 | 3209 | 2747 |
| **Male 25-34** | 11 | -7 | 6 | 3 | 14 | -5 |
| **Male 35-44** | 157 | 39 | 40 | 10 | 9 | 59 |
| **Male 45-54** | 932 | 157 | 168 | 171 | 213 | 223 |
| **Male 55-64** | 2868 | 472 | 552 | 614 | 632 | 598 |
| **Male 65-74** | 6692 | 1169 | 1389 | 1480 | 1363 | 1292 |
| **Male 75-84** | 7773 | 1646 | 1666 | 1780 | 1471 | 1210 |
| **Male 85+** | 3539 | 770 | 907 | 755 | 673 | 434 |
|  |  |  |  |  |  |  |
| **Female 25-34** | 9 | 2 | -2 | 1 | 4 | 6 |
| **Female 35-44** | 44 | 7 | 8 | 22 | -11 | 17 |
| **Female 45-54** | 225 | 13 | 21 | 41 | 70 | 81 |
| **Female 55-64** | 977 | 128 | 182 | 147 | 208 | 312 |
| **Female 65-74** | 3281 | 483 | 646 | 748 | 704 | 700 |
| **Female 75-84** | 6402 | 1288 | 1307 | 1400 | 1341 | 1066 |
| **Female 85+** | 5158 | 1188 | 1335 | 1177 | 893 | 565 |
|  |  |  |  |  |  |  |
| **Total** | **38070** | **7353** | **8225** | **8349** | **7584** | **6558** |

**Table L.2 DPPs explained by model by age, sex and deprivation quintiles**

|  | **England** | **IMDQ1** | **IMDQ2** | **IMDQ3** | **IMDQ4** | **IMDQ5** |
| --- | --- | --- | --- | --- | --- | --- |
| **Male** | 15345 | 2782 | 3190 | 3241 | 3182 | 2950 |
| **Female** | 17425 | 2995 | 3556 | 3770 | 3688 | 3417 |
| **Male 25-34** | 24 | 1 | 2 | 1 | 8 | 10 |
| **Male 35-44** | 115 | 12 | 19 | 18 | 27 | 38 |
| **Male 45-54** | 364 | 58 | 65 | 63 | 79 | 98 |
| **Male 55-64** | 1864 | 275 | 351 | 370 | 395 | 474 |
| **Male 65-74** | 4213 | 709 | 861 | 907 | 878 | 859 |
| **Male 75-84** | 6224 | 1206 | 1317 | 1319 | 1277 | 1106 |
| **Male 85+** | 2541 | 520 | 575 | 564 | 518 | 365 |
|  |  |  |  |  |  |  |
| **Female 25-34** | 12 | 1 | 1 | 2 | 3 | 5 |
| **Female 35-44** | 33 | 4 | 4 | 6 | 6 | 12 |
| **Female 45-54** | 151 | 16 | 22 | 24 | 39 | 50 |
| **Female 55-64** | 573 | 69 | 101 | 114 | 128 | 161 |
| **Female 65-74** | 3133 | 435 | 568 | 639 | 679 | 812 |
| **Female 75-84** | 6469 | 1107 | 1342 | 1398 | 1405 | 1216 |
| **Female 85+** | 7054 | 1362 | 1517 | 1586 | 1428 | 1160 |
|  |  |  |  |  |  |  |
| **Total** | **32770** | **5777** | **6746** | **7011** | **6870** | **6367** |

Notes: DPPs explained after adjustment for poly-pharmacy and cumulative risk factor reduction

**Table L.3 Model fit^†^ by age, sex and deprivation quintiles**

|  | **England** | **IMDQ1** | **IMDQ2** | **IMDQ3** | **IMDQ4** | **IMDQ5** |
| --- | --- | --- | --- | --- | --- | --- |
| **Male** | 70% | 66% | 67% | 67% | 73% | 77% |
| **Female** | 108% | 96% | 102% | 107% | 115% | 124% |
|  |  |  |  |  |  |  |
| **Male 25-34** | 217% | 17% | 42% | 40% | 61% | 225% |
| **Male 35-44** | 73% | 32% | 47% | 185% | 285% | 65% |
| **Male 45-54** | 39% | 37% | 39% | 37% | 37% | 44% |
| **Male 55-64** | 65% | 58% | 64% | 60% | 63% | 79% |
| **Male 65-74** | 63% | 61% | 62% | 61% | 64% | 66% |
| **Male 75-84** | 80% | 73% | 79% | 74% | 87% | 91% |
| **Male 85+** | 72% | 68% | 63% | 75% | 77% | 84% |
|  |  |  |  |  |  |  |
| **Female 25-34** | 130% | 74% | 35% | 260% | 95% | 92% |
| **Female 35-44** | 75% | 63% | 53% | 29% | 52% | 70% |
| **Female 45-54** | 67% | 127% | 108% | 59% | 55% | 62% |
| **Female 55-64** | 59% | 54% | 55% | 78% | 62% | 52% |
| **Female 65-74** | 95% | 90% | 88% | 85% | 96% | 116% |
| **Female 75-84** | 101% | 86% | 103% | 100% | 105% | 114% |
| **Female 85+** | 137% | 115% | 114% | 135% | 160% | 205% |
|  |  |  |  |  |  |  |
| **Total** | **86%** | **79%** | **82%** | **84%** | **91%** | **97%** |

**^†^** Model fit = absolute % of the total DPPs explained by the model:

***% Model fit = ABSOLUTE (1- ((total DPPs – model DPPs)/total DPPs)) × 100***

**Table L.4: Overall model fit by deprivation quintiles: comparing modelled deaths prevented or postponed (DPPs) against observed fall in CHD deaths, and 95% uncertainty intervals (UI).**

|  | **Target DPPs** | **Explained DPPs** | ***Lower UI***  ***(95%)*** | ***Upper UI***  ***(95%)*** | **Explained (%)** | ***Lower UI (%)*** | ***Upper UI (%)*** |
| --- | --- | --- | --- | --- | --- | --- | --- |
| **IMDQ1** | 7353 | 5777 | *4134* | *7420* | 78.6 | *56.2* | *100.9* |
| **IMDQ2** | 8225 | 6746 | *4999* | *8492* | 82.0 | *60.8* | *103.3* |
| **IMDQ3** | 8349 | 7011 | *4872* | *9151* | 84.0 | *58.4* | *109.6* |
| **IMDQ4** | 7584 | 6870 | *5146* | *8595* | 90.6 | *67.8* | *113.3* |
| **IMDQ5** | 6558 | 6367 | *4887* | *7848* | 97.1 | *74.5* | *119.7* |
| **England** | **38070** | **32770** | ***24681*** | ***40861*** | **86.1** | ***64.8*** | ***107.3*** |

**Figure L.1: Model fit by age, gender and deprivation quintiles**


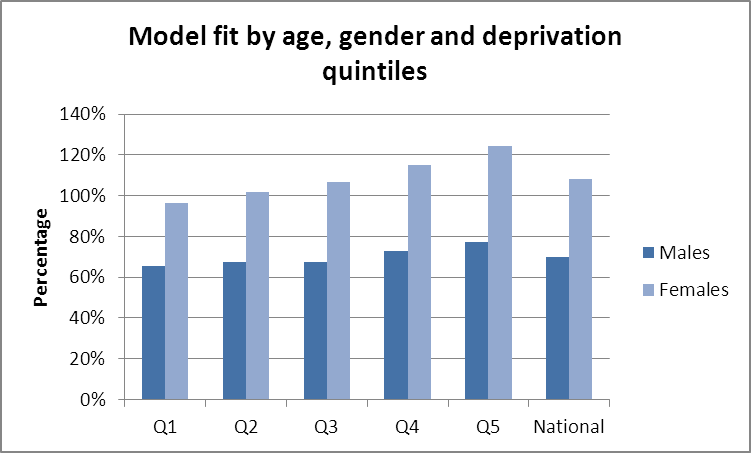


# Table M. Uncertainty analysis: parameter distributions, functions and sources

Table M records the type of distribution and associated functions for each of the input variables in the IMPACT_SEC_ model. We implemented stochastic uncertainty analysis in Excel using Ersatz (version 1.0 available at [http://www.epigear.com](http://www.epigear.com/)), an add-in that allows probabilistic bootstrapping in Excel [62]. Ersatz allows repeated random draws from specified distributions for input variables that are used to recalculate iteratively the model. It then calculates the 95% uncertainty intervals from the realised values of the output variable (deaths prevented or postponed). For the IMPACT_SEC_ model, we calculated the uncertainty intervals based on 1000 draws taking the 95% uncertainty intervals as the 2.5^th^ and 97.5^th^ percentiles. Input variables taken from external sources (e.g. case fatality rates, beta coefficients and relative risk reductions) were randomly drawn from specified distributions but assumed constant across deprivation quintiles. Worked examples using Ersatz to estimate uncertainty intervals for net treatment DPPs and DPPs attributable to risk factor change are shown below Table M.

| **Input parameters** | ***Type of distribution and functions (Mean, Standard error)*** | Source |
| --- | --- | --- |
| **Population** | | |
| Population counts and CHD deaths stratified by age, sex, and Index of Multiple Deprivation quintiles | - Population counts (no error) - Deaths expected in 2007 had CHD mortality rates in 2000 persisted (***Poisson distribution***) | Office for National Statistics |
| **Risk factors** | | |
| Prevalence/mean estimates (pooled data; national estimates for 2000 and 2007) | - Prevalence estimates (smoking, physical activity, diabetes): (***Beta distribution***: cases, sample-size minus cases) - Continuous variables (Body Mass Index, SBP, total cholesterol, fruit and vegetable consumption): (***Normal distribution***: mean, SE of mean) | Health Survey for England |
| RR: **smoking** | ***Ersatz RR function*** (RR, SE ln(RR)):  RRs and 95% CIs shown in Table S10. | Ezzati et al (2005) [57] |
| RR: **physical activity** | ***Ersatz RR function*** (RR, SE ln(RR)):  RRs and 95% CIs shown in Table S10. | Bull et al (2004) [58] |
| RR: **diabetes** | ***Ersatz RR function*** (RR, SE ln(RR)):  RRs and 95% CIs shown in Table S10. | Roglic and Unwin (2010) [10]; Huxley et al (2006) [11] |
| Beta coefficient: **Body Mass Index** | ***Normal distribution*** (mean, SE of mean):  M & F < 45 (0.036,0.005); M & F 45-54 (0.030, 0.004)  M & F 55-64 (0.023,0.003); M & F 65-74 (0.015, 0.002)  M & F 75-84 (0.012,0.002); M & F 85+ (0.010, 0.001) | Bogers et al (2006) [55], James et al (2004) [56]. Parameters on the log scale. |
| Beta coefficient: **SBP** | ***Normal distribution*** (mean, SE of mean):  M < 45 (-0.036,0.004); M 45-54 (-0.035,0.004)  M 55-64 (-0.032,0.003); M 65-74 (-0.027,0.003)  M 75-84 (-0.021,0.002); M 85+ (-0.016,0.002)  F < 55 (-0.046, 0.005); F 55-64 (-0.035,0.004)  F 65-74 (-0.032,0.003); F 75-84 (-0.026,0.003)  F 85+ (-0.019,0.002) | Prospective studies collaborative meta-analysis (2002) [53]. Parameters on the log scale. |
| Beta coefficient: **total cholesterol** | ***Normal distribution*** (mean, SE of mean):  M < 45 (-0.799,0.081); M 45-54 (-0.755,0.077)  M 55-64 (-0.446,0.046); M 65-74 (-0.236,0.024)  M 75-84 (-0.117,0.012); M 85+ (-0.083,0.009)  F < 45 (-0.844,0.086); F 45-54 (-0.734,0.075)  F 55-64 (-0.431,0.044); F 65-74 (-0.261,0.027)  F 75-84 (-0.174,0.018); F 85+ (-0.051,0.005) | Prospective studies collaborative meta-analysis (2007) [54]. Parameters on the log-scale. |
| Beta coefficient: **Fruit and vegetable consumption** | ***Normal distribution*** (mean, SE of mean):  M & F (-0.041,0.016) | Dauchet et al (2006) [7]. Parameters on the log-scale. |
| **ST elevation myocardial infarction (STEMI)** | | |
| **Eligible patients**:  Emergency admissions with a primary diagnosis of myocardial infarction (ratio of STEMI/nSTEMI as 40/60) | ***Poisson distribution*** (admissions) | Hospital Episode Statistics (HES) |
| **Case fatality rate** | Sample size (*n*) = STEMI admissions:  ***Beta distribution*** (cases = *n* × CFR estimate, non-cases = *n* – cases) | Wijeysundera et al (2010) [5] |
| **Treatment uptake** | - *Medications and in-hospital CPR:* ***Beta distribution*** (cases = STEMI admissions from MINAP × medication uptake, non-cases = STEMI admissions – cases) - *PCI and CABG*: ***Beta distribution*** (cases = MI admissions from HES × PCI/CABG uptake, non-cases = MI admissions – cases) | MINAP for treatment uptake (2003 and 2007 for start and end year respectively); HES for number of admissions (2000 and 2007 for start and end year respectively) |
| **Relative risk reduction:** | ***Ersatz RR function*** (RRR, SE ln(RRR)): |  |
| In-hospital CPR | M & F (33%,0.103): absolute risk reduction | Tunstall-Pedoe (1992) [33] |
| Thrombolysis  Aspirin  Beta-blockers  Primary PCI  Primary CABG surgery  ACE Inhibitors | M & F (0.31,0.298)  M & F (0.23,0.177)  M & F (0.04,0.691): assumed lower limit of 1%  M & F (0.30,0.587)  M & F (0.39,0.293)  M & F (0.07,0.435) | Estess (2002) [25]  ISIS-2 (1998) [26]  Freemantle (1999) [29]  Keeley (2003) [28]  Yusuf (1994) [27]  ACE-I MI Collaborative Group (1998) [30] |
| Clopidogrel | M & F (0.03,0.457) | Chen (2003) [31], Sabatine (2005) [32] |
| **Non-ST segment elevation acute coronary syndrome (NSTEACS)** | | |
| **Eligible patients**:  Emergency admissions with a primary diagnosis of myocardial infarction (ratio of STEMI/nSTEMI as 40/60) or primary diagnosis of unstable angina | ***Poisson distribution*** (nSTEMI + unstable angina admissions) | Hospital Episode Statistics (HES) |
| **Case fatality rate** | Sample size (*n*) = nSTEMI + unstable angina admissions:  ***Beta distribution*** (cases = *n* × CFR estimate, non-cases = *n* – cases) | Wijeysundera et al (2010) [5] |
| **Treatment uptake** | - *Medications and in-hospital CPR:* ***Beta distribution*** (cases = NSTEACS admissions × medication uptake, non-cases = NSTEACS admissions – cases) - *PCI and CABG*: ***Beta distribution*** (cases = unstable angina admissions from HES × PCI/CABG uptake, non-cases = unstable angina admissions – cases) | MINAP (2003 and 2007 for start and end year respectively); HES (2000 and 2007 for start and end year respectively) |
| **Relative risk reduction:** | ***Ersatz RR function*** (RRR, SE ln(RRR)): |  |
| In-hospital CPR | M & F (33%,0.103): absolute risk reduction | Tunstall-Pedoe (1992) [33] |
| Aspirin & heparin  Primary CABG surgery  Early PCI  Beta blockers  Clopidogrel  ACE Inhibitors | M & F (0.33,0.470)  M & F (0.39,0.293)  M & F (0.32,0.592)  M & F (0.04,0.691): assumed lower limit of 1%  M & F (0.07,0.435)  M & F (0.07,0.435) | Oler (1996) [36]  Yusuf (1994) [27]  RITA 3 (Fox 2005) [38]  Freemantle (1999) [29]  Yusuf (2001) [39]  ACE-I MI Collaborative Group (1998) [40] |
| Aspirin alone | M & F (0.15,0.139) | Antithrombotic Trialists’ Collaboration ATC (2002) [35] |
| Platelet glycoprotein IIB/IIIA inhibitors | M & F (0.09,0.530) | Boersma (2002) [37] |
| **Secondary prevention post myocardial infarction (MI)** | | |
| **Eligible patients**:  Ever having had a myocardial infarction (prior to 1/1/2007) | ***Poisson distribution*** (Population in 2007 × (post-MI prevalence obtained from GPRD) minus assumed overlap with Heart Failure) | General Practice Research Database (GPRD) |
| **Case fatality rate** | Sample size (*n*) = ever having had MI in GPRD in 2007:  ***Beta distribution*** (cases = *n* × CFR estimate, non-cases = *n* – cases) | Wijeysundera et al (2010) [5] |
| **Treatment uptake** | ***Beta distribution*** (cases = *n* × medication uptake, non-cases = *n* – cases) | GPRD (2000 and 2007 for start and end year respectively) |
| **Compliance** | Sample size (*n1*) = ever having had MI in GPRD in 2007 with record of medication use:  ***Beta distribution*** (cases = *n1* × assumed compliance, non-cases = *n1* – cases) |
| **Relative risk reduction:** | ***Ersatz RR function*** (RRR, SE ln(RRR)): |  |
| Aspirin  Beta blockers  ACE Inhibitors  Statins  Warfarin | M & F (0.15,0.139)  M & F (0.23,0.185)  M & F (0.20,0.177)  M & F (0.24,0.245)  M & F (0.22,0.305) | ATC (2002) [35]  Freemantle (1999) [29]  Flather (2000) [40]  Hulten (2006) [41]  Anand and Yusuf (1999) [42] |
| **Secondary prevention post revascularisation** | | |
| **Eligible patients**:  Ever having had a revascularisation procedure, and assumed be alive prior to 1/1/2007 | - ***Poisson distribution*** (CABG/PTCA procedures from 2000 to 2007 minus i) annual 5% mortality adjustment, ii) assumed overlap with post myocardial infarction group) - ***Rehabilitation (mortality benefits within last 5 years only): Poisson distribution*** (CABG/PTCA procedures from 2002 to 2007 minus i) annual 5% mortality adjustment, ii) assumed overlap with post myocardial infarction group) | Hospital Episode Statistics (HES) |
| **Case fatality rate** | Sample size (*n*) = ever having had post-revascularisation in GPRD in 2007:  ***Beta distribution*** (cases = *n* × CFR estimate, non-cases = *n* – cases) | Wijeysundera et al (2010) [5] |
| **Treatment uptake** | ***Beta distribution*** (cases = *n* × medication uptake, non-cases = *n* – cases) | GPRD (2000 and 2007 for start and end year respectively) |
| **Compliance** | Sample size (*n1*) = ever having had revascularisation in GPRD in 2007 with record of medication use:  ***Beta*** ***distribution*** (cases = *n1* × assumed compliance, non-cases = *n1* – cases) |
| **Relative risk reduction:** | ***Ersatz RR function*** (RRR, SE ln(RRR)): |  |
| Aspirin  Beta blockers  ACE Inhibitors  Statins  Rehabilitation  Warfarin | M & F (0.15,0.139)  M & F (0.23,0.185)  M & F (0.20,0.177)  M & F (0.24,0.245)  M & F (0.26,0.347)  M & F (0.22,0.305) | ATC (2002) [35]  Freemantle (1999) [29]  Flather (2000) [40]  Hulten (2006) [41]  Taylor (2004) [43]  Anand and Yusuf (1999) [42] |
| **Chronic stable coronary artery disease** | | |
| **Eligible patients**:  Ever having had chronic stable artery disease but no myocardial infarction (prior to 1/1/2007) | ***Poisson*** ***distribution*** (Population in 2007 × (angina but no myocardial infarction prevalence obtained from GPRD) minus i) emergency admissions for unstable angina and ii) assumed overlaps with heart failure and post-revascularisation groups) | General Practice Research Database (GPRD) |
| **Case fatality rate** | Sample size (*n*) = ever having had angina but no MI in GPRD in 2007:  ***Beta distribution*** (cases = *n* × CFR estimate, non-cases = *n* – cases) | Wijeysundera et al (2010) [5] |
| **Treatment uptake** | ***Beta distribution*** (cases = *n* × medication uptake, non-cases = *n* – cases) | GPRD (2000 and 2007 for start and end year respectively) |
| **Compliance** | Sample size (*n1*) = ever having had angina but no MI in GPRD in 2007 with record of medication use  ***Beta distribution*** (cases = *n1* × assumed compliance, non-cases = *n1* – cases) |
| **Relative risk reduction:** Statins  Aspirin  ACE Inhibitors | ***Ersatz RR function*** (RRR, SE ln(RRR)):  M & F (0.23,0.244)  M & F (0.15,0.139)  M & F (0.17,0.177) | Wilt (2004) [45]  ATC (2002) [35]  Al-Mallah (2006) [46] |
| **CABG for chronic stable coronary artery disease (0-5 years)** | | |
| **Eligible patients**:  Ever having had chronic stable artery disease but no myocardial infarction (prior to 1/1/2007) | ***Poisson distribution*** (estimated count of patients with stable coronary artery disease (described above) in 2007 × (estimated uptake of CABG)):   - ***2007***: Uptake of CABG: (number of CABG procedures from 2002 to 2007)/eligible patients in 2007 - ***2000***: Uptake of CABG: (number of CABG procedures from 1995 to 2000)/eligible patients in 2000 | Hospital Episode Statistics |
| **Case fatality rate** | As described in the post-revascularisation group |  |
| **Treatment uptake and compliance** | Fixed at 100% |  |
| **Relative risk reduction:** CABG (0-5 years) | ***Ersatz RR function*** (RRR, SE ln(RRR)):  M & F (0.39,0.293) | Yusuf (1994) [27] |
| **CABG for chronic stable coronary artery disease (6-10 years)** | | |
| **Eligible patients**:  Ever having had chronic stable artery disease but no myocardial infarction (prior to 1/1/2007) | ***Poisson distribution*** (estimated count of patients with stable coronary artery disease (described above) in 2007 × (estimated uptake of CABG)):   - ***2007***: Uptake of CABG: (number of CABG procedures from 2000 & 2001)/eligible patients in 2007 - ***2000***: Uptake of CABG: (number of CABG procedures from 1993 & 1994)/eligible patients in 2000 | Hospital Episode Statistics |
| **Case fatality rate** | As described in the post-revascularisation group |  |
| **Treatment uptake and compliance** | Fixed at 100% |  |
| **Relative risk reduction:** CABG (6-10 years) | ***Ersatz RR function*** (RRR, SE ln(RRR)):  M & F (0.32,0.243) | Yusuf (1994) [27] |
| **Heart failure in patients requiring hospitalisation** | | |
| **Eligible patients**:  Admissions with a primary diagnosis of heart failure | ***Poisson distribution*** (admissions minus 50% assumed not to be due to CHD) | Hospital Episode Statistics (HES) |
| **Case fatality rate** | Sample size (*n*) = HES admissions for HF divided by 2 as only half assumed to be CHD related  ***Beta distribution*** (cases = *n* × CFR estimate, non-cases = *n* – cases) | Wijeysundera et al (2010) [5] |
| **Treatment uptake** | - Aspirin: as described in the post-myocardial infarction group   Other medications: Sample size (*n1*) = HF admissions from NHS survey:   - ***Beta distribution*** (cases = *n1* × medication uptake, non-cases = *n1* – cases) | NHS Heart Failure survey (2005).  2005 rates taken as 2007 values. Rates assumed 10% lower in 2000. |
| **Compliance** | Sample size (*n2*) = HF admissions from NHS survey with record of medication use:  ***Beta distribution*** (cases = *n2* × assumed compliance, non-cases = *n2*  – cases) | NHS Heart Failure Survey |
| **Relative risk reduction:** Aspirin  ACE Inhibitors  Beta blockers  Spironolactone | ***Ersatz RR function*** (RRR, SE ln(RRR)):  M & F (0.15,0.139)  M & F (0.20,0.177)  M & F (0.35,0.128)  M & F (0.30,0.128) | ATC (2002) [35]  Flather (2000) [40]  Shibata (2001) [47]  Pitt (1999) [48] |
| **Heart failure in the community** | | |
| **Eligible patients**:  Ever having had heart failure (prior to 1/1/2007) | ***Poisson distribution*** (Population in 2007 × HF prevalence obtained from GPRD divided by 2 as only half assumed to be CHD related) minus HF hospital admissions) | General Practice Research Database, Hospital Episode Statistics |
| **Case fatality rate** | Sample size (*n*) = ever having had HF in GPRD in 2007:  ***Beta distribution*** (cases = *n* × CFR estimate, non-cases = *n* – cases) | Wijeysundera et al (2010) [5] |
| **Treatment uptake** | ***Beta distribution*** (cases = *n* × medication uptake, non-cases = *n* – cases) | GPRD (2000 and 2007 for start and end year respectively) |
| **Compliance** | Sample size (*n1*) = ever having had HF in GPRD in 2007 with record of medication use:  ***Beta distribution*** (cases = *n1* × assumed compliance, non-cases = *n1* – cases) |
| **Relative risk reduction:** Aspirin  ACE Inhibitors  Beta blockers  Spironolactone | ***Ersatz RR function*** (RRR, SE ln(RRR)):  M & F (0.15,0.139)  M & F (0.20,0.177)  M & F (0.35,0.128)  M & F (0.31,0.216) | ATC (2002) [35]  Flather (2000) [40]  Shibata (2001) [47]  Pitt (1999) [48] |
| **Primary prevention therapies: Statins** | | |
| **Eligible patients**:  Population | Population counts (no error) | Office for National Statistics |
| **Treatment uptake** | % never having had angina or heart attack and currently taking lipid lowering drugs prescribed by a doctor: (***Beta distribution***: cases, sample-size minus cases) | Health Survey for England |
| **Case fatality rate** | Sample size (*n*) = never having had angina or heart attack and currently taking lipid lowering drugs in 2006:  ***Beta distribution*** (cases = *n* × CFR estimate, non-cases = *n* – cases) | Wijeysundera et al (2010) [5] |
| **Compliance** | ***Beta distribution*** (cases = *n* × assumed compliance, non-cases = *n* – cases) | Health Survey for England |
| **Relative risk reduction:** Statins | ***Ersatz RR function*** (RRR, SE ln(RRR)):  M & F (0.35,0.396) | Pignone (2000) [52] |
| **Primary prevention therapies: Treatments for high blood pressure** | | |
| **Eligible patients**:  Population | Population counts (no error) | Office for National Statistics |
| **Treatment uptake** | % never having had angina or heart attack and currently taking medication specifically prescribed to treat high blood pressure: (***Beta distribution***: cases, sample-size minus cases) | Health Survey for England |
| **Case fatality rate** | Sample size (*n*) = never having had angina or heart attack and currently taking medication to lower blood pressure in 2006:  ***Beta distribution*** (cases = *n* × CFR estimate, non-cases = *n* – cases) | Wijeysundera et al (2010) [5] |
| **Compliance** | ***Beta distribution*** (cases = *n* × assumed compliance, non-cases = *n* – cases) | Health Survey for England |
| **Relative risk reduction:** Treatments for high blood pressure | ***Ersatz RR function*** (RRR, SE ln(RRR)):  M & F (0.13,0.294) | Law (2003) [51] |

**WORKED EXAMPLES USING ERSATZ FOR UNCERTAINTY ANALYSIS**

Below we illustrate our use of Ersatz for uncertainty analysis using males aged 75-84 in the most deprived quintile (~ means “distributed as”).

**1 Uncertainty analysis for treatments**

The net effects of aspirin in the secondary prevention post MI (within the last five years) group was calculated as follows:

***DPPs (2007) = patient numbers × treatment uptake_2007_ × compliance × relative mortality reduction × 1-year case fatality***

= (12 226) × 0.75 × 0.70 × 0.15 × 0.067 ≈ 65

***DPPs (2000) = patient numbers × treatment uptake_2000_ × compliance × relative mortality reduction × 1-year case fatality***

= (12 226) × 0.59 × 0.70 × 0.15 × 0.067 ≈ 51

Net DPPs were therefore calculated as = **DPPs_2007_ – DPPs_2000_** ≈ 65 – 51 ≈ 14

Table M above shows the probability distributions and associated Ersatz functions used for each input variable in the DPP calculations. For secondary prevention post MI we used Poisson (patient numbers); beta (treatment uptake, compliance, and case fatality rate) and the Ersatz RR function (relative risk reduction in the 1-year case fatality rate owing to treatment). More specifically, for males aged 75-84 in IMDQ5, the input values for the uncertainty analysis were as follows:

- Patient numbers ~ Poisson (population in 2007 × post-MI prevalence) minus overlap with the heart failure in the community group ~ Poisson (12 226)
- Treatment uptake in 2007 and 2000 ~ Beta (cases, non-cases)

If we let *n* denote ever having had MI in 2007 then cases = (*n* × uptake of aspirin) and non-cases = (*n* – cases). Cases = (587 × 0.75) = 440; non-cases = (587-440) = 147. Treatment uptake in 2007 was therefore ~ Beta (440,147). Likewise, treatment uptake in 2000 was ~ Beta (247,169).

- Compliance ~ Beta (cases, non-cases)

If we let *n1* denote ever having had MI in 2007 *and* with record of medication use in 2007 then cases = (*n1* × assumed compliance (0.70)) and non-cases = (*n1* – cases). Cases = (440 × 0.70) = 308; non-cases = (440-308) = 132. Compliance therefore ~ Beta (308,132)

- Relative risk reduction (RRR) ~ Ersatz RR function (RRR, SE ln(RRR))

where SE and ln denote standard error and natural logarithm, respectively. RRR was 1-odds ratio for aspirin use in the community taken from ATC (2002) = 1-0.85 = 0.15; with 95% Confidence Interval (0.11,0.19) [35]. Using the 95% CIs, the SE of ln(RR) was calculated as:

ln(0.19)-ln(0.11)/(1.96×2) = 0.139

Relative risk reduction for aspirin use was therefore ~ Ersatz RR function (0.15,0.139)

- Case fatality rate ~ Beta (cases, non-cases)

Parameter uncertainty around case fatality rates was calculated at the national level and assumed constant across all IMD quintiles. If we let *n* denote ever having had MI in 2007 in England then cases = (*n* × assumed CFR (0.067)) and non-cases = (*n* – cases). Using the GPRD data for males aged 75-84 and assumed CFRs [5], cases = (5348 × 0.067) = 358; non-cases = (5348-358) = 4990. Case fatality for the post-MI group was therefore ~ Beta (358, 4990).

Putting all this together, 10 runs in Ersatz gave the following estimates of each input variable randomly drawn from the relevant probability distributions from which we calculate the net DPPs for aspirin use in male post myocardial infarction survivors aged 75-84 in IMDQ5:

**Ten runs in Ersatz to calculate net DPPs for aspirin use in males aged 75-84 in IMDQ5 (post-MI in last 5 years)**

| **Run** | **Numbers** | **Uptake_2007_** | **Compliance** | **Relative risk reduction** | **Case fatality rate** | **Uptake_2000_** | **Net DPPs†** |
| --- | --- | --- | --- | --- | --- | --- | --- |
|  | **Probability distributions** | | | | | | |
|  | **Poisson** | **Beta** | **Beta** | **RR** | **Beta** | **Beta** |  |
|  | **Col A** | **Col B** | **Col C** | **Col D** | **Col E** | **Col F** |  |
| 1 | 12 272 | 0.737 | 0.681 | 0.166 | 0.068 | 0.591 | 13.7 |
| 2 | 12 297 | 0.759 | 0.653 | 0.184 | 0.069 | 0.581 | 18.3 |
| 3 | 12 225 | 0.741 | 0.687 | 0.145 | 0.076 | 0.607 | 12.3 |
| 4 | 12 428 | 0.736 | 0.697 | 0.166 | 0.069 | 0.587 | 14.6 |
| 5 | 12 252 | 0.712 | 0.778 | 0.189 | 0.065 | 0.580 | 15.5 |
| 6 | 12 141 | 0.782 | 0.727 | 0.175 | 0.068 | 0.588 | 20.4 |
| 7 | 12 154 | 0.777 | 0.715 | 0.135 | 0.066 | 0.592 | 14.4 |
| 8 | 12 397 | 0.734 | 0.698 | 0.151 | 0.069 | 0.591 | 12.9 |
| 9 | 12 220 | 0.723 | 0.727 | 0.153 | 0.070 | 0.558 | 15.6 |
| 10 | 12 427 | 0.772 | 0.684 | 0.186 | 0.065 | 0.593 | 18.5 |
| **Point estimate** | **12 226** | **0.75** | **0.70** | **0.15** | **0.067** | **0.59** | **14** |

^†^ Net DPPs = (A×B×C×D×E) - (A×F×C×D×E)

*Uncertainty intervals for treatment contribution DPPs*

In each Ersatz run, net DPPs were calculated for all age-sex-IMD groups and were then summed for each medication within each of the 9 mutually exclusive CHD patient groups. Each of the nine treatment DPP totals was then multiplied by a correction for poly-pharmacy (which varied across patient groups but took the same value in each of the 1000 Ersatz runs). An estimate of the total treatment contribution to model DPPs was obtained by summing the nine patient group totals.

95% uncertainty intervals from the set of 1000 runs (2.5^th^ and 97.5^th^ percentiles) were extracted for the nine treatment totals plus the overall estimate of treatment contribution DPPs.

**2 Uncertainty analysis for change in binary risk factors**

The DPPs attributable to change in smoking prevalence over 2000-2007 was calculated as follows:

***DPPs = expected CHD deaths in 2007 (had mortality rates in 2000 remained constant) × (PARF_2000_ – PARF_2007_)***

where PARF = [P × (RR-1)] / [1 + P × (RR-1)]; P is the prevalence of the risk factor and RR is the relative risk for CHD mortality associated with risk factor presence.

For the three binary risk factors (smoking, diabetes, and physical activity) we used the following probability distributions: Poisson (expected deaths); beta (risk factor prevalence) and the Ersatz RR function (relative risk reduction owing to elimination of exposure). More specifically, for males aged 75-84 in IMDQ5, the input values for the uncertainty analysis were as follows:

- Expected CHD deaths in 2007 ~ Poisson (population in 2007 × CHD mortality rates in 2000) ~ Poisson (4236)
- Estimates of smoking prevalence~ Beta (cases, non-cases)

If we let *n* denote the number of Health Survey for England male respondents aged 75-84 in IMDQ5 over all years from 2000 to 2007 (i.e. pooled data) then cases = (*n* × estimate of smoking prevalence) and non-cases = (*n* – cases). Cases = (439 × 0.160) = 70; non-cases = (439-70) = 369. Smoking prevalence over 2000-2007 therefore ~ Beta (70,369). The same method was used for a pooled estimate of smoking prevalence at the national level. Smoking prevalence over 2000-2007 in England for males aged 75-84 ~ Beta (283,2710)

National estimates of smoking prevalence were calculated in the start and final years of the model (2000 and 2007). Smoking prevalence in England for males aged 75-84 in 2000 ~ Beta (22,225); smoking prevalence in England for males aged 75-84 in 2007 ~ Beta (26,216)

- Increased relative risk attributable to smoking ~ Ersatz RR function (RR, SE ln(RR))

where RR was taken from the CPS-II study but modified to fit into the ten-year age bands used for IMPACT_SEC_ [57]. For males aged 75-84, RR for smoking = 1.31 with 95% CI (1.11,1.56). Using the 95% CI the SE of ln(RR) was calculated as follows:

ln(1.56)-ln(1.11)/(1.96×2) = 0.088

The relative risk for smoking in males aged 75-84 was therefore ~ Ersatz RR function (1.31, 0.088)

Putting all this together, ten runs in Ersatz gave the following estimates of each input variable randomly drawn from the relevant probability distributions from which we calculate DPPs from the change in smoking prevalence over 2000-2007 in males aged 75-84 in IMDQ5:

**Ten runs in Ersatz to calculate DPPs for change in smoking prevalence over 2000-2007 in males aged 75-84 in IMDQ5**

| **Run** | **Expected deaths** | **Pooled % smoke_IMD_** | **Pooled % smoke_England_** | **Relative risk** | **% smoke in 2000_Eng_** | **% smoke in 2007_Eng_** | **DPPs†** |
| --- | --- | --- | --- | --- | --- | --- | --- |
|  | **Probability distributions** | | | | | | |
|  | **Poisson** | **Beta** | **Beta** | **RR** | **Beta** | **Beta** |  |
|  | **Col A** | **Col B** | **Col C** | **Col D** | **Col E** | **Col F** |  |
| 1 | 4258 | 0.141 | 0.103 | 1.072 | 0.095 | 0.127 | -13.3 |
| 2 | 4207 | 0.175 | 0.092 | 1.231 | 0.082 | 0.101 | -33.2 |
| 3 | 4294 | 0.169 | 0.097 | 1.461 | 0.089 | 0.096 | -23.5 |
| 4 | 4130 | 0.161 | 0.092 | 1.377 | 0.080 | 0.118 | -94.5 |
| 5 | 4294 | 0.154 | 0.093 | 1.169 | 0.108 | 0.083 | 28.7 |
| 6 | 4259 | 0.169 | 0.100 | 1.337 | 0.067 | 0.112 | -101.4 |
| 7 | 4251 | 0.205 | 0.098 | 1.094 | 0.069 | 0.131 | -50.4 |
| 8 | 4290 | 0.169 | 0.106 | 1.371 | 0.095 | 0.092 | 6.3 |
| 9 | 4310 | 0.135 | 0.094 | 1.299 | 0.079 | 0.093 | -25.1 |
| 10 | 4402 | 0.151 | 0.090 | 1.158 | 0.050 | 0.100 | -56.2 |
| **Point estimate** | **4236** | **0.160** | **0.095** | **1.31** | **0.089** | **0.108** | **-38** |

† Intermediate steps in calculating DPPs as follows:

PARF using pooled data (IMD) = (B × (D-1))/(B × (D-1)+1);

PARF using pooled data (England) = (C × (D-1))/(C × (D-1)+1).

SEC gradient calculated as the ratio of these two quantities.

PARF in 2000 (England) = (E × (D-1))/(E × (D-1)+1);

PARF in 2007 (England) = (F × (D-1))/(F × (D-1)+1)

DPPs = A × ((PARF in 2000 (England) × SEC gradient) - ((PARF in 2007 (England) × SEC gradient)

*Uncertainty intervals for binary risk factor DPPs*

Within each run, DPPs were calculated for all age-sex-IMD groups and were then summed within each of the three binary risk factors. 95% uncertainty intervals from the set of 1000 runs (2.5^th^ and 97.5^th^ percentiles) were extracted for each risk factor total. The 95% uncertainty intervals were then scaled down using an overall correction for cumulative risk reduction (See Section 1.3 and Table D).

**3 Uncertainty analysis for change in continuous risk factors**

DPPs attributable to change in mean levels of systolic blood pressure (SBP) over 2000-2007 were calculated as follows:

***DPPs = expected CHD deaths in 2007 (had mortality rates in 2000 remained constant) × absolute risk factor reduction between 2000 and 2007 × regression coefficient exponentiated***

For continuous risk factors (SBP, total cholesterol, fruit and vegetable consumption, and BMI) we used the Poisson (expected deaths) and Normal probability distributions (mean risk factor levels and the beta coefficients quantifying the change in CHD mortality resulting from a one-unit absolute change in risk factor level between 2000 and 2007). The input values for the uncertainty analysis were as follows:

- Expected deaths ~ Poisson (population in 2007 × CHD mortality rates in 2000) ~ Poisson (4236)
- Estimates of SBP levels ~ Normal (mean, SE mean)

For males aged 75-84 in IMDQ5 mean SBP levels using all Health Survey for England data over the time period 2000 to 2007 ~ Normal (mean = 143.1 mmHg, SE = 1.51). The SE was estimated in Stata Version 11.1 to account for the complex survey design. Likewise, for males aged 75-84 in England mean SBP levels over 2000-2007 ~ Normal (mean = 141.0, SE = 0.49). The SEC gradient in mean SBP was computed as the ratio of these two quantities.

National estimates for 2000 and 2007 were used to estimate absolute change in SBP over the seven year period. In 2000, mean SBP for males aged 75-84 in England was ~ Normal (mean = 141.9, SE = 1.39); for 2007 mean SBP ~ Normal (mean = 135.8, SE = 1.48).

- Beta coefficients ~ Normal (mean, SE)

where the beta coefficient for SBP (on the logarithmic scale) taken from the PSC study for males aged 75-84 was -0.0212 with 95% CI (-0.0170,-0.0255) [53]. Using the 95% CIs the SE of the beta coefficient was calculated as follows:

(-0.0170) - (-0.0255)/(1.96×2) = 0.0022

The beta coefficient for SBP for males aged 75-84 in IMDQ5 was therefore ~ Normal (mean = -0.0212, SE = 0.0022).

Putting all this together, ten runs in Ersatz gave the following estimates of each input variable drawn from the relevant probability distributions from which we calculated DPPs from the absolute change in mean SBP over 2000-2007 in males aged 75-84 in IMDQ5:

**Ten runs in Ersatz to calculate DPPs for change in mean SBP levels over 2000-2007 in males aged 75-84 in IMDQ5**

| **Run** | **Expected deaths** | **Pooled SBP_IMD_** | **Pooled SBP_England_** | **Beta** | **SBP in 2000_Eng_** | **SBP in 2007_Eng_** | **DPPs†** |
| --- | --- | --- | --- | --- | --- | --- | --- |
|  | **Probability distributions** | | | | | | |
|  | **Poisson** | **Normal** | **Normal** | **Normal** | **Beta** | **Beta** |  |
|  | **Col A** | **Col B** | **Col C** | **Col D** | **Col E** | **Col F** |  |
| 1 | 4168 | 142.7 | 141.8 | -0.022 | 138.0 | 133.9 | 366.1 |
| 2 | 4149 | 146.2 | 142.1 | -0.018 | 142.8 | 136.3 | 476.0 |
| 3 | 4075 | 143.5 | 141.7 | -0.021 | 143.0 | 134.0 | 697.5 |
| 4 | 4225 | 144.2 | 140.6 | -0.019 | 139.8 | 136.7 | 256.8 |
| 5 | 4240 | 142.8 | 140.7 | -0.021 | 140.4 | 137.0 | 301.5 |
| 6 | 4194 | 143.8 | 141.8 | -0.021 | 140.8 | 134.9 | 502.2 |
| 7 | 4234 | 144.1 | 140.5 | -0.025 | 144.4 | 134.0 | 1012.8 |
| 8 | 4126 | 142.0 | 141.9 | -0.023 | 141.3 | 132.2 | 778.0 |
| 9 | 4291 | 145.3 | 141.7 | -0.019 | 140.2 | 135.2 | 389.1 |
| 10 | 4264 | 141.5 | 140.8 | -0.025 | 138.4 | 137.6 | 84.0 |
| **Point estimate** | **4236** | **143.1** | **141.0** | **-0.0212** | **141.9** | **135.8** | **524** |

† Intermediate steps in calculating DPPs as follows:

SEC gradient calculated as B/C.

Absolute change in mean SBP calculated as (E × SEC gradient) – (F × SEC gradient)

DPPs = (1-exp(D × (absolute change in mean SBP))) × A

*Uncertainty intervals for continuous risk factor DPPs*

Within each run, DPPs were calculated for all age-sex-IMD groups and were then summed within each of the four continuous risk factors. For systolic blood pressure and total cholesterol we then subtracted the DPPs calculated in the treatment (primary prevention) component of the model from the DPPs calculated in the risk factor component (See Section 1.4.3). 95% uncertainty intervals were then calculated from the set of 1000 runs (taken from the 2.5^th^ and 97.5^th^ percentiles) for the four continuous risk factor DPP totals. These were then scaled down using an overall correction for cumulative risk reduction.

*Uncertainty intervals for total risk factor contribution DPPs*

Within each run, the total risk factor contribution to model DPPs was obtained by summation of seven risk factor DPPs totals (within each age-sex-IMD group). A 95% uncertainty interval for the overall risk factor contribution was obtained from the set of 1000 runs by taking the 2.5^th^ and 97.5^th^ percentiles. These lower and upper limits were then scaled down using the correction for cumulative risk reduction.

*Uncertainty intervals for total model DPPs (changes in treatment uptake and risk factors)*

Within each run, we also calculated an estimate of the total model DPPs by summation of the treatment contribution DPPs and the risk factor contribution DPPs *after* adjustment for cumulative risk reduction. The standard deviation of the estimated total model DPPs over the set of 1000 runs was extracted for England and each deprivation quintile. The 95% uncertainty intervals for total model DPPs were then obtained as follows:

Lower limit = estimate – (1.96 × standard deviation)

Upper limit = estimate + (1.96 × standard deviation)

# Table N. Assumptions and overlap adjustments used in the IMPACT_SEC_ model

To avoid double counting of patients treated for two or more conditions within the year (e.g. heart failure develops within 1 year after myocardial infarction in approximately 30% of survivors) we quantified overlaps between different groups and made appropriate adjustments. Overlap adjustments were based on estimating the patient counts for each condition separately, and the counts of patients with two or more of the target conditions. The latter, or the probabilities of events intersecting, were then used to adjust single patient counts into unique non-overlapping counts by condition.

We constructed nine non-overlapping patient groups. Following the basic logic of the natural history of coronary disease, patients recorded as having two or more conditions were allocated to the condition that was further along the disease pathway. For instance, if an individual had both chronic angina and heart failure, they were allocated to heart failure.

**Potential overlaps between patient groups with chronic CHD**

**
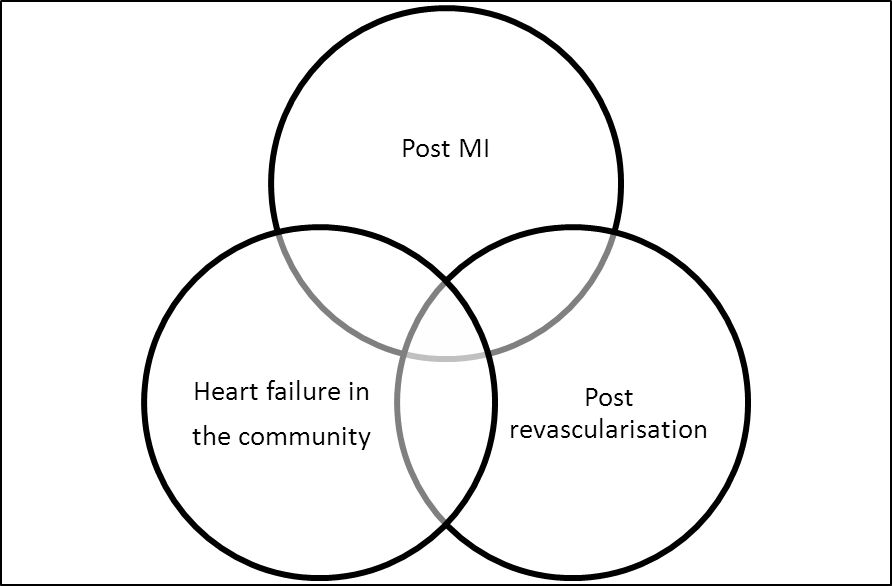
**

Therefore, to avoid double counting, potential overlaps between different groups of patients were identified and appropriate adjustments made by subtracting one group from another. For instance, we can subtract the number of heart failure patients treated in hospital from the total number of heart failure patients in the community (because community heart failure patients could be admitted to hospital on one or more occasions). The set of overlap adjustments made for the IMPACT_SEC_ model is presented in Box 1. Patient overlaps for the final year of the model are shown in Figure N.1.

**Box 1: Main assumptions and overlap adjustments used in the IMPACT_SEC_ model**

| **Treatment category** | **Assumptions and overlap adjustments** | **Justification** |
| --- | --- | --- |
| **Post-AMI** | Assume 30% already counted in the heart failure in community group. | Weir (2006) [63] |
| **Post-CABG** | Assume two-thirds had AMI, already counted as post AMI | Unal (2004) [1] |
| **Post-angioplasty** | - Assume 50% had prior AMI, already counted as post AMI - Assume 25% also had CABG, thus already counted as post CABG - Assume 25% had prior PTCA, i.e. repeat procedures, already counted | Unal (2004) [1] |
| **Angina in the community** | Start with the total numbers with angina in the community (without MI) based on GPRD prevalence. Then deduct persons counted elsewhere:   - Persons already treated for unstable angina in hospital - 50% of those in the heart failure in the community group - 2/3 of those receiving secondary prevention post CABG/post PTCA | Capewell (2000) [3] |
| **Heart failure in the community** | Based on GPRD prevalence.   - Assume 50% of heart failure is due to CHD - Deduct persons treated for severe heart failure in the hospital (already counted) | NHANES 1999-2000 |
| **Fall in population blood pressure** | Estimate the number of DPPs by hypertension treatment   - Then subtract this from the total DPPs attributed to the secular fall in population blood pressure | Capewell (1999) [4]  Capewell (2000) [4] |
| **Fall in population total cholesterol** | Estimate the number of DPPs by cholesterol lowering medication   - Then subtract this from the total DPPs attributed to the secular fall in population cholesterol |  |
| AMI denotes acute myocardial infarction, CABG coronary artery bypass graft surgery, CHD coronary heart disease, DPPs deaths prevented or postponed, GPRD General Practice Research Database | | |

**Figure N.1: Patient overlaps for IMPACT_SEC_ (2007) *(Page 1 of 2)***

MI denotes myocardial infarction; STEMI ST-segment elevation; NSTE-ACS non-ST segment elevation; CHD Coronary heart disease; CABG Coronary artery bpass grafting; PTCA Percutaneous transluminal coronary angioplasty. Figures in bold represent patient numbers used in IMPACT_SEC_ model (i.e. minus relevant overlaps).

**Hospital admissions**

MI (51,755)

**STEMI (20,702)**

**NSTE-ACS (91,288)**

Unstable angina (60,235)

Heart failure (49,248)

NOT due to CHD (excluded) (24,624)

**Heart failure due to CHD (24,624)**

**Primary prevention**

**(population ≈ 35.2 million)**

**Statins for hypercholesterolemia (3,159,505)**

**Anti-hypertensive medication (4,754,711)**

**Figure N.1: Patient overlaps for IMPACT_SEC_ (2007) – *(Page2 of 2)***

AMI denotes acute myocardial infarction; CHD Coronary heart disease; HF heart failure; CABG Coronary artery bpass grafting; PTCA Percutaneous transluminal coronary angioplasty. † MI & unstable angina admissions having PTCA/CABG in 2007 subtracted from revascularisation counts. Figures in bold represent patient numbers used in IMPACT_SEC_ model (i.e. minus relevant overlaps).

**Secondary prevention**

Post MI

TOTAL

(807,988)

**MI, no HF (565,592)**

Overlap with HF in community (807,988 × 0.3)

Angina, no MI

TOTAL

(1,168,737)

HF in the community

TOTAL

(394,789)

Post CABG survivors

TOTAL

(122,708)

Post PTCA survivors

TOTAL

(252,540)

**Angina, no MI (984,807)**

Overlap with HF in community (172,770 × 0.5)

Overlap with post revasc (40,903 + 71,027) × (1/3)

Admissions for UA (60,235)

**CHD related HF (172,770)**

Not CHD (394,789 × 0.5)

NOT INCLUDED

Admissions for CHD related HF (24,624)

**Survivors†, no AMI, over 7 year period**

**(40,903)**

Overlap with Post MI

(122,708 × (2/3))

**Survivors†, no AMI, over 7 year period**

**(71,027)**

Overlap with Post MI (252,540 × 0.5)

Overlap with CABG (252,540 × 0.5 × 0.75)

Repeat PTCA procedures

(252,540 × 0.5 × 0.75 × 0.75)

# Table O. ‘Fixed gradients’ for measuring risk factor change between two time points for deprivation quintiles

The annual sample size of the Health Survey for England (HSfE), roughly 14,000 adults aged 16 years and over, was not large enough to provide accurate/precise estimates of risk factor levels, and hence rates of change over time by age, sex, and deprivation quintiles (70 groups in total). We considered three options for estimating risk factor change as key inputs into the regression and PARF deaths prevented or postponed calculations:

1. Option 1: using single-year estimates for the base and final year (2000 and 2007 respectively). Both surveys, however, were half the usual adult size due to boost samples for population sub-groups.
2. Option 2: using estimates based on three-year averages with pooled 1999-2001 survey data for the base year (2000) and 2005-7 for the final year (2006 as the mid-year). Estimates of risk factor change over 2000-6 were scaled up by a factor of 8/7 (i.e. number of years between 2000 and 2007 divided by the observed number of years).
3. Option 3: the ‘fixed gradient approach’ (discussed in detail below).

The fixed gradient approach was based on the assumption that changes in pace and direction for each deprivation quintile were similar and therefore, most accurately measured by the overall national rates of change (across 14 age-sex groups). If this assumption holds, then relatively stable and plausible estimates for each quintile could be derived by scaling the national age-sex risk factor levels up or down using a fixed ratio/gradient.

The fixed gradient was derived by pooling together survey data for all available years from 2000 to 2007 to calculate risk factor estimates by age, sex, and deprivation quintiles. Then the pooled national estimate for 14 age-by-sex groups was set notionally to one, and the corresponding estimates for each deprivation quintile re-indexed to be below or above one (i.e. expressing the ratio of the deprivation quintile to national estimate). These index rates for each of the 70 breaks were then applied to the single year national estimates to derive the corresponding 70 risk factor levels for that year. The fixed gradient was applied to both the start and end years of the model. An illustrative example, using the population-attributable risk fraction (PARF), is set out below.

**EXAMPLE: Fixed gradient for change in smoking prevalence in men aged 45-54**

*Step 1*

Using the pooled 2000-7 HSfE data the national estimate of current smoking was 25.7% for men aged 45-54. Estimates by deprivation quintile ranged from 14.0% for men in the most affluent quintile (IMDQ1) to 46.5% in the most deprived (IMDQ5). The relative risk (RR) of smoking taken from the CPS-II study was 3.04 [57]. Using the smoking prevalence (P) and the RR (assumed the same across deprivation quintiles) we calculated the PARF for England as a whole and each deprivation quintile using the formula:

***PARF = [P × (RR - 1)]/[1 + P × (RR - 1)]***

*Applying step 1: Calculate the PARF gradient using 2000-*7 pooled survey data

| **Men 45-54** | **England**  **2000-7** | | **IMDQ1**  **2000-7** | **IMDQ2**  **2000-7** | **IMDQ3**  **2000-7** | **IMDQ4**  **2000-7** | **IMDQ5**  **2000-7** |
| --- | --- | --- | --- | --- | --- | --- | --- |
| **Proportion smokers (P)** | | 0.2569 | 0.1398 | 0.1949 | 0.2668 | 0.2882 | 0.4652 |
| **RR** | | 3.04 | 3.04 | 3.04 | 3.04 | 3.04 | 3.04 |
| **PARF** | | 0.3427 | 0.2258 | 0.2819 | 0.3510 | 0.3671 | 0.4846 |
| **Gradient in PARF** | | 1 | 0.659 | 0.823 | 1.024 | 1.071 | 1.414 |

The PARF calculated using pooled data at the national level was then set notionally to one, and the corresponding values for each deprivation quintile re-indexed to be below or above one. For example, the pooled gradient in the PARF for men aged 45-54 in Q1 was estimated to be 0.2258/0.3427 = 0.659.

*Step 2*

Using the HSfE data for the start and final year of the model we then derived the national PARF for 14 age-by-sex groups. The national PARF for men aged 45-54 based on prevalence (P) of 28.3% and RR of 3.04 in 2000 was 0.3662; a prevalence of 25.1% in 2007 gave a PARF of 0.3385.

*Applying step 2: Calculate the national PARF in base and final year*

| **Men 45-54** | **England**  **2000** | **England**  **2007** |
| --- | --- | --- |
| **Proportion smokers (P)** | 0.2832 | 0.2508 |
| **RR** | 3.04 | 3.04 |
| **PARF** | 0.3662 | 0.3385 |

*Step 3*

The fixed gradient (Step 1) was then applied to the national PARF (Step 2) to produce estimates of the PARF for each deprivation quintile, separately for the base and final years of the model. For example, for men aged 45-54 in Q1 the 2000 estimate of the PARF was equal to 0.3662 (national PARF) multiplied by the gradient (0.659), to give an estimate of 0.2413. The 2007 estimate was equal to 0.3385 (national PARF) multiplied by the fixed gradient (0.659), to give an estimate of 0.2230.

*Applying step 3: Estimate the PARF by deprivation quintiles for single years 2000 and 2007 using fixed gradient*

| **Men 45-54** | **England**  **2000-7** | **IMDQ1**  **2000-7** | **IMDQ2**  **2000-7** | **IMDQ3**  **2000-7** | **IMDQ4**  **2000-7** | **IMDQ5**  **2000-7** |
| --- | --- | --- | --- | --- | --- | --- |
| PARF 2000 | 0.3662 | 0.2413 | 0.3012 | 0.3751 | 0.3923 | 0.5179 |
| PARF 2007 | 0.3385 | 0.2230 | 0.2784 | 0.3467 | 0.3626 | 0.4787 |

*Step 4: Calculating the DPPs*

The formula for calculating DPPs using the change in PARF approach was as follows:

***Expected CHD deaths in 2007 (had mortality rates in 2000 remained constant) × difference between the PARF in 2000 and 2007***

***Expected CHD deaths in 2007 × (PARF_2000_ – PARF_2007_)***

*Applying step 4: Estimate the DPPs due to change in PARF between 2000 and 2007*

| **Men 45-54** | **England**  **2000-7** | **IMDQ1**  **2000-7** | **IMDQ2**  **2000-7** | **IMDQ3**  **2000-7** | **IMDQ4**  **2000-7** | **IMDQ5**  **2000-7** |
| --- | --- | --- | --- | --- | --- | --- |
| CHD mortality rate (2000)† | 0.9131 | 0.5434 | 0.6644 | 0.8177 | 1.1173 | 1.6448 |
| Population (2007) | 3284291 | 736444 | 700676 | 660481 | 611424 | 575266 |
| Aded deaths (2007) | 3035 | 400 | 466 | 540 | 683 | 946 |
| PARF 2000 | 0.3662 | 0.2413 | 0.3012 | 0.3751 | 0.3923 | 0.5179 |
| PARF 2007 | 0.3385 | 0.2230 | 0.2784 | 0.3467 | 0.3626 | 0.4787 |
| **DPPs in 2007** | **91**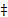 | **7** | **11** | **15** | **20** | **37** |

† Rate per 1000

‡ The total DPPs for England was based on the sum of the DPPs across the deprivation quintiles.

We tested all three options and selected Option 3 (‘fixed gradient’ approach). This method had the advantage of reducing the number of data breaks to a maximum of 14 (age by sex) for any single HSfE year and instead of discarding the survey information in the intermediate years, used the whole data series to improve and stabilise the 70 estimates. The disadvantage was that the assumption of a fixed gradient for each age-by-sex group remaining constant over time may not hold (e.g. the difference in risk factor level between a deprivation quintile and the national rate may be considerably larger in 2000 than in 2007). Furthermore, we concluded that substantial relative changes over such a short period were possible but unlikely.

# Reference List

1. Unal B, Critchley JA, Capewell S (2004) Explaining the decline in coronary heart disease mortality in England and Wales between 1981 and 2000. Circulation 109:1101-1107.

2. Ford ES, Ajani UA, Croft JB, Critchley JA, Labarthe DR, et al. (2007) Explaining the decrease in US deaths from coronary disease, 1980-2000. N Engl J Med 356:2388-2398. Supplementary Appendix available at (http://www.nejm.org/doi/suppl/10.1056/NEJMsa053935/suppl_file/nejm_ford_2388sa1.pdf).

3. Capewell S, Beaglehole R, Seddon M, McMurray J (2000) Explanation for the decline in coronary heart disease mortality rates in Auckland, New Zealand, between 1982 and 1993. Circulation 102:1511-1516.

4. Capewell S, Morrison CE, McMurray JJ (1999) Contribution of modern cardiovascular treatment and risk factor changes to the decline in coronary heart disease mortality in Scotland between 1975 and 1994. Heart 81:380-386.

5. Wijeysundera HC, Machado M, Farahati F, Wang X, Witteman W, et al. (2010) Association of temporal trends in risk factors and treatment uptake with coronary heart disease mortality, 1994-2005. JAMA 303:1841-1847.

6. Yeh RW, Sidney S, Chandra M, Sorel M, Selby JV, et al. (2010) Population trends in the incidence and outcomes of acute myocardial infarction. N Engl J Med 362:2155-2165.

7. Dauchet L, Amouyel P, Hercberg S, Dallongeville J (2006) Fruit and vegetable consumption and risk of coronary heart disease: A meta-analysis of cohort studies. J Nutr 136:2588-2593.

8. Porta M (2008) A dictionary of epidemiology. Oxford University Press.

9. Ezatti M, Lopez AD, Rodgers A, Murray CJL, editors (2004) Comparative quantification of risk. Global and regional burden of disease attributable to selected major risk factors. Geneva: World Health Organization.

10. Roglic G, Unwin N (2010) Mortality attributable to diabetes: estimates for the year 2010. Diabetes Res Clin Pract 87:15-19.

11. Huxley R, Barzi F, Woodward M (2006) Excess risk of fatal coronary heart disease associated with diabetes in men and women: meta-analysis of 37 prospective cohort studies. BMJ 332:73-78.

12. Danaei G, Ding EL, Mozaffarian D, Taylor B, Rehm J, et al. (2009) The preventable causes of death in the United States: comparative risk assessment of dietary, lifestyle, and metabolic risk factors. PLoS Med 6(4): e1000058.

13. Tobias M, Taylor R, Yeh LC, Huang K, Mann S, et al. (2008) Did it fall or was it pushed? The contribution of trends in established risk factors to the decline in premature coronary heart disease mortality in New Zealand. Aust N Z J Public Health 32:117-125.

14. Taylor R, Dobson A, Mirzaei M (2006) Contribution of changes in risk factors to the decline of coronary heart disease mortality in Australia over three decades. Eur J Cardiovasc Prev Rehabil 13:760-768.

15. Dobson A, McElduff P, Heller R, Alexander H, Colley P, et al. (1999) Changing patterns of coronary heart disease in the Hunter region of New South Wales, Australia. J Clin Epidemiol 52:761-771.

16. Yusuf S (2002) Two decades of progress in preventing vascular disease. Lancet 360:2-3.

17. Wald NJ, Law MR (2003) A strategy to reduce cardiovascular disease by more than 80%. BMJ 326:1419-1424.

18. Mant J, Hicks N (1995) Detecting differences in quality of care - the sensitivity of measures of process and outcome in treating acute myocardial-infarction. BMJ 311:793-796.

19. Noble M, mcLennan D, Wilkinson K et al (2007). The English Indices of Deprivation 2007. Department for Communities and Local Government.

20. Adams J, White M (2006) Removing the health domain from the Index of Multiple Deprivation 2004 - effect of measured inequalities in census measure of health. J Pub Health 28:379-383.

21. DH Vascular Programme Team (2008). Treatment of heart attack National Guidance. Final report of the National Infarct Angioplasty Project (NIAP).

22. National Audit Team British Heart Foundation, University of York (2008). The National Audit of Cardiac Rehabilitation. Annual Statistical report 2008. London: British Heart Foundation.

23. Nicol ED, Fittall B, Roughton M, Cleland JGF, Dargie H, et al. (2008) NHS heart failure survey: a survey of acute heart failure admissions in England, Wales and Northern Ireland. Heart 94:172-177.

24. Craig R, Mindell J (2008) Health Survey for England 2006. London, United Kingdom: The Information Centre.

25. Estess JM, Topol EJ (2002) Fibrinolytic treatment for elderly patients with acute myocardial infarction. Heart 87:308-311.

26. ISIS-2 (Second international study of infarct survival) collaborative group (1988) Randomised trial of intravenous streptokinase, oral aspirin, both, or neither among 17 187 cases of suspected acute myocardial infarction: ISIS-2. Lancet 8607:349-360.

27. Yusuf S, Zucker D, Peduzzi P, Fisher LD, Takaro T, et al. (1994) Effect of coronary-artery bypass graft-surgery on survival - overview of 10-year results from randomized trials by the coronary-artery bypass graft-surgery trialists collaboration. Lancet 344:563-570.

28. Keeley EC, Boura JA, Grines CL (2003) Primary angioplasty versus intravenous thrombolytic therapy for acute myocardial infarction: a quantitative review of 23 randomised trials. Lancet 361:13-20.

29. Freemantle N, Cleland J, Young P, Mason J, Harrison J (1999) Beta blockade after myocardial infarction: systematic review and meta regression analysis. BMJ 318:1730-1737.

30. Ace Inhibitor Myocardial Infarction Collaborative Group (1998) Indications for ACE inhibitors in the early treatment of acute myocardial infarction: systematic overview of individual data from 100 000 patients in randomized trials. Circulation 97:2202-2212.

31. Chen ZM, Jiang LX, Chen YP, Xie JX, Pan HC, et al. (2005) Addition of clopidogrel to aspirin in 45,852 patients with acute myocardial infarction: randomised placebo-controlled trial. Lancet 366:1607-1621.

32. Sabatine MS, Cannon CP, Gibson CM, Lopez-Sendon JL, Montalescot G, et al. (2005) Addition of clopidogrel to aspirin and fibrinolytic therapy for myocardial infarction with ST-segment elevation. N Engl J Med 352:1179-1189.

33. Tunstall-Pedoe H, Bailey L, Chamberlain DA, Marsden AK, Ward ME, et al. (1992) Survey of 3765 cardiopulmonary resuscitations in British hospitals (the BRESUS Study): methods and overall results. BMJ 304:1347-1351.

34. Nadkarni VM, Larkin GL, Peberdy MA, Carey SM, Kaye W, et al. (2006) First documented rhythm and clinical outcome from in-hospital cardiac arrest among children and adults. JAMA 295:50-57.

35. Antithrombotic Trialists' Collaboration (2002) Collaborative meta-analysis of randomised trials of antiplatelet therapy for prevention of death, myocardial infarction, and stroke in high risk patients. BMJ 324:71-86.

36. Oler A, Whooley MA, Oler J, Grady D (1996) Adding heparin to aspirin reduces the incidence of myocardial infarction and death in patients with unstable angina. A meta-analysis. JAMA 276:811-815.

37. Boersma E, Harrington RA, Moliterno DJ, White H, Theroux P, et al. (2002) Platelet glycoprotein IIb/IIIa inhibitors in acute coronary syndromes: a meta-analysis of all major randomised clinical trials. Lancet 359:189-198.

38. Fox KAA, Poole-Wilson P, Clayton TC, Henderson RA, Shaw TRD, et al. (2005) 5-year outcome of an interventional strategy in non-ST-elevation acute coronary syndrome: the British Heart Foundation RITA 3 randomised trial. Lancet 366:914-920.

39. Yusuf S, Zhao F, Mehta SR, Chrolavicius S, Tognoni G, et al. (2001) Effects of clopidogrel in addition to aspirin in patients with acute coronary syndromes without ST-segment elevation. N Engl J Med 345:494-502.

40. Flather MD, Yusuf S, Kober L, Pfeffer M, Hall A, et al. (2000) Long-term ACE-inhibitor therapy in patients with heart failure or left-ventricular dysfunction: a systematic overview of data from individual patients. ACE-Inhibitor Myocardial Infarction Collaborative Group. Lancet 355:1575-1581.

41. Hulten E, Jackson JL, Douglas K, George S, Villines TC (2006) The effect of early, intensive statin therapy on acute coronary syndrome: a meta-analysis of randomized controlled trials. Arch Intern Med 166:1814-1821.

42. Anand SS, Yusuf S (1999) Oral anticoagulant therapy in patients with coronary artery disease: a meta-analysis. JAMA 282:2058-2067.

43. Taylor RS, Brown A, Ebrahim S, Jolliffe J, Noorani H, et al. (2004) Exercise-based rehabilitation for patients with coronary heart disease: systematic review and meta-analysis of randomized controlled trials. Am J Med 116:682-692.

44. Boden WE, O'Rourke RA, Teo KK, Hartigan PM, Maron DJ, et al. (2007) Optimal medical therapy with or without PCI for stable coronary disease. N Engl J Med 356:1503-1516.

45. Wilt TJ, Bloomfield HE, MacDonald R, Nelson D, Rutks I, et al. (2004) Effectiveness of statin therapy in adults with coronary heart disease. Arch Intern Med 164:1427-1436.

46. Al-Mallah MH, Tleyjeh IM, Abdel-Latif AA, Weaver WD (2006) Angiotensin-converting enzyme inhibitors in coronary artery disease and preserved left ventricular systolic function: a systematic review and meta-analysis of randomized controlled trials. J Am Coll Cardiol 47:1576-1583.

47. Shibata MC, Flather MD, Wang DL (2001) Systematic review of the impact of beta blockers on mortality and hospital admissions in heart failure. Eur J Heart Fail 3:351-357.

48. Pitt B, Zannad F, Remme WJ, Cody R, Castaigne A, et al. (1999) The effect of spironolactone on morbidity and mortality in patients with severe heart failure. N Engl J Med 341:709-717.

49. Kjekshus J, Apetrei E, Barrios V, Böhm M, Cleland JG, et al. (2007) Rosuvastatin in older patients with systolic heart failure. N Engl J Med 357:2248-2261.

50. Tavazzi L, Maggioni AP, Marchioli R, Barlera S, Franzosi MG, et al. (2008) Effect of rosuvastatin in patients with chronic heart failure (the GISSI-HF trial): a randomised, double-blind, placebo-controlled trial. Lancet 372:1231-1239.

51. Law M, Wald N, Morris J (2003) Lowering blood pressure to prevent myocardial infarction and stroke: a new preventive strategy. Health Technol Assess 7:1-94.

52. Pignone M, Phillips C, Mulrow C (2000) Use of lipid lowering drugs for primary prevention of coronary heart disease: meta-analysis of randomised trials. BMJ 321:983-986.

53. Lewington S, Clarke R, Qizilbash N, Peto R, Collins R (2002) Age-specific relevance of usual blood pressure to vascular mortality: a meta-analysis of individual data for one million adults in 61 prospective studies. Lancet 2002 360:1903-1913.

54. Lewington S, Whitlock G, Clarke R, Sherliker P, Emberson J, et al. (2007) Blood cholesterol and vascular mortality by age, sex, and blood pressure: a meta-analysis of individual data from 61 prospective studies with 55000 vascular deaths. Lancet 370:1829-1839.

55. Bogers RP, Hoogenveen RT, Boshuizen H, Woodward M, Knekt P, et al. (2006) Overweight and obesity increase the risk of coronary heart disease: A pooled analysis of 30 prospective studies. European Journal of Epidemiology 21(Supplement):107.

56. James WPT, Jackson-Leach R, Mhurchu CN, Kalamara E, Shayeghi M, et al (2004). Overweight and obesity (high body mass index). In: Ezatti M, Lopez AD, Rodgers A, Murray CJL, editors. Comparative quantification of risk. Global and regional burden of disease attributable to selected major risk factors. Volume 1 ed. Geneva: World Health Organization. pp. 497-596.

57. Ezzati M, Henley SJ, Thun MJ, Lopez AD (2005) Role of smoking in global and regional cardiovascular mortality. Circulation 112:489-497.

58. Bull F, Armstrong TP, Dixon T, Ham S, Neiman A, et al. (2004) Physical inactivity. In: Ezatti M, Lopez AD, Rodgers A, Murray CJL, editors. Comparative quantification of risk. Global and regional burden of disease attributable to selected major risk factors. Volume 1 ed. Geneva: World Health Organization. pp. 729-881.

59. Joubert J, Norman R, Lambert EV, Groenewald P, Schneider M, et al. (2007) Estimating the burden of disease attributable to physical inactivity in South Africa in 2000. S Afr Med J 97:725-731.

60. Hu G, Qiao Q, Tuomilehto J, Balkau B, Borch-Johnsen K, Pyorala K, for the DECODE Study Group (2004) Prevalence of the metabolic syndrome amd its relation to all-cause and cardiovascular mortality in nondiabetic European men and women. Arch Intern Med 164:1066-1076.

61. Morrish NJ, Wang SL, Stevens LK, Fuller JH, Keen H (2001) Mortality and causes of death in the WHO Multinational Study of Vascular Disease in Diabetes. Diabetologia 44 Suppl 2:S14-21.

62. Barendregt JJ (2010) The effect size in uncertainty analysis. Value in Health 4:388-391.

63. Weir RAP, McMurray JJV, Velazquez EJ (2006) Epidemiology of heart failure and left ventricular systolic dysfunction after acute myocardial infarction: prevalence, clinical characteristics, and prognostic importance. Am J Cardiol 97:13F-25F.
